# The versatile world of cumulene chemistry

**DOI:** 10.1039/d5sc04462f

**Published:** 2025-11-27

**Authors:** Abhishek Pareek, Yu Qiu, Matthew A. Johnson, Rik R. Tykwinski, Przemysław Gaweł

**Affiliations:** a Institute of Organic Chemistry, Polish Academy of Sciences Kasprzaka 44/52 01-224 Warsaw Poland pgawel@icho.edu.pl p.gawel@cent.uw.edu.pl; b Department of Chemistry, University of Alberta Edmonton Alberta T6G 2G2 Canada tykwinsk@ualberta.ca

## Abstract

Despite a history spanning over a century, cumulenes are often relegated to the realm of perceived curiosities rather than practical synthetic intermediates as a result of their high intrinsic reactivity. In this review, we bridge the gap between the known synthetic strategies for these “exotic” molecules and the potential of their reactivity. By surveying both even and odd [*n*]cumulenes, paying particular attention to the most accessible [3]cumulenes, alongside more limited but instructive examples of [4]- and [5]cumulenes, we demonstrate that these sp-hybridized frameworks offer exceptional synthetic versatility. Indeed, cumulenes can function effectively as nucleophiles, electrophiles, and dienophiles, enabling cyclooligomerization, cycloaddition, organometallic coupling, and other transformations. We describe how these reactions utilize the “naked” sp-hybridized carbon atoms of the cumulene and their substantial internal energy to access structurally diverse products that would otherwise be challenging or even impossible to obtain using more traditional routes. In doing so, we aim to showcase their potential in organic synthesis and highlight the opportunities they present for constructing novel molecular architectures. By reframing cumulenes as valuable synthetic building blocks, rather than mere curiosities, this review hopes to persuade chemists to incorporate these intriguing scaffolds more broadly into modern organic synthesis.

## Abstract 2

Cumulenes, long regarded as exotic curiosities, reveal remarkable versatility as nucleophiles, electrophiles, and dienophiles, enabling diverse transformations and offering powerful tools for modern organic synthesis.

## Graphical abstract

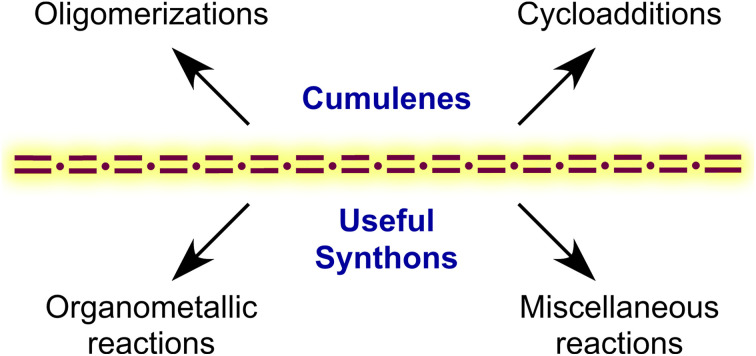

## Introduction

**Fig. 1:**
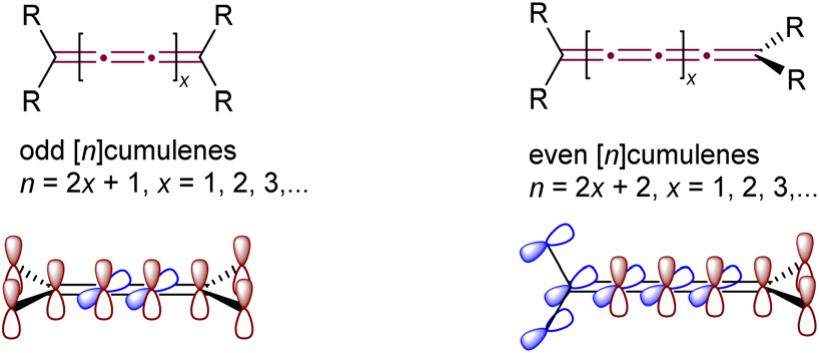
Classification of cumulenes and schematic depiction of π-orbital systems.

Cumulenes, distinguished by their ‘naked’ sp-hybridized carbon atoms and high internal energy, not only pose significant synthetic challenges but also yield numerous exciting reactions, balancing these challenges with rewarding chemical diversity. As defined by IUPAC, cumulenes are linear chains of carbon atoms joined by three or more consecutive double bonds.^1^ The structure of cumulenes, as predicted by van't Hoff in 1875, varies with the carbon chain length (Fig. 1).^2,3^ [*n*]Cumulenes with an even number of sp-carbon atoms (and thus an odd number of double bonds, with odd *n*) are planar, with all substituents lying in a plane together with the skeleton of the cumulene and can exhibit *E*/*Z* isomerism. Conversely, [*n*]cumulenes with an even number of double bonds (even *n* and odd number of sp-carbon atoms) have substituents in two perpendicular planes intersecting at the cumulene axis, giving axial chirality in cases with the appropriate substitution. Because of these structural distinctions, cumulenes bearing an odd *versus* even number of double bonds exhibit substantially different properties and reactivity.

Early interest in [*n*]cumulenes was driven by their unique structure and potential applications as carbon-rich materials.^4–7^ The initial synthesis of cumulenes, *i.e.*, tetraaryl[3]cumulenes, was achieved by Brand in the early 1920s through HCl elimination from 2,3-dichloro-but-2-enes.^8,9^ The relationship of cumulenes to sp-hybridized carbon allotropes, particularly carbyne and cyclocarbons, gained equal significance over time.^10–13^ This stemmed from [*n*]cumulenes, together with polyynes, representing the two fundamental boundary structures of these allotropes, highlighting their role in understanding the nature of carbyne.^10–12^ Significant progress in synthetic methodologies toward carbyne has led to the development of record-breaking [9]cumulenes and a 68-carbon atom-long polyyne,^14–18^ along with cyclocarbons that exhibited a cumulenic structure in their smaller, aromatic counterparts.^13,19–24^ These remarkable achievements have been instrumental in fostering our understanding of cumulenes.

The latter half of the 20th century saw a significant expansion in cumulenic structures and synthetic methods, which have been reviewed elsewhere.^25^ In contrast, the rich and diverse reactivity of [*n*]cumulenes remains dispersed throughout the literature, with no comprehensive summary available. Conversely, polyynes, similar to [*n*]cumulenes, also contain ‘naked carbon atoms’ with relatively high internal energy^26^ and are well recognized as valuable building blocks in organic synthesis.^27,28^

**Fig. 2:**
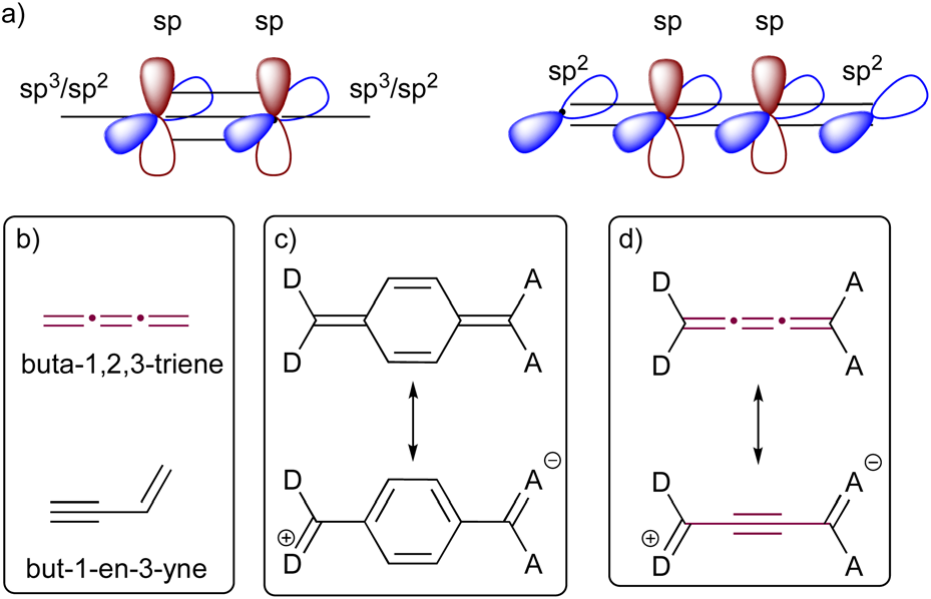
Proacetylenic character of cumulenes. (a) Comparison of π-orbitals in cumulene and acetylene; (b) structures of but-3-en-1-yne and buta-1,2,3-triene; (c) schematic representation of proaromaticity in quinones; (d) schematic representation of proacetylenic character in cumulenes.^29–31^

The bonding of sp-hybridized carbon atoms in odd [*n*]cumulenes shows an alternating pattern of short and long bonds, and as a result, they exhibit electronic characteristics remarkably similar to those of polyynes (Fig. 2). This proacetylenic character of cumulenes is manifested in their structural composition, intrinsic properties, and most notably, their distinctive reactivity patterns.^29^ This concept is somewhat analogous to the proaromaticity observed in quinoidal chromophores, in which aromatic ring stabilization is the driving force for charge separation (Fig. 2).^30^ Topological resonance energy (TRE) theory, comprehensively elucidated by Chauvin and coworkers, offers insight into this phenomenon.^31^ According to TRE theory, but-1-en-3-yne is more stable in comparison to its constitutional cumulenic isomer, buta-1,2,3-triene (Fig. 2).^31^ The degree to which the central C=C bond of a [3]cumulene unit resembles a triple bond is dependent on the electronic character of terminal groups. The polarization induced by substituting a cumulene with electron-donating (D) and electron-accepting (A) groups at the terminal positions leads to a pronounced bond-length alternation (BLA, the difference in bond lengths between the two most central bonds of an odd cumulene).^32–34^ Likewise, formal replacement of alkyl endgroups of an odd [*n*]cumulene with aryl groups leads to a increased BLA due to increased conjugation.^15^

In this review, we aim to bridge the gap between [*n*]cumulenes as structural curiosities and useful building blocks toward convincing the organic chemistry community that [*n*]cumulenes offer access to chemical space that is impossible with other types of transformations. We focus in particular on the reactivity of the most common [3]cumulenes while also covering examples involving the more rarely encountered [4]- and [5]cumulenes. Due to their unique structure, [*n*]cumulenes exhibit remarkable synthetic versatility, participating in reactions as nucleophiles, electrophiles, and dienophiles. They engage in a wide array of transformations, including oxidation, hydrogenation, halogenation, cycloaddition, metalation, metal complexation, and sulfurization. This review presents a comprehensive account of the reactivity of [*n*]cumulenes, beginning with a historical overview and followed by dedicated sections on cyclooligomerizations, cycloadditions, organometallic transformations, and other relevant reaction types. Given the distinct reactivity patterns of odd- and even-numbered cumulenes, these classes are discussed separately.

## Odd cumulenes

### Early cumulene reactions

**Scheme 1:**
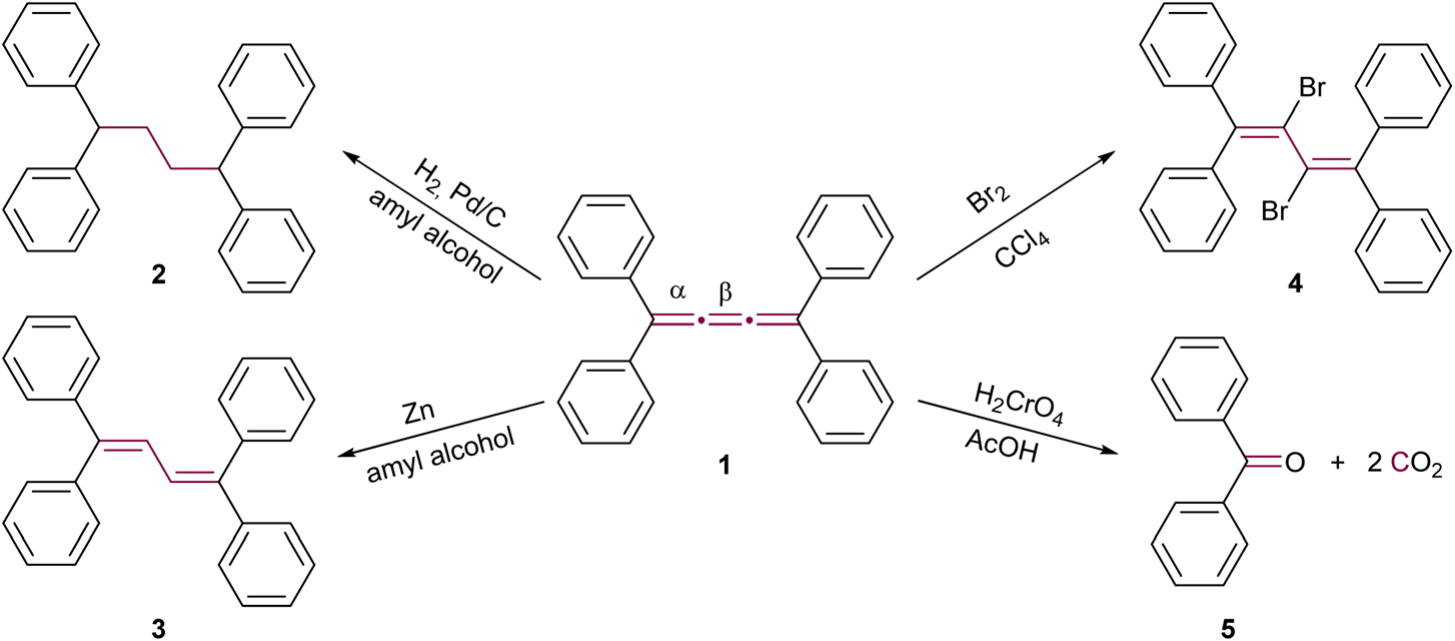
Early reactions of [3]cumulenes, as reported by Brand in 1921, using the example of tetraphenyl[3]cumulene 1.^8,9,33,35–38^

The initial reactions involving cumulenes, primarily hydrogenations and oxidations, were demonstrated by Brand alongside the first report of the synthesis of an [*n*]cumulene (Scheme 1).^8,9^ The hydrogenation of tetraphenyl[3]cumulene 1 in the presence of palladium on carbon leads to the complete saturation of the [3]cumulene moiety, yielding 1,1,4,4-tetraphenylbutane 2. A milder reduction employing zinc gives 1,1,4,4-tetraphenylbuta-1,3-diene 3 (Scheme 1). Electrophilic addition reactions with halogens (Br_2_, I_2_, *etc*.) predominantly occur at the central bond (β) of the [3]cumulene, forming 2,3-dihalobutadienes such as 4.^33,35^ Notably, bromination may also target a terminal double bond (α) or instigate a domino reaction, leading to the formation of cyclic structures (*vide infra*).^36–38^ Furthermore, oxidation of 1 by chromic acid results in the production of benzophenone 5 and carbon dioxide (Scheme 1). These early 20th-century reactions are typical for non-aromatic unsaturated hydrocarbons, and thus provide limited insight into the distinctive reactivity of cumulenes. Subsequent research yielded numerous instances of analogous reactions, which will not be extensively covered in the discussions that follow.^36,37,39–41^

### Cyclooligomerizations

The reactivity of cumulenes is exemplified through their rich oligomerization chemistry. These unique compounds are capable of reacting through various mechanisms, including thermal, photochemical, and catalyzed processes. This section will delve into the array of species formed as a result of oligomerization, illustrating the complexity and versatility of these reactions. Remarkably, oligomerization is postulated as a key mechanism in the highly exothermic, and sometimes explosive, decomposition of carbyne – an sp-hybridized allotrope of carbon.^42^

**Scheme 2:**
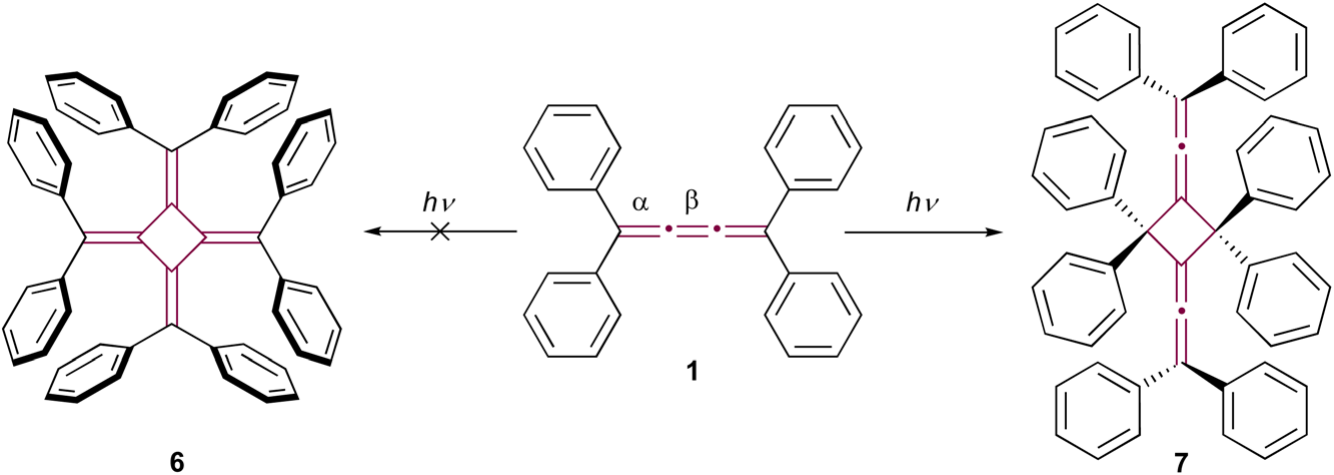
Photodimerization of tetraphenyl[3]cumulene 1. Radialene 6 (left) is initially postulated as the product. The product is later revised and verified by X-ray diffraction analysis as 7 (right).^43,44^

In his pioneering research, Brand noted that the bright yellow color of tetraphenyl[3]cumulene 1 gradually faded when exposed to sunlight, although the structure of the resultant photodegradation product remained unidentified at the time.^8,9^ It wasn't until 1962 that the structure of the product was proposed to be octaphenyl[4]radialene 6 by Uhler *et al.* (Scheme 2).^43^ However, this proposal was later refuted by Leiserowitz and colleagues.^44^ They demonstrated that the photochemical dimerization occurred between the terminal double bonds, α, rather than the central β-bond, leading to the head-to-tail dimer 7 (Scheme 2). In the latter studies, the structural elucidation was supported by single-crystal X-ray diffraction analysis and further bolstered by the structural determination of its ozonolysis products.

**Scheme 3:**
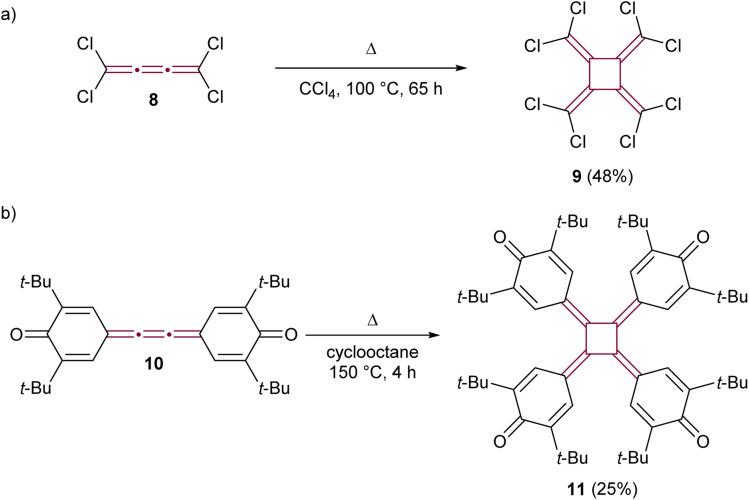
Thermal cyclodimerization of (a) perchloro[3]cumulene 8 and (b) quinone derivative 10.^45–47^

The early reports of cumulene reactivity were followed by the development of further dimerization methodologies. Heinrich and Roedig reported the thermal dimerization of tetrachloro[3]cumulene 8, leading to the formation of perchloro[4]radialene 9 (Scheme 3a).^45^ Koster and West explored the thermal dimerization of extended quinone 10, successfully synthesizing tetraquinocyclobutane 11, a compound notable for its unique redox-active properties and the photochemistry of its dianions (Scheme 3b).^46,47^ While these reactions gave moderate yields, the thermal dimerization conditions they pioneered were subsequently adapted for the synthesis of various [4]radialenes from related [3]cumulenes.^48,49^

**Scheme 4:**
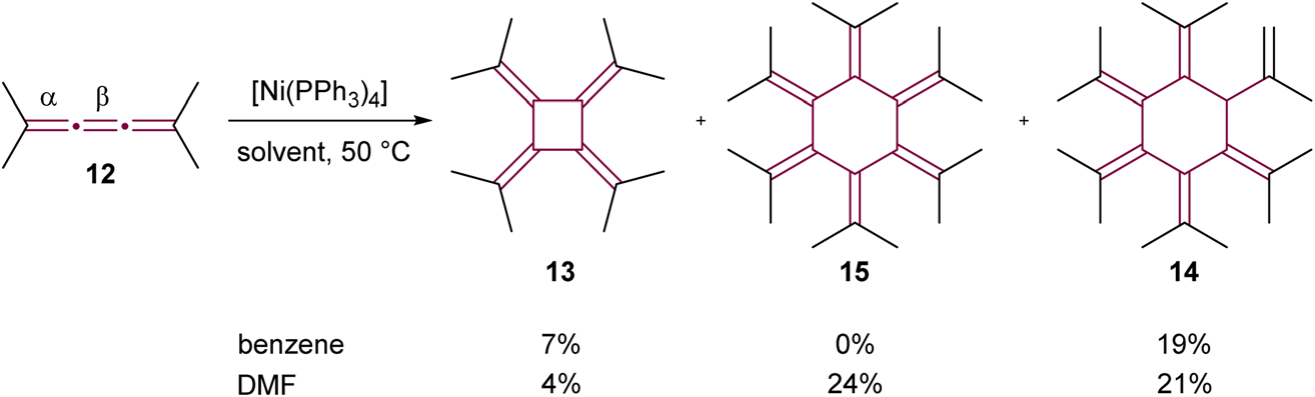
Ni(0)-catalyzed cyclooligomerization of tetramethyl[3]cumulene 12.^51,52^

The utilization of transition-metal complexes as catalysts marked a significant advancement in cumulene dimerization reactions. In initial experiments, d^10^ late transition metal complexes including Pd(0), Pt(0), Rh(i), and Ni(0) were employed to catalyze the dimerization of [3]cumulene 10 into radialene 11 (not shown).^50^ This approach yielded considerable improvements in reaction efficiency over non-catalyzed thermal methods, with the exception of Rh-based catalysts. The complex [Ni(PPh_3_)_2_(CO)_2_] was found to be the most efficient catalyst, delivering 95% isolated yield of radialene after heating for four hours at 80 °C in benzene. Further investigations by Iyoda and coworkers into the cyclooligomerization of tetramethyl[3]cumulene 12, employing *in situ* generated [Ni(PPh_3_)_4_], revealed a solvent-dependent outcome.^51,52^ When performed in benzene at 50 °C, the reaction gave [4]radialene 13 (7%) and cyclic dendralene 14 (19%), while [6]radialene 15 was not observed (Scheme 4). Conversely, the same reaction performed in DMF at 50 °C produced [6]radialene 15 in 24% yield, alongside isomer 14 and [4]radialene 13 in 21% and 4% yields, respectively (Scheme 4).

**Scheme 5:**
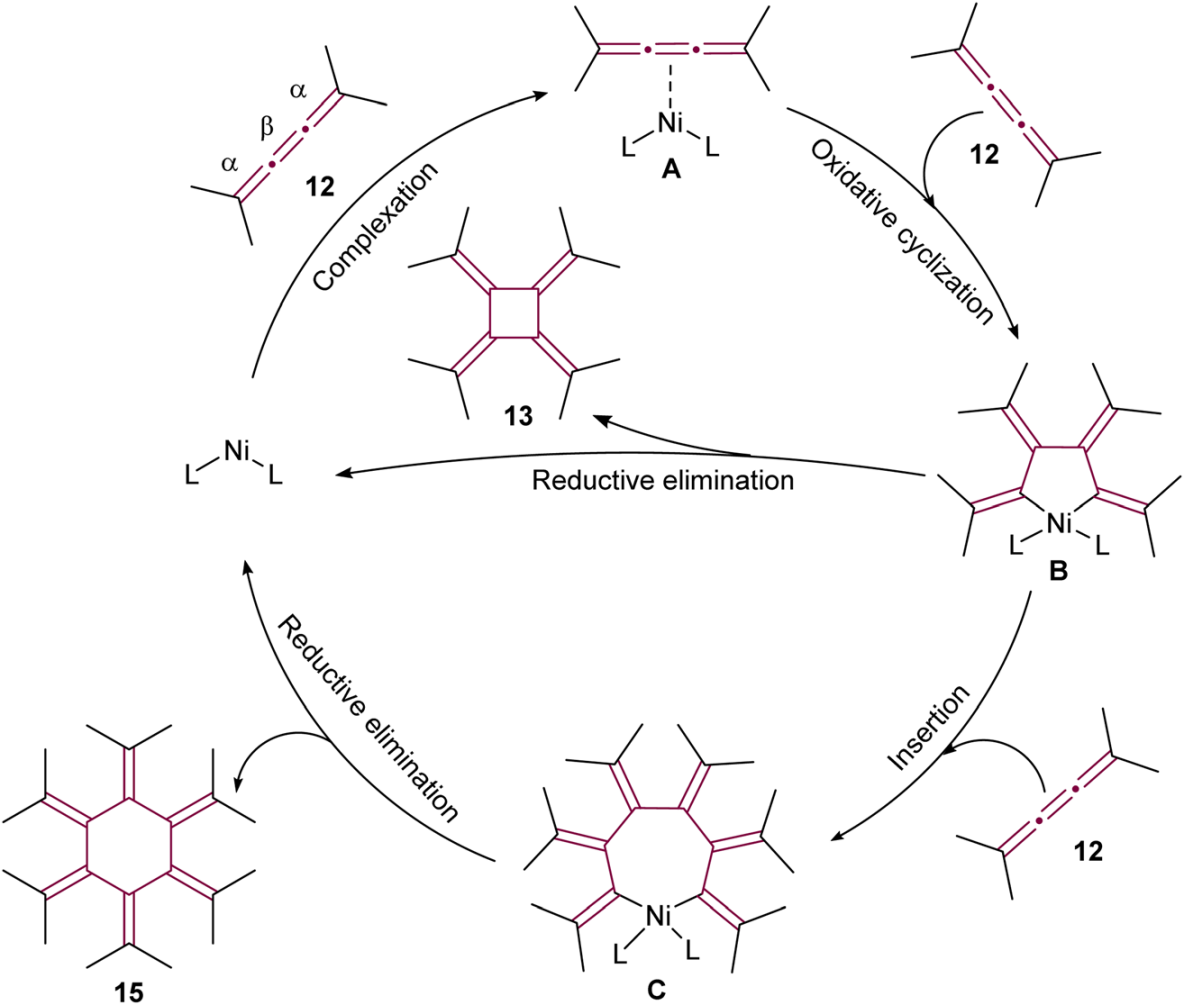
Proposed mechanism of Ni(0)-catalyzed cyclooligomerization of tetramethyl[3]cumulene 12 (L = ligand).^51^

In the proposed reaction mechanism (Scheme 5), initiation of the catalytic cycle occurred through complexation of Ni(0) with the β-bond of 12 to form an *η*^2^–[3]cumulene nickel complex A.^51^ Previously, analogous stable complexes with Rh(i) as the central metal have been reported.^50,53^ The process progressed with the addition of a second molecule of 12, leading to complexation of Ni to the β bond to form a five-membered nickelacycle B. These complexes were isolated and characterized in detail in studies conducted by Wilke and Stehling, employing a more stable bipyridine Ni(0) complex.^54^ At this stage, the insertion of a third molecule of 12 into the metallacycle was possible, resulting in the formation of a seven-membered ring C. The reductive elimination of these metallacycle complexes subsequently yielded [4]- and [6]radialenes 13 and 15, respectively (Scheme 5).

**Scheme 6:**
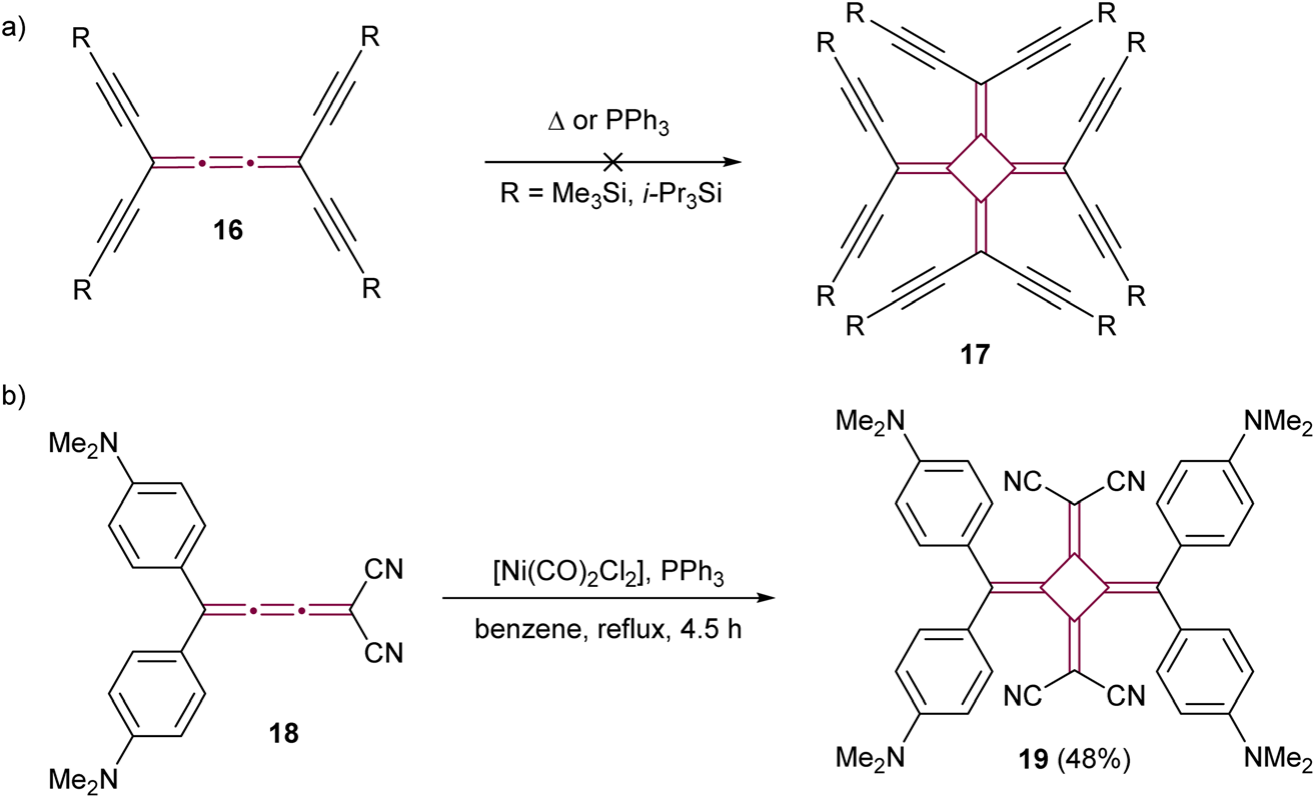
The attempted synthesis of (a) octaethynyl[4]radialene 17 and (b) the successful synthesis of the push–pull [4]radialene 19.^55,56^

The dimerization of cumulenes has proved to be a good strategy for the synthesis of chromophores featuring cross-conjugated carbon structures, specifically [4]radialenes. Diederich and coworkers have explored novel carbon allotropes employing this strategy. Their initial approach involved attempting to dimerize tetraethynyl[3]cumulenes 16 with the aim of synthesizing perethynylated[4]radialenes 17 (Scheme 6a).^55^ Despite their efforts, neither thermal nor the triphenylphosphine-catalyzed method of West^50^ yielded the anticipated product. Rh-catalyzed methods were also investigated, but they solely resulted in the formation of a stable *η*^2^–Rh complex, without yielding the targeted radialene. Later, the same group successfully showed the dimerization of a highly polar push–pull [3]cumulene 18, employing a [Ni(PPh_3_)_2_(CO)_2_] catalyst (Scheme 6b).^56^ The resultant [4]radialene 19 demonstrated intense and broad absorption in the visible spectrum, suggesting its potential suitability for optoelectronic applications.

**Scheme 7:**
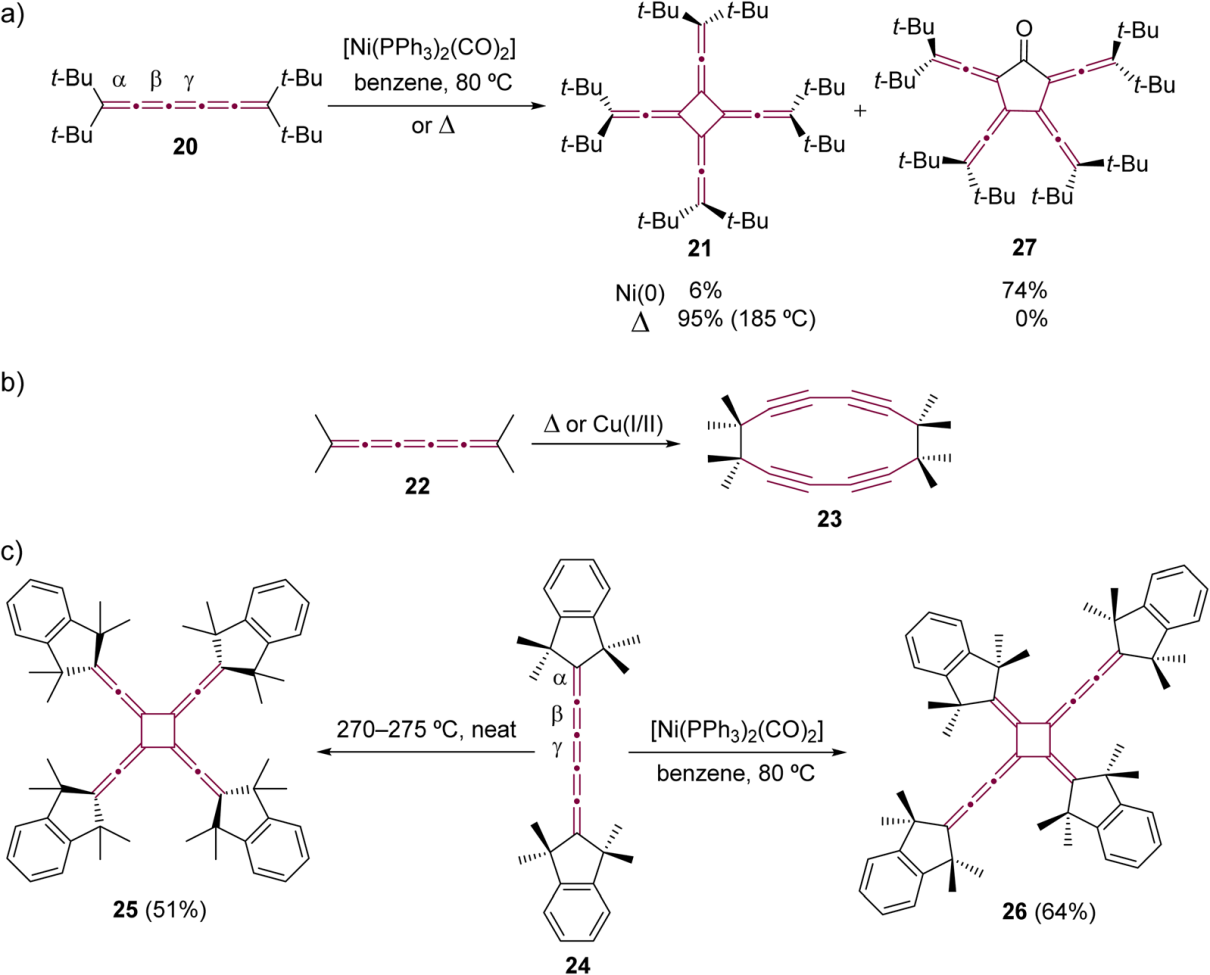
Oligomerizations of [5]cumulenes: (a) tetra(*tert*-butyl)[5]cumulene 20 to extended [4]radialene 21 and cyclic ketone 27,^57,58,69,70^ (b) tetramethyl[5]cumulene 22 to cyclyne 23,^59–61^ and (c) comparison of thermal and Ni-catalyzed dimerization reactions of [5]cumulene 24.^69,71^

The dimerization was extended to include longer [*n*]cumulenes, employing both thermal and Ni-catalyzed techniques. The pioneering work in thermal dimerization of a [5]cumulene was reported by Hartzler in 1966.^57,58^ He observed that tetra(*tert*-butyl)[5]cumulene 20 resolidified after melting at 189 °C forming a white solid, assigned as radialene 21, which formed through dimerization at the central cumulenic double bond (γ) (Scheme 7a). A few years later, Scott and coworkers reported that tetramethyl[5]cumulene 22 undergoes thermal dimerization through the terminal carbon atoms of the cumulene moiety, resulting in the formation of cyclyne 23 (Scheme 7b).^59–61^ A similar reaction was observed during attempts to synthesize unfunctionalized [3]cumulene, which spontaneously dimerized to cycloocta-1,5-diyne (not shown).^35,62–64^ Hopf and Stang proposed, independently, that [5]cumulenes are intermediates in the synthesis of other polyynic macrocycles.^65–67^

The thermal dimerization of [5]cumulenes substituted with sterically demanding alkyl groups consistently yielded *D*_4_-symmetric [4]radialenes, which was later demonstrated in extensive studies by Iyoda and coworkers (Scheme 7c).^68–71^ This research highlights a contrast between the thermal and Ni-catalyzed reactions. The thermal processes predominantly occur at the central γ-bond of the [5]cumulene moiety resulting in *D*_4_-symmetric [4]radialenes, while the Ni-catalyzed reactions tend to involve the β-bond. This distinction is exemplified in a direct comparison using tetramethylindane endcapped cumulene 24, which yields two isomeric radialenes: fourfold-symmetric 25 from neat thermal transformation and head-to-tail dimer 26 from a Ni-catalyzed process.^70^ On the other hand, this trend is not always maintained, and cumulenes decorated with bulky endgroups can yield the same extended [4]radialene *via* either thermal or metal-catalyzed dimerization processes.^72^ For instance, the dimerization of cumulene 20 consistently results in the formation of radialene 21, irrespective of the method employed (Scheme 7a).^57,58,69,70^ This observed regiochemistry could be attributed to the subtly increased steric hindrance induced by the bulky *tert*-butyl substituents, which obstruct access to α- and β-bonds, guiding the reaction toward the γ-bond. Iyoda and coworkers also observed the formation of [5]radialenone 27 as a byproduct in the Ni-catalyzed reaction (Scheme 7a), which arose from the insertion of carbon monoxide, originating from the coordination sphere of nickel, into the intermediate nickelacycle (see Scheme 5).^69,71^

**Scheme 8:**
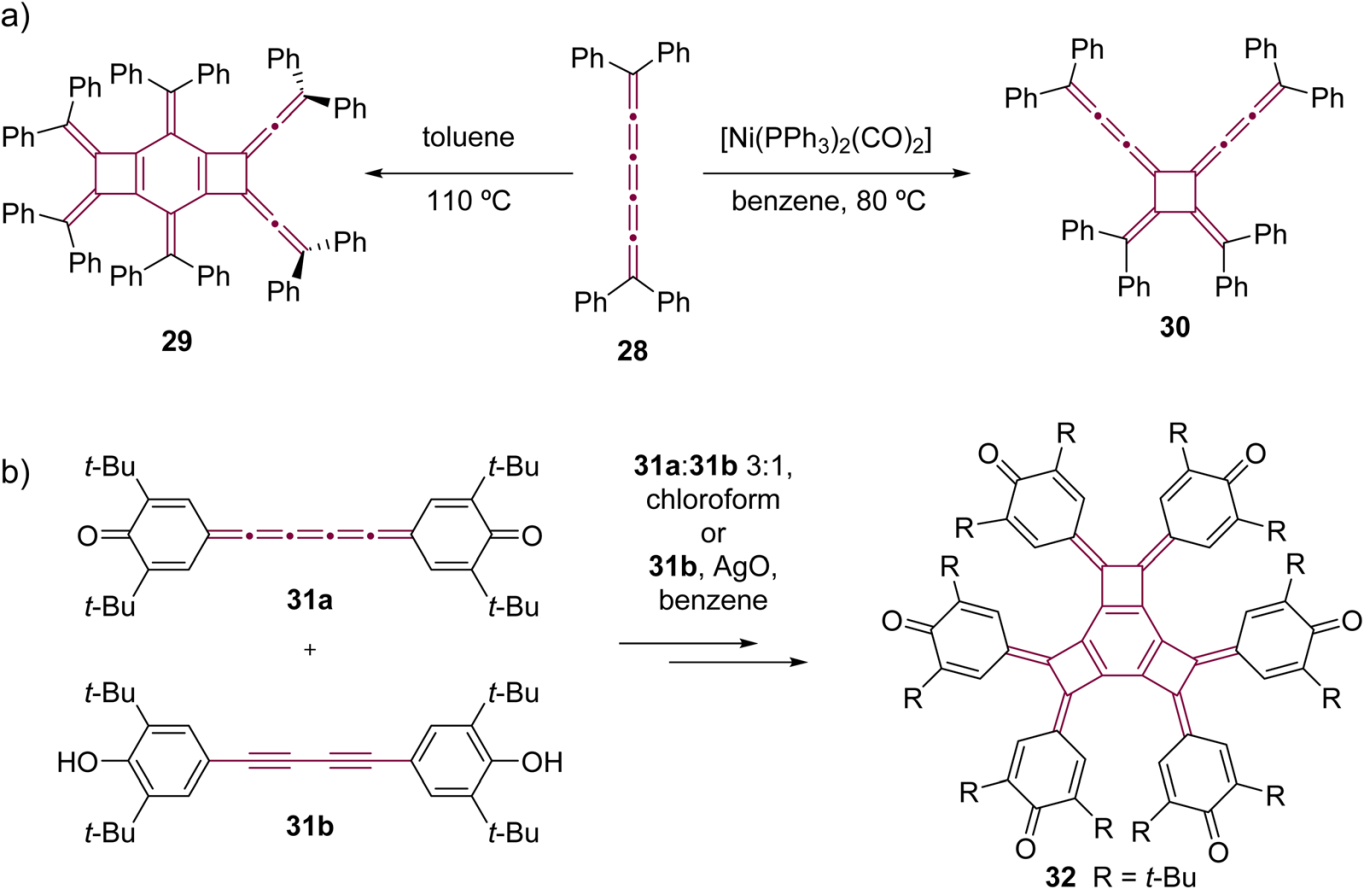
Oligomerizations of [5]cumulenes: (a) thermal trimerization and Ni-catalyzed dimerization reactions of tetraphenyl[5]cumulene 28^68,73^ and (b) trimerization of quinocumulene 31a induced by 31b.^74^

In comparison to [5]cumulenes substituted with alkyl groups, those functionalized with aromatic substituents, such as phenyl groups, exhibit notably different behavior in both thermal and Ni-catalyzed dimerization processes. For instance, when heated in toluene at 110 °C, tetraphenyl[5]cumulene 28 undergoes a tandem trimerization reaction, leading to the formation of the tricyclodecadiene derivative 29, as reported by Kawamura and coworkers (Scheme 8a).^73^ The authors suggested that this reaction initiates with the dimerization of tetraphenyl[5]cumulene 28 into a *D*_4_-symmetric radialene, analogous to the transformation 20 –> 21. This is followed by a [4 + 2] cycloaddition with a third molecule of [5]cumulene 28, and a subsequent electrocyclization culminates in the tricyclic product 29. In contrast, the Ni-catalyzed reaction of 28, using [Ni(PPh_3_)_2_(CO)_2_] in benzene, predominantly yields the *D*_2_-symmetric head-to-head isomer 30. This is a notable deviation from the head-to-tail isomer produced in the reaction with compound 24 (Scheme 7c).^68^ It is not clear if this reactivity difference stems from the steric repulsion of tetramethylindane groups in 24 or the π–π stacking interactions between the phenyl rings in 28, or a combination of both that influence the orientation of cumulenes coordinated at the nickel center during the catalytic cycle. Additional studies reveal that, when catalyzed by Ni(PPh_3_)_4_ instead of [Ni(PPh_3_)_2_(CO)_2_], the dimerization of 28 produces a minor amount of hexa-1,5-dien-3-yne as a by-product (not shown).^68^ These results suggest that the reactivity of tetraphenyl[5]cumulene 28 likely involves competing pathways beyond the straightforward head-to-head dimerization.

Kawase *et al.* observed that quinocumulene 31a undergoes trimerization, forming a tricyclobutabenzene derivative 32 (Scheme 8b).^74^ They suggested a mechanism in which the hydroquinone precursor 31b first oxidizes into a radical intermediate. This radical then initiates dimerization, followed by the addition of [5]cumulene 31a. The reaction sequence concludes with additional oxidation and cyclization, ultimately yielding the tricyclobutabenzene product 32.

**Scheme 9:**
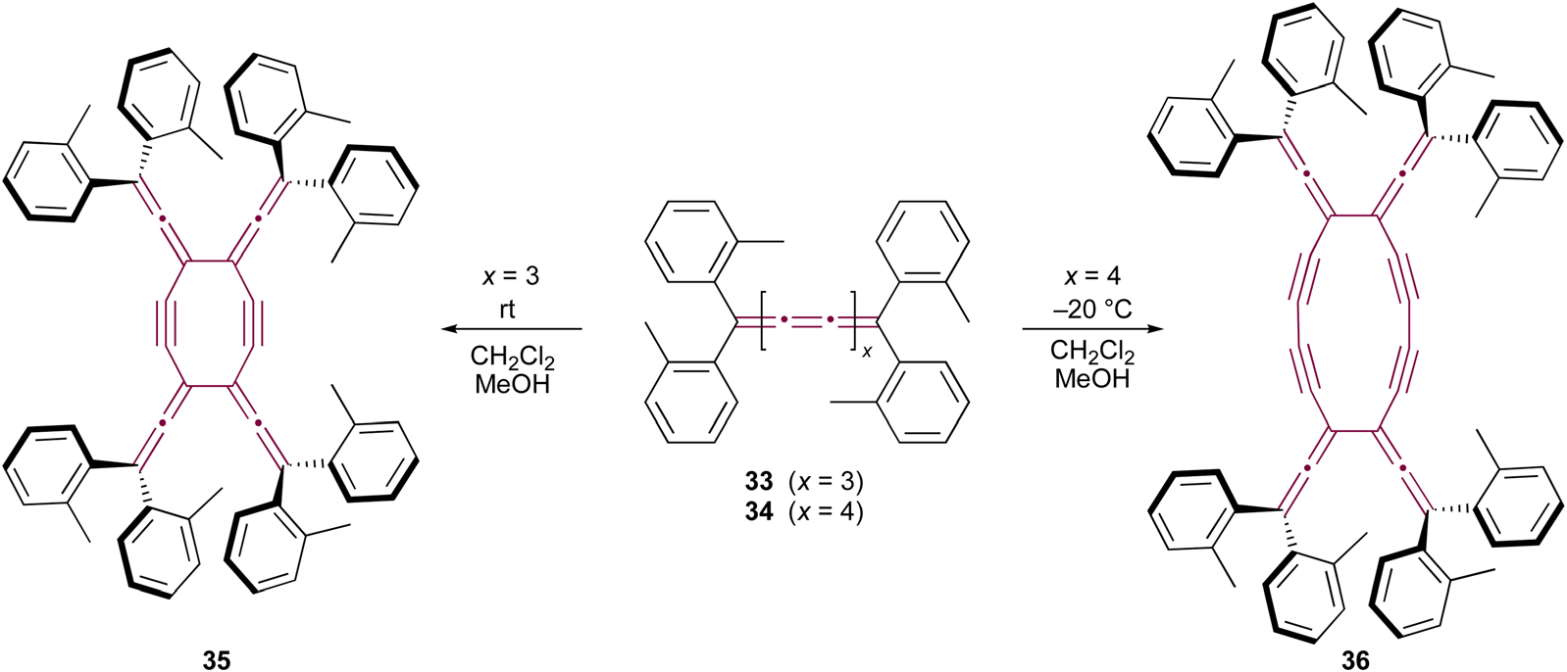
Thermal dimerization of tetraaryl[7]- 33 and [9]cumulenes 34.^75^

Only two examples of dimerization involving [*n*]cumulenes with *n* >5 have been reported, which was described by Wendiger *et al.*, both leading to cross-conjugated expanded radialenes.^75^ The size of the central radialene ring is determined by the length of the cumulene backbone used as the substrate. A consistent structural feature of the resulting products is the presence of diarylvinylidene (allene) units located at the vertices of the expanded radialene framework, indicating preference to react at C3 and C3′ positions. Hence, tetraaryl[7]- and [9]cumulenes (33 and 34, respectively) dimerize to form expanded radialenes 35 and 36 (Scheme 9).

### Cycloaddition reactions

**Scheme 10:**
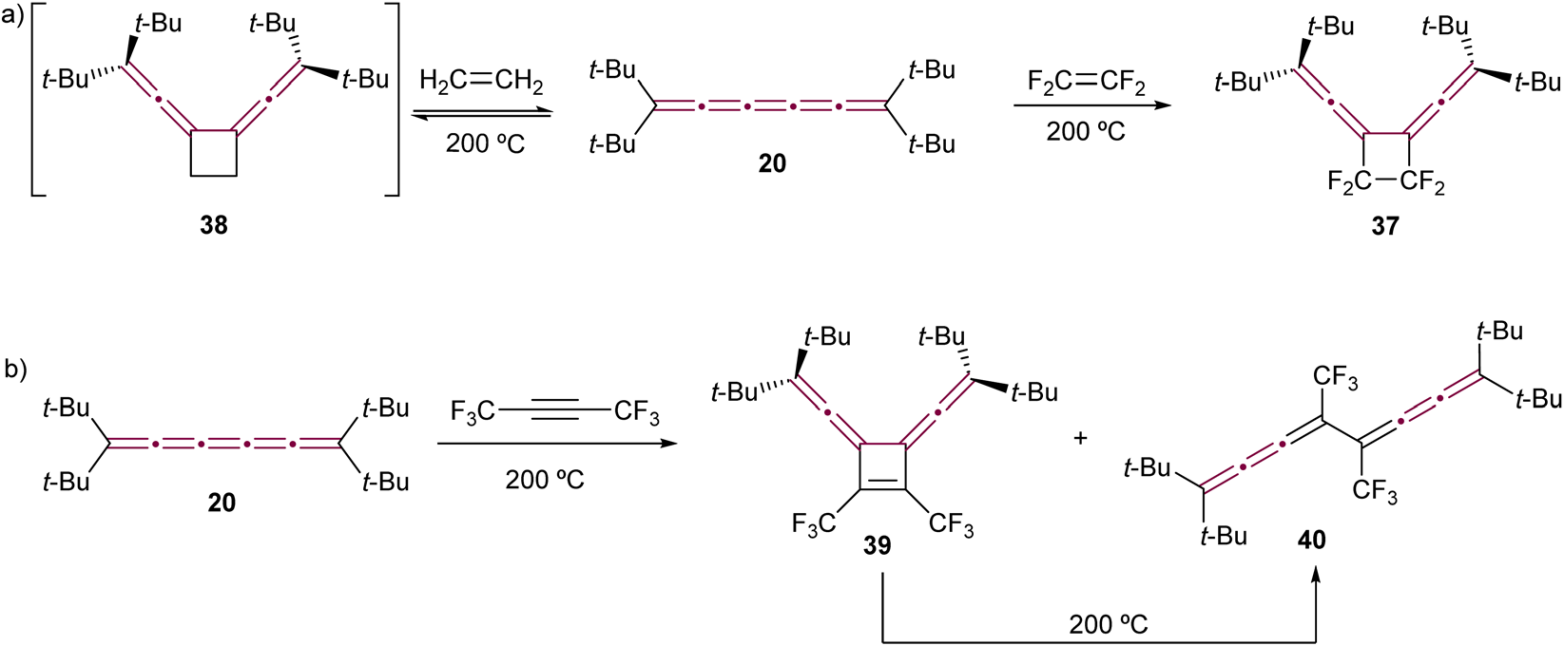
First reported cycloaddition reactions of tetra(*tert*-butyl)[5]cumulene 20 with (a) ethylene (product not isolated) and tetrafluoroethylene and (b) hexafluorobut-2-yne.^57,58^

To the best of our knowledge, the first report of cycloaddition involving a cumulene features tetra(*tert*-butyl)[5]cumulene 20 (the same report that discussed the dimerization of 20 by Hartzler, Scheme 7a).^57,58^ In this study, tetrafluoroethylene reacts with the central γ-bond of cumulene 20 at 200 °C sealed in a Carius tube, forming fluorinated cyclobutene 37 as a result of a formal [2 + 2] cycloaddition reaction (Scheme 10a). Interestingly, the reaction of 20 with ethylene proves to be reversible. The authors postulated the formation of a transient intermediate 38 upon heating with ethylene at 200 °C under pressure, which is found to release ethylene and restore [5]cumulene 20 upon cooling. However, recall that at 200 °C without ethylene, 20 dimerizes to form [4]radialene 21 (Scheme 7a). In the same study, compound 20 is reported to react with hexafluorobut-2-yne at 200 °C, yielding cyclobutene 39 (Scheme 10b). However, under these harsh conditions, 39 isomerizes *via* retroelectrocyclization to form bis[3]cumulene 40.

**Scheme 11:**
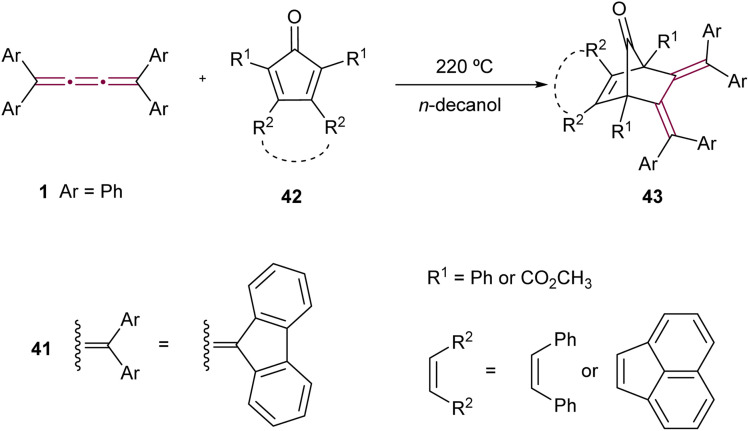
Diels–Alder reactions of tetraaryl[3]cumulenes 1 and 41.^76^

The Diels–Alder reaction, featuring tetraaryl[3]cumulenes as dienophiles, was reported by Ried and Neidhardt.^76^ The reaction of tetraphenyl[3]cumulene 1 and its fluorenylidene endcapped counterpart 41 with various substituted cyclopentadienones 42 produces cycloadducts 43 (Scheme 11). These products are particularly interesting due to their potential conversion, *via* decarbonylation, into *o*-quinodimethanes, compounds that are desirable intermediates in organic synthesis.^77–79^

**Scheme 12:**
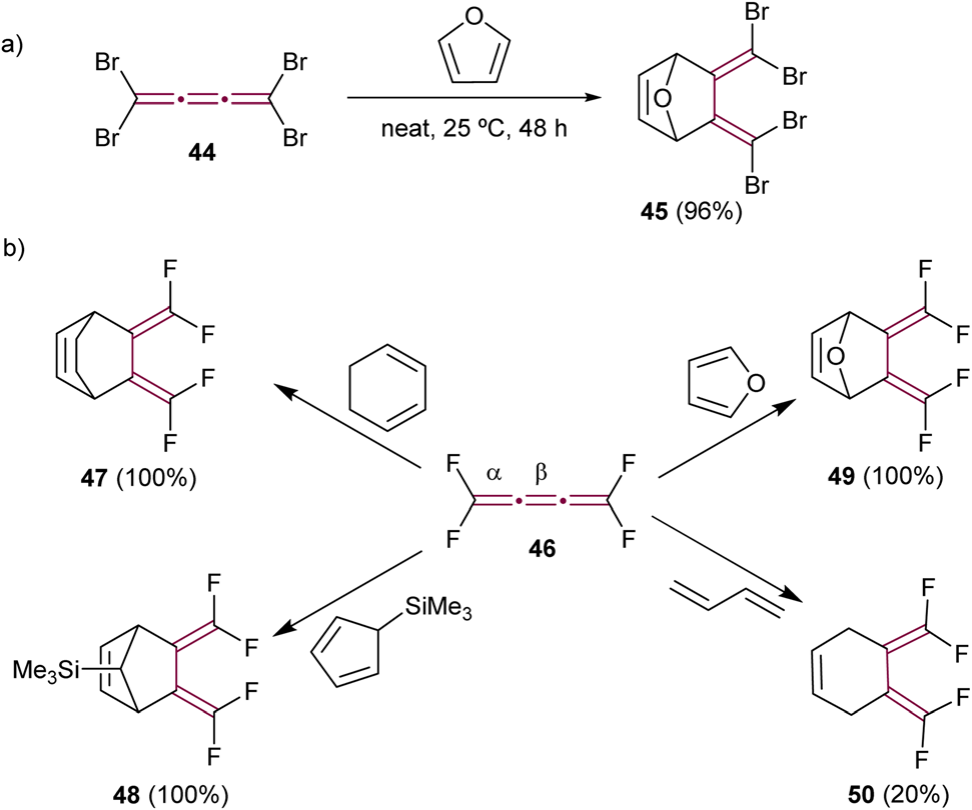
Diels–Alder reactions of (a) tetrabromo- 44 and (b) tetrafluoro[3]cumulenes 46.^80–82^

In their creative approach, Goroff and coworkers utilized the reversible character of the Diels–Alder reaction with furan for the protection of tetrabromo[3]cumulene 44 (Scheme 12a).^80^ This approach served as a preparatory step for subsequent functionalization of the terminal bromine atoms, specifically through Pd-catalyzed cross-coupling reactions that would otherwise engage the cumulene core directly. The reaction was carried out neat in furan and produced adduct 45 in a good yield. In contrast, tetrafluoro[3]cumulene 46, first reported by Martin and Sharkey in 1959, exhibited exceptional reactivity, leading to explosive decomposition in its liquid state.^81^ Consequently, it requires storage at extremely low temperatures, *i.e.*, at −196 °C. Nevertheless, this inherent instability did not prevent Ehm and Lentz from reporting on its reactivity in Diels–Alder reactions. They described the reaction of 46 with various dienes, resulting in stable cycloaddition products (selected examples 47–50 in Scheme 12b). Notably, these reactions involved the dienes reacting selectively with the central β-bond of tetrafluoro[3]cumulene 46 in all cases.^82^

**Scheme 13:**
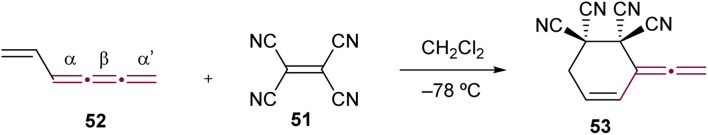
Diels–Alder reaction of 1,2,3,5-hexatetraene 52 with TCNE 51.^96^

Tetracyanoethene (TCNE, 51) shows notable reactivity with electron-rich acetylenes, which, akin to cumulenes, are characterized by linearly aligned sp-hybridized carbon atoms (Fig. 2). The reaction between TCNE and alkynes typically follows a cycloaddition–retroelectrocyclization (CA–RE) reaction cascade.^83,84^ This transformation proves to be an exceptionally efficient and valuable approach for synthesizing push–pull chromophores with low HOMO–LUMO gaps.^30,85–95^ The analogous reactions between cumulenes and TCNE 51 lead to the formation of a diverse array of products, highlighting their versatility as building blocks in organic synthesis. The initial reaction of this type, reported by Hopf and Maurer, is used to confirm the structure of 1,2,3,5-hexatetraene 52,^96^ which represents one of the twelve possible linear isomers of benzene.^97^ In this reaction, cumulene 52 acts as a diene in a Diels–Alder [4 + 2] cycloaddition, leading to the formation of 53 (Scheme 13). This reaction is particularly interesting as it indicates that cycloadditions can involve the terminal α-bond of a [3]cumulene when sterically unencumbered. Supporting this reactivity pattern, a systematic theoretical study by Baroudi *et al.* reveals that both kinetic and thermodynamic factors govern the regioselectivity of cycloadditions in [3]cumulenes.^98^

**Scheme 14:**
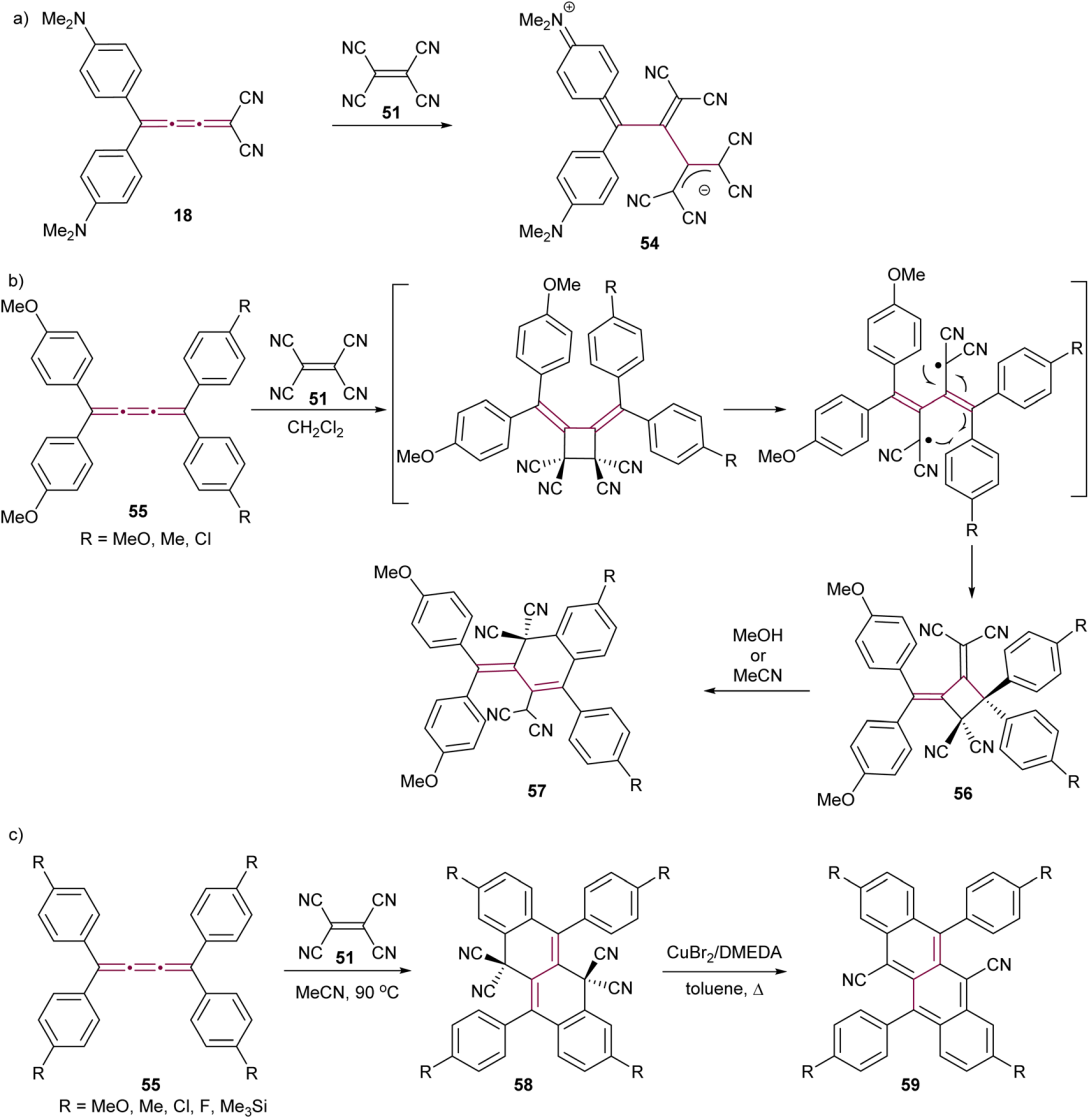
Reaction of TCNE with [3]cumulenes (a) 18, (b) 55, and (c) 55 under different conditions to form tetracenes 59.^100–103^

The Diederich group has demonstrated that a polarized push–pull [3]cumulene 18 exhibits a proacetylenic reactivity engaging in a CA–RE cascade with TCNE to give the stable zwitterion 54 (Scheme 14a).^56^ This reactivity was later extended to a series of push–pull [3]cumulenes by the same research group.^29^ In these studies, the proacetylenic nature of cumulenes was systematically evaluated through X-ray structural characterization and the measurement of rotational barriers about the cumulene axis. Remarkably, the rotational barriers were as low as 12 kcal mol^−1^. These barriers were closer to those associated with sterically hindered single bonds rather than those typical of double bonds, which are expected to be significantly higher at around 65 kcal mol^−1^ for ethylene.^99^ Such results suggest that highly polarized [3]cumulenes bear a greater structural resemblance to the single–triple–single bonding of acetylenes rather than the three contiguous double bonds that are characteristic of cumulenes.

The proacetylenic reactivity of cumulenes with TCNE has been extended to symmetric, non-polar derivatives. Kawamura and coworkers showed that tetraaryl[3]cumulenes 55 undergo a facile reaction with TCNE in CH_2_Cl_2_ at ambient temperature to give cycloadducts 56 (Scheme 14b), which are relatively stable in nonpolar and aprotic solvents.^100^ However, in polar solvents such as MeOH or MeCN, compounds 56 undergo an ionic transformation, leading to hydronaphthalenes 57 through ring opening followed by intramolecular cyclization of one of the aromatic substituents (Scheme 14b).^101^ Independently, Gawel *el al.* have detailed that the reaction of TCNE with tetraaryl[3]cumulenes 55 does not stop at 57 when performed in acetonitrile at elevated temperatures, but gives instead dihydrotetracenes 58 as the products of a multistep domino reaction (Scheme 14c).^102^ Detailed mechanistic investigations reveal that, after an initial CA–RE reaction between TCNE and the central β-bond of the [3]cumulene, two sequential 6-electron electrocyclizations furnish the tetracyclic carbon scaffold, which is followed by a 1,5-hydrogen shift and H_2_ elimination to give 58. In the presence of metallic copper, decyanation and aromatization are achieved by heating 58 neat, leading to cyanotetracenes 59. Subsequently, a solution-based method has been developed using Cu(i)-*N,N′*-dimethylethylenediamine complex as a catalyst.^103^ The resulting dicyanotetracenes 59 are strong fluorophores and undergo efficient singlet fission in the solid state.^104,105^

**Scheme 15:**
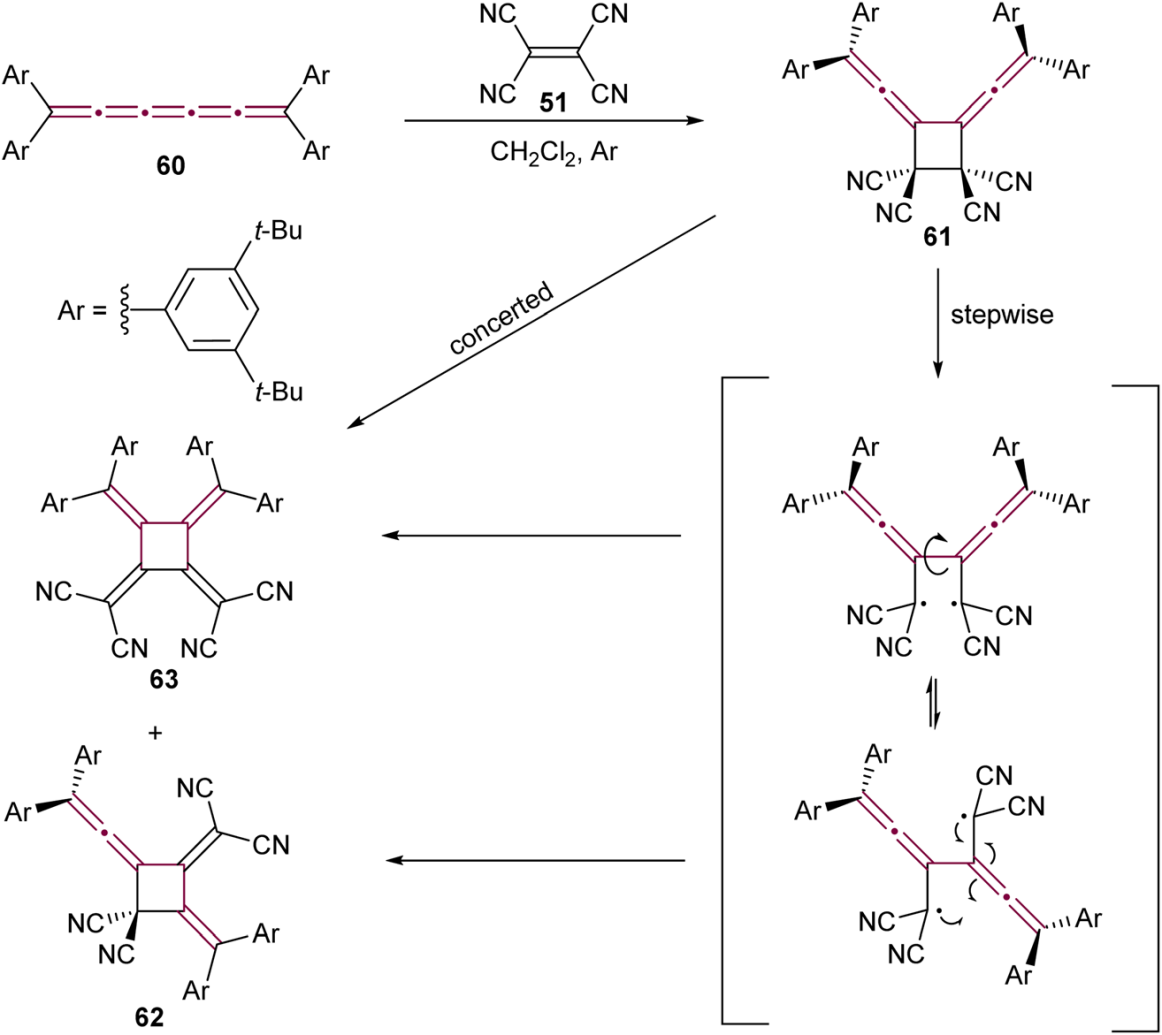
Reaction of [5]cumulene 60 with TCNE.^106^

Expanding on the reactivity of [*n*]cumulenes with TCNE, Januszewski *et al.* reported a complex outcome from the reaction between TCNE 51 and tetraaryl[5]cumulene 60 (Scheme 15).^106^ The initial cycloaddition product, a vinylidene cyclobutane 61, is isolable only at low temperatures. At room temperature, it undergoes a sequence of ring opening and closure, to form the cyclic [3]dendralene 62 and the electron-deficient [4]radialene 63.

**Scheme 16:**
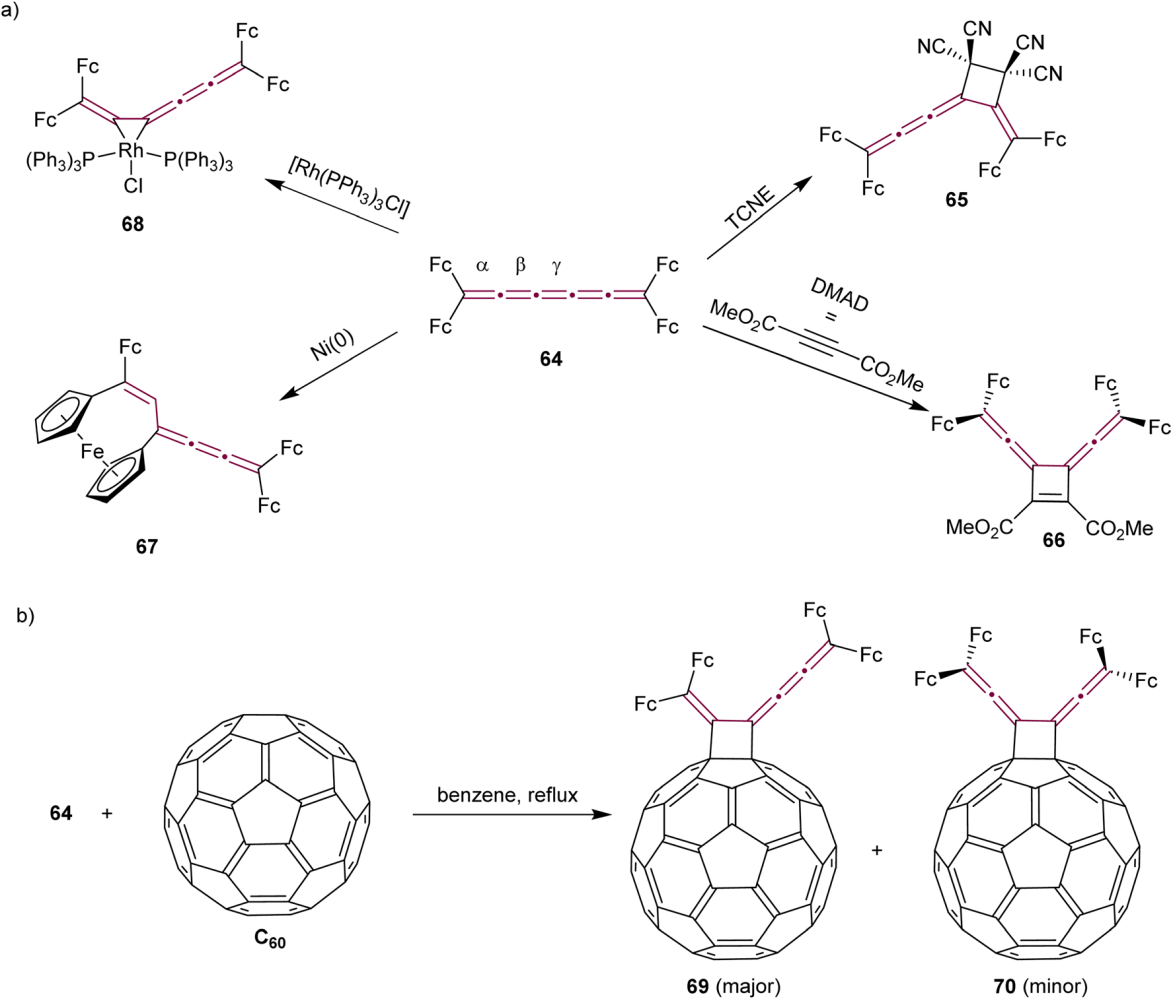
Reactivity of tetraferrocenyl[5]cumulene 64 (Fc = ferrocenyl) (a) with metals and in cycloaddition reactions and (b) in the cycloaddition reaction with C_60_.^107^

Tetraferrocenyl[5]cumulene 64, studied by Bildstein and coworkers, reacts with TCNE *via* a formal [2 + 2] cycloaddition at the β-bond of the [5]cumulene to form the stable cyclobutane 65 (Scheme 16a).^107^ The reaction of 64 with the electron-poor alkyne DMAD, however, takes place at the central γ-bond to produce cycloadduct 66 with a characteristic dark blue color. Attempts at [2 + 2] cyclodimerization with Ni(0) to produce octaferrocenyl[4]radialenes lead to an unexpected outcome and the formation of a unique [3]ferrocenophane 67. In the realm of coordination chemistry, 64 forms a stable complex with [Rh(PPh_3_)_3_Cl]. The [2 + 2] cycloaddition of cumulene 64 with C_60_ yields regioisomers 69 and 70. The isomer 70 with *D*_2*h*_ symmetry, is the minor product that could not be isolated, whereas the green, air-stable isomer 69 is the major product, isolated in 68% yield (Scheme 16b).

**Scheme 17:**
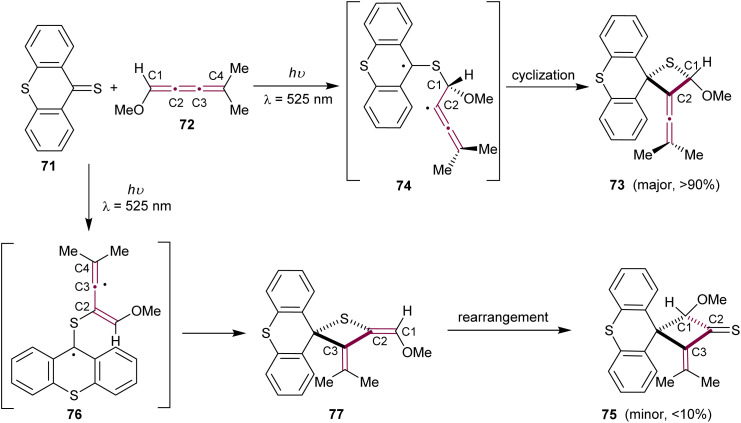
Photochemical [2 + 2] cycloaddition of thioxanthenethione 71 to [3]cumulene 72.^108^

In 1979, Bos and coworkers reported the heteroatomic photochemical [2 + 2] cycloaddition reaction between thioxanthenethione 71 and [3]cumulene 72, which leads to the formation of cyclic thioether 73 as the major product (Scheme 17).^108^ This report is a natural expansion of their earlier research on photochemical additions of thiocarbonyl compounds to acetylenes, allenes, and ketenimines *via* [2 + 2] and [4 + 2] cycloadditions.^109^ In the reaction pathway, thioxanthenethione 71 in its triplet state preferentially attacks cumulene 72 at the terminal sp^2^-carbon, resulting in a stabilized biradical 74. Subsequent ring closure produces the thietane derivative 73. Interestingly, the formation of a minor isomeric byproduct, cyclobutanethione derivative 75, can be explained by the thioxanthenethione 71 attack at C2 of cumulene 72, followed by ring closure of the biradical 76 to give thione 77. The thione 77 then undergoes a rearrangement, resulting in the formation of the final cyclobutanethione 75 (Scheme 17).

**Scheme 18:**
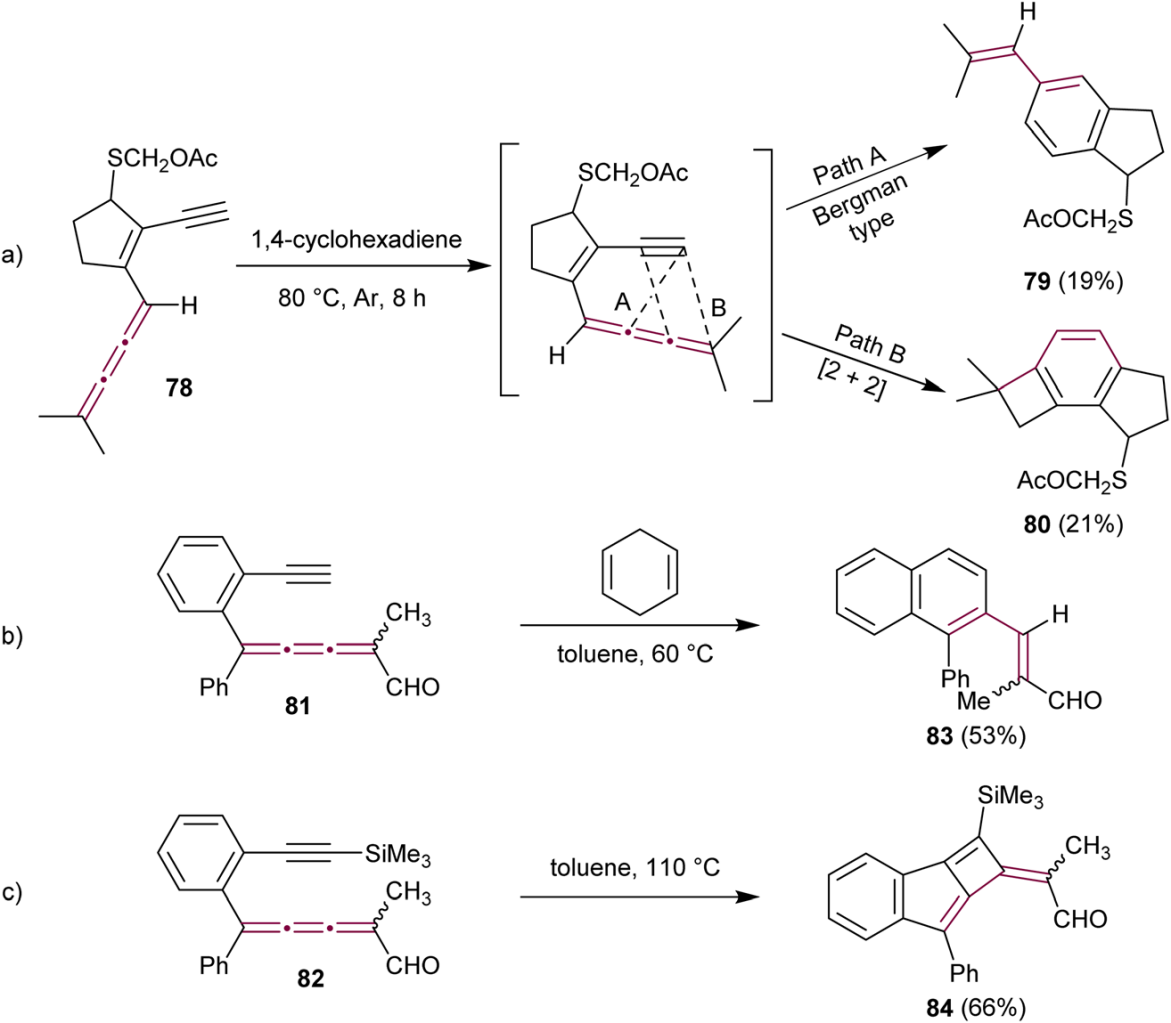
Intramolecular cyclization reactions of enyne[3]cumulenes: (a) 78*via* a Bergman-type cyclization (Path A) or [2 + 2] cycloaddition (Path B), (b) Bergman-type cyclization reaction of 81, and (c) [2 + 2]cycloaddition reaction of 82.^113,114^

In the context of research dedicated to the mechanism underlying the antitumor efficacy of the endiyne antibiotic neocarzinostatin, a series of studies have examined the intramolecular cyclization of enyne[3]cumulenes.^110–112^ The Hirama group reported on the intramolecular electrocyclic reactions of enyne[3]cumulene 78.^113^ Under thermal conditions (in deoxygenated 1,4-cyclohexadiene at 80 °C), 78 gave a mixture of styrene 79 (19%) and benzocyclobutane 80 (21%) as products of Bergman-type cyclization and [2 + 2] cycloaddition pathways, respectively (Scheme 18a). With a meticulous mechanistic investigation, this study shows that the reactions indeed follow first order kinetics that are characteristic of intramolecular reactions and that radical species are involved.

A few years later, Rodríguez and coworkers have explored the intramolecular cycloadditions in enyne[3]cumulenals 81 and 82.^114^ In these cases, the reaction pathway depends on the terminal substitution of the alkyne (Schemes 18b and c). [3]Cumulene 81, bearing a terminal alkyne, converts efficiently into naphthalene 83*via* a Bergman-type cyclization, whereas a trimethylsilyl-protected alkyne 82 gives cyclobutene 84 as a result of an intramolecular [2 + 2] cycloaddition reaction (Schemes 18b and c). Both computational investigations and experimental observations suggest that steric effects play a crucial role in the intramolecular cyclization of enyne[3]cumulenes, with bulky substituents promoting [2 + 2] cycloaddition over Bergman-type cyclization.

**Scheme 19:**
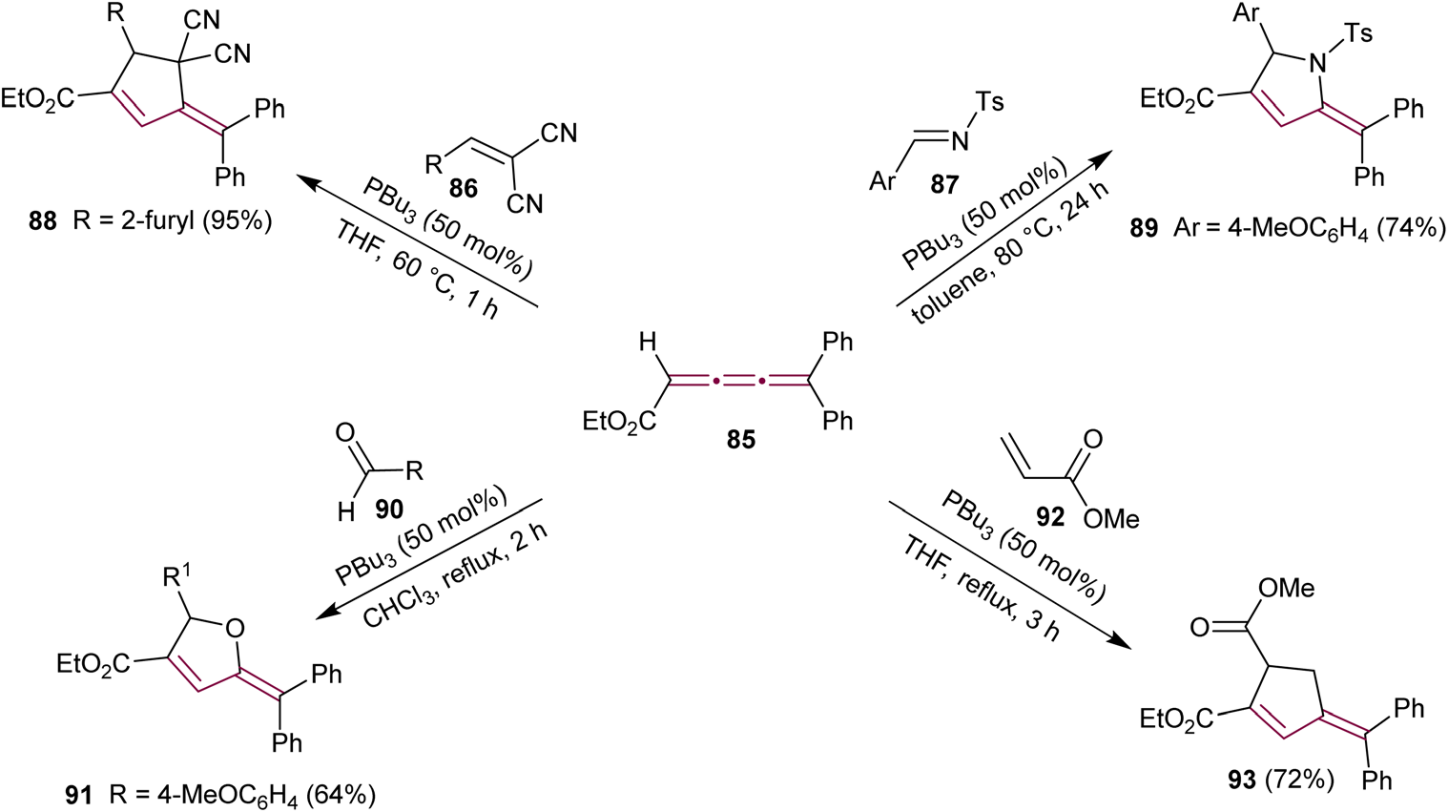
Phosphine-mediated [3 + 2] cycloaddition reactions of ethyl 5,5-diarylpenta-2,3,4-trienoates 85.^115,117^

The potential of cumulenes as synthons was further demonstrated by Guan and Shi, who reported the phosphine-mediated [3 + 2] cycloaddition reactions of [3]cumulene 85 (Scheme 19).^115^ In the presence of PBu_3_, [3]cumulene 85 reacts with substituted methylidenemalononitrile 86 or *N*-tosylimine 87 to give substituted cyclopentene 88 and pyrrolidine 89, respectively. A plausible reaction mechanism was proposed based on previously reported phosphine-catalyzed [3 + 2] cycloadditions.^116^ The first step involves the nucleophilic attack of PBu_3_ on 85 to produce a zwitterionic intermediate that attacks the electrophile 86 (or 87), followed by an intramolecular conjugate addition and elimination of phosphine.

Another report showed that the tributylphosphine-catalyzed [3 + 2] cycloaddition of 85 with the aromatic aldehyde 90 leads to the formation of substituted furan derivative 91.^117^ Additionally, the reaction of 85 with the α,β-unsaturated carbonyl esters, such as 92, yields the substituted cyclopentene 93 (Scheme 19).

**Scheme 20:**
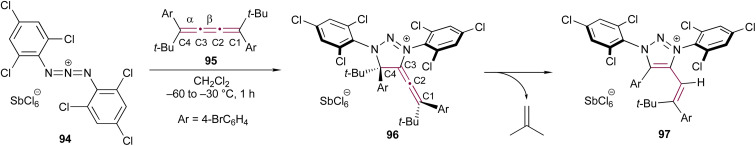
Reaction of [3]cumulene 95 with 1,3-diaza-2-azoniaallene salt 94.^118^

In an effort to explore the reactivity of 1,3-diaza-2-azoniaallene salt 94, Jochims and coworkers examined the reaction with [3]cumulene 95, leading to the formation of allene 96 (Scheme 20).^118^ The cycloaddition occurs exclusively at the α-bond of the [3]cumulene moiety. Computational studies indicate that 96 is 3–11 kcal mol^−1^ more stable than any alternative regioisomer that could be formed upon addition to the central β-bond of 95. Interestingly, upon recrystallization, 96 transforms into the triazolium salt 97 with the loss of isobutene. According to the proposed mechanism, this transformation proceeds *via* elimination of Me_3_C^+^ from the 4-position of 96, followed by proton transfer from Me_3_C^+^ to the intermediate structure, ultimately yielding isoprene and 97.

### Organometallic reactions

Among the many tools available in modern organic synthesis, organometallic reactions stand out for their versatility in constructing complex molecular architectures.^119^ Within this context, cumulenes are intriguing, yet underexplored, substrates, and their distinct bonding pattern and electronic structure impart unique reactivity patterns that hold promise for organometallic transformations. Due to their accessible π-systems and their suitable energy levels, cumulenes react readily with many metals, ranging from alkali to late transition metals. Among many unsaturated hydrocarbons, [*n*]cumulenes have been widely used as ligands in coordination chemistry;^53,120–141^ however, here, we focus mainly on the utility of cumulenes as synthons in organic synthesis. Hence, in this section, we explore the nuanced reactivity of cumulenes mediated by metal centers.

**Scheme 21:**
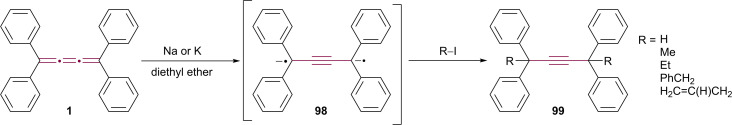
Formation and reactivity of anionic species derived from tetraphenyl[3]cumulene 1.^142^

The work of Zweig and Hoffmann marked the first reported metal-induced reaction involving a cumulene, detailing the reduction of tetraphenyl[3]cumulene 1 using metallic sodium or potassium in an ethereal solution (Scheme 21).^142^ This reaction initially forms a radical anion intermediate, which subsequently undergoes reduction to yield dianion 98. The dianion appears as a transient brown suspension that gradually fades over time. Electron spin resonance (ESR) results reveal a weak signal corresponding to the radical intermediate, which diminishes and disappears within two to three hours. The disappearance of the ESR signal suggests conversion to ESR-silent dianion 98 or other closed-shell species. Treatment of dianion 98 with alkyl halides leads to functionalization at propargylic positions affording 99 (Scheme 21). This study was followed by the study of Day and coworkers who explored the scope of alkali metal-induced reactions of 1 and other tetraaryl[3]cumulenes.^143,144^

Dianions of tetraphenyl[3]cumulene 1 have been extensively studied by Kornacki and Kemula using polarographic methods as well as structurally characterized by X-ray crystallography by Bock and coworkers.^145–148^ Recently, in their collaborative efforts, the groups of Tykwinski and Petrukhina isolated and characterized complexes of [3]- and [4]cumulenes with alkali metals, showing structural changes in cumulenic cores upon reduction.^149,150^

**Scheme 22:**
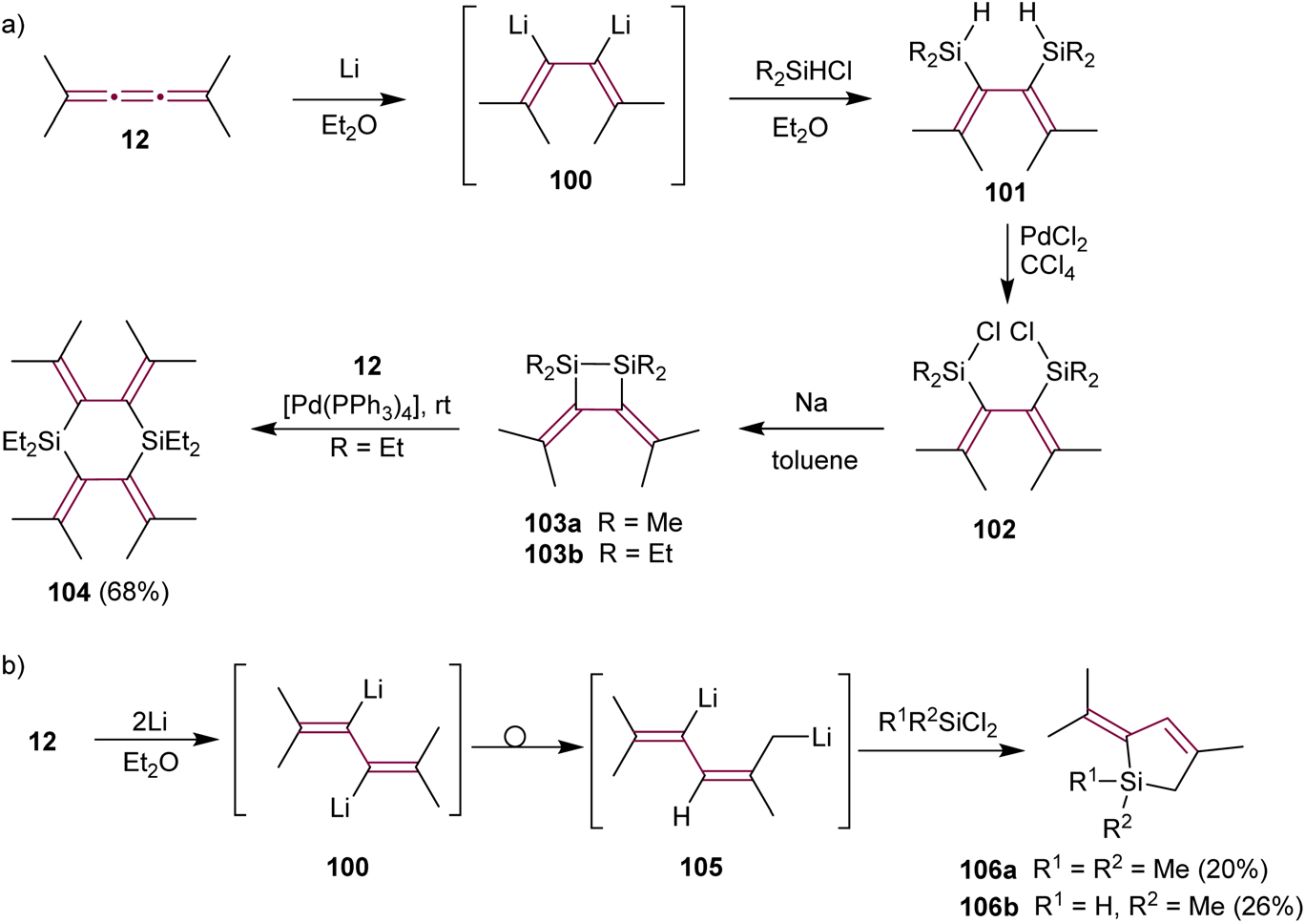
Lithium-induced reactions of tetramethyl[3]cumulene 12 with (a) chlorosilanes and (b) dichlorosilanes.^151,153^

Lithiation of the alkyl-substituted tetramethyl[3]cumulene 12 shows different selectivity than tetraphenyl derivative 1. Ando and coworkers postulated that treating 12 with excess Li metal in Et_2_O produces organometallic intermediate 100 (Scheme 22a). Trapping this dianion with a chlorosilane (R_2_SiHCl) affords the bis-silylated diene 101*via* Li/Si exchange. The subsequent reaction of 101 with PdCl_2_ yields chlorodisilanes 102. Dienes 102, upon reduction with sodium, give disilacyclobutanes 103a or 103b.^151^ Such disilanes are useful reagents in transition metal-catalyzed double silylation of C–C multiple bonds.^152^ Interestingly, the nature of alkyl substituents of the silane affects the yields of doubly silylated products and subsequent disilane metathesis. While methylsilane derivative 103a displays high reactivity and requires storage in dilute solution to prevent polymerization, ethyl-functionalized 103b exhibits remarkable stability for several months even in neat form and at room temperature. Ethylsilane 103b reacts with [3]cumulene 12 in a Pd(0)-catalyzed reaction producing cyclic silane 104 (Scheme 22a). Subsequently, Maercker and coworkers report that intermediate 100 rearranges to the 2,5-dilithiated isomer 105, which is then transformed to dihydrosilols 106 (Scheme 22b).^153^

**Scheme 23:**
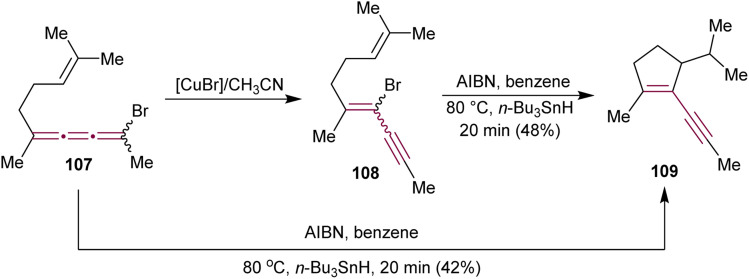
Cyclization of bromo[3]cumulene 107 and bromoenyne 108 to cyclopentene 109.^154^

In 1990, Ziegler have detailed a study on the Cu-mediated isomerization of bromo[3]cumulene 107 to bromoenyne 108, followed by vinyl radical cyclization that leads to the synthesis of substituted cyclopentene 109 (Scheme 23).^154^ This enyne moiety is a key component of the strained fused ring system found in the antitumor antibiotic neocarzinostatin.^110–112^ Interestingly, treatment of 107 with AIBN and *n*-Bu_3_SnH leads directly to 109*via* 5-(π-endo)-*exo*-trig cyclization. Both 107 and 108 convert to 109*via* a common planar radical intermediate.

**Scheme 24:**
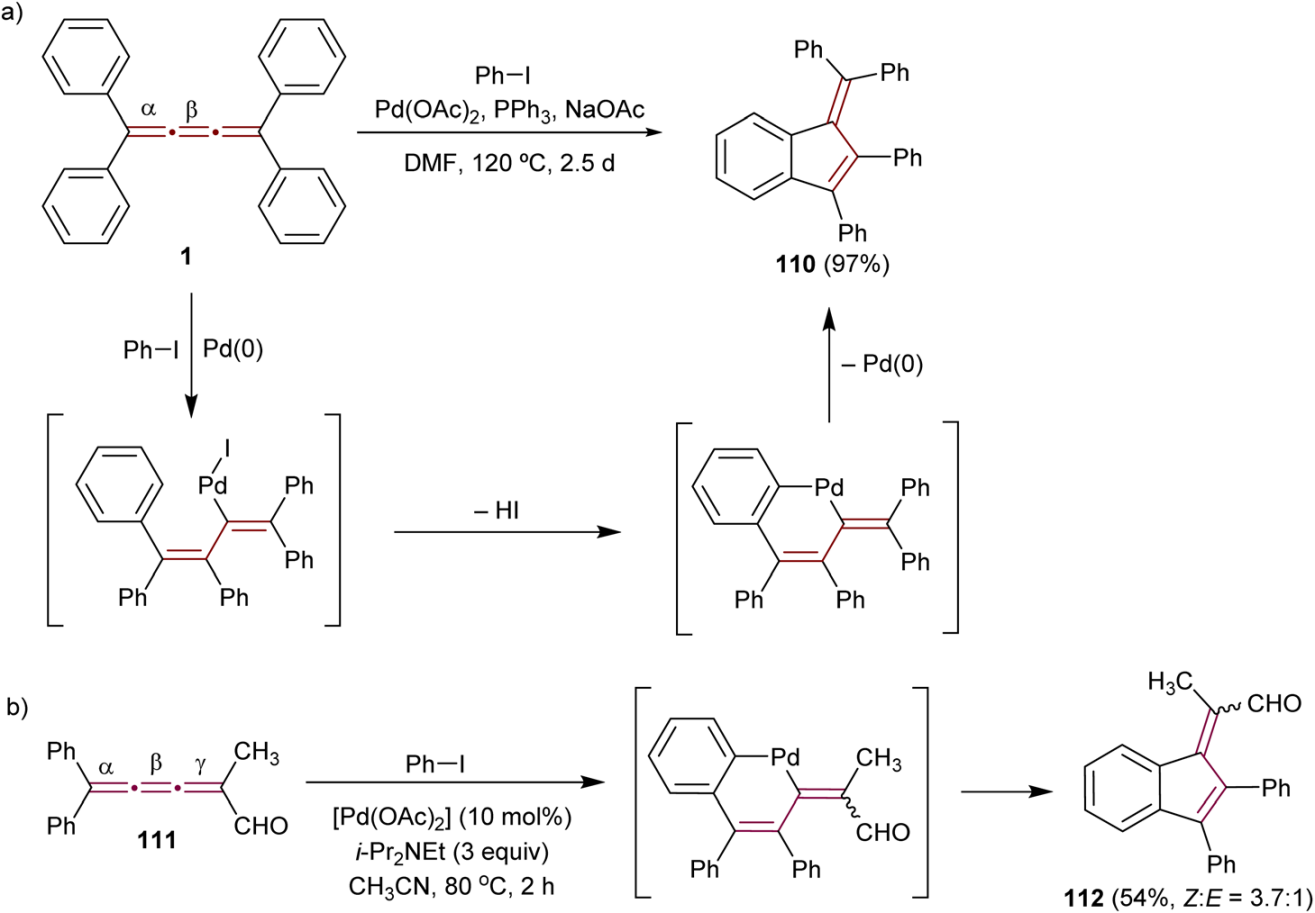
Carbopalladation reactions of [3]cumulenes (a) 1 and (b) 111.^155–157^

The pioneering Pd-catalyzed arylation of cumulenes was demonstrated by Jones and coworkers. They demonstrated that [3]cumulene 1 reacts with iodobenzene through a Pd-mediated catalytic cycle to give substituted benzofulvene 110 in 97% yield (Scheme 24a).^155^ This transformation is proposed to commence with a carbopalladation across the β-bond of the cumulene moiety and then proceed through a cyclopalladation/C–H activation step. Reductive elimination to 110 concludes the final step. Kan and coworkers applied a similar Pd-catalyzed reaction to convert [3]cumulenal 111 into benzofulvene 112 (Scheme 24b). The scope of this reaction is broadened to provide good yields using a range of aryl halides containing either electron-donating or -withdrawing groups.^156,157^ Remarkably, the conjugated carbonyl group in 111 does not alter the course of the reaction: the aryl-palladium intermediate still inserts regioselectively into the β-double bond. Subsequent C–H activation with a neighboring phenyl group leads to the formation of 2,3-benzofulvene derivatives 112 with a substituted exocyclic alkene. This domino process deviates from allene formation initially anticipated by the authors. Aryl iodides containing both electron-donating and -withdrawing groups produce corresponding products in good yields.

**Scheme 25:**
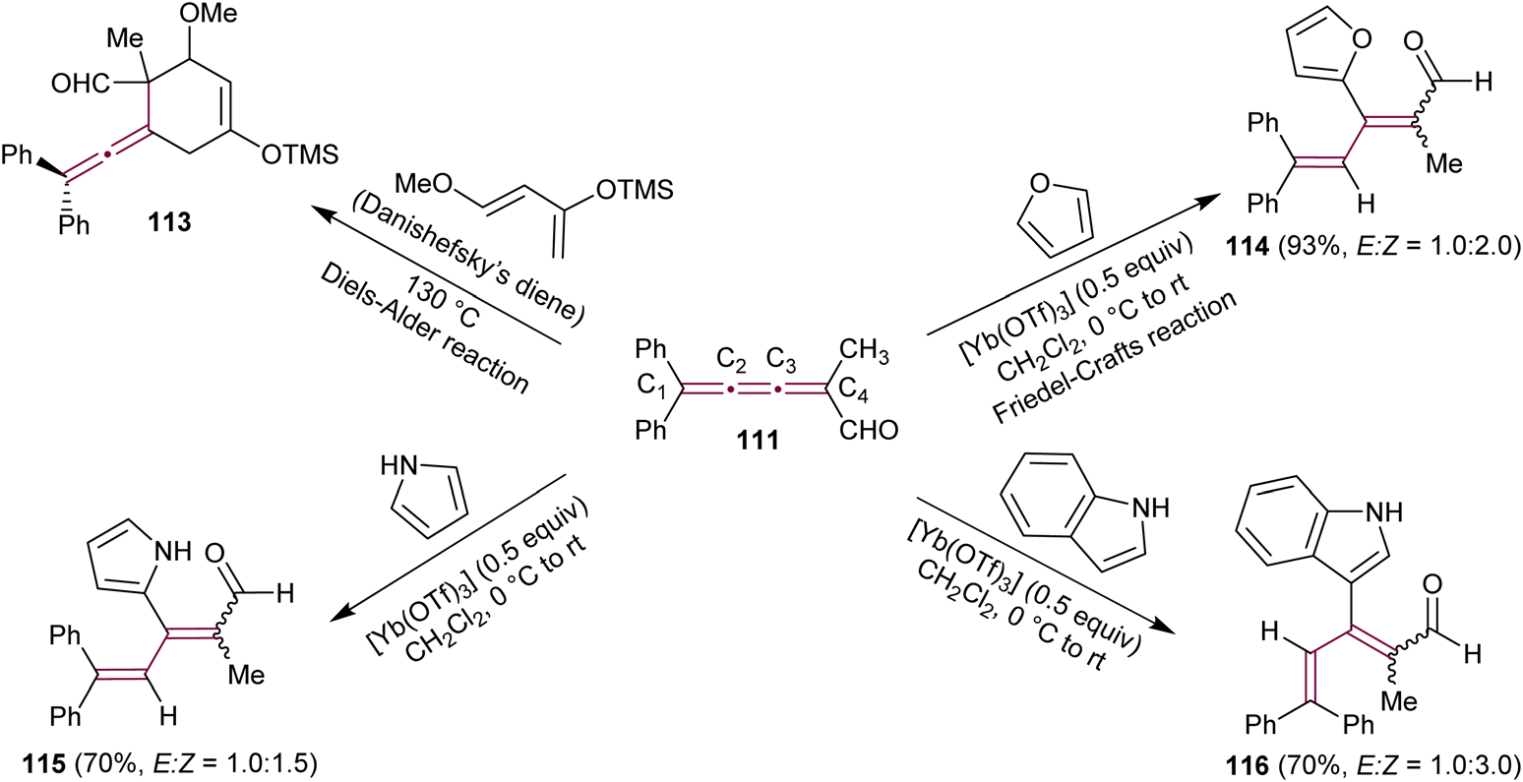
Reactions of cumulenal 111 in Diels–Alder and Friedel–Crafts reactions.^158^

Kan's group expanded the use of [3]cumulenal 111 as a synthon (Scheme 25).^158^ In a Lewis-acid catalyzed Diels–Alder reaction, cumulene 111 reacts efficiently with Danishefsky's diene producing an unstable allenyl cycloadduct 113. The reaction of 111 with cyclopentadiene is also described in detail (not shown). Notably, Diels–Alder reactions occur selectively at the C3–C4 double bond. Further investigations revealed the successful Friedel–Crafts reactions with furan, catalyzed by the Lewis acid Yb(OTf)_3_, yielding tetrasubstituted, conjugated diene 114 in a 93% yield. This reaction was proposed to proceed through the conjugate addition at C3 of the cumulene, followed by subsequent protonation at C2. The Friedel–Crafts reactions with nitrogen-containing heteroaromatics, such as pyrrole and indole, were also reported. In all cases, the reactions proceed smoothly, yielding the corresponding dienes 115 and 116 in excellent yields, with no evidence of the Diels–Alder product or the corresponding allene derivative. To validate the reactivity of 111, the authors also tested the reaction with other nucleophiles, such as thiolates. Similar to the Friedel–Crafts reaction, the conjugate addition of the thiolate anion occurs at C3, followed by protonation at C2, resulting in the conjugated diene with same regioselectivity as 114 and 115.^158^ The conjugate addition–protonation sequence can be considered as a formal 3,4-addition, forming tetrasubstituted conjugated dienes. This reactivity stands in stark contrast to the Diels–Alder reaction, which occurs at the C3–C4 bond of 111, leading to tetrasubstituted allene derivatives, such as 113. As expected, this reactivity is influenced by electronic and steric factors inherent for each double bond. The electron-withdrawing effect of the formyl group and the steric hindrance of the diphenyl moiety are postulated as key factors contributing to this unique reactivity.

**Scheme 26:**
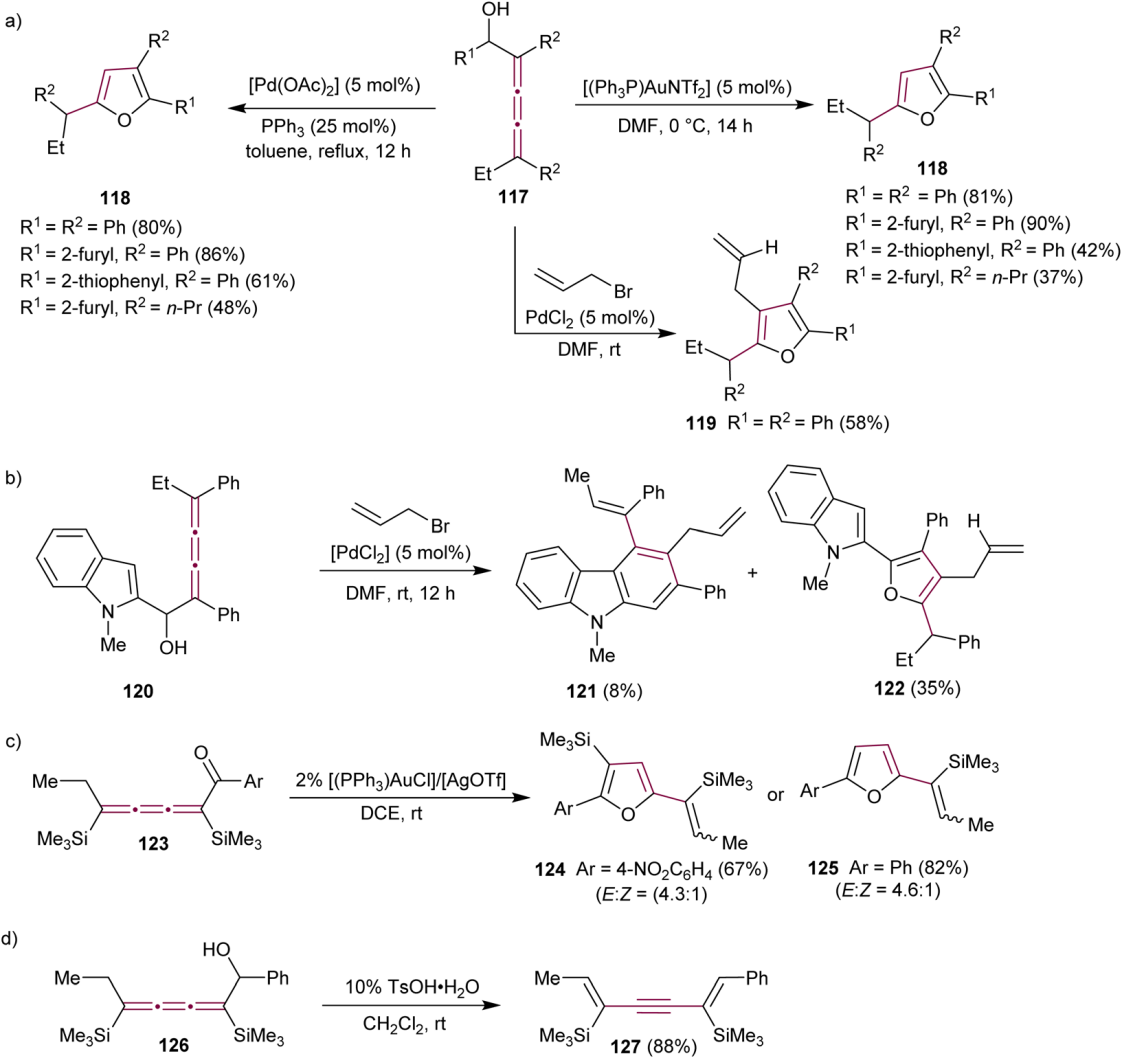
Reactions of (a) [3]cumulenol 117, (b) [3]cumulenol 120, (c) [3]cumulenone 123, and (d) [3]cumulenol 126.^160,161^

Alcaide *et al.* reported the effect of several metal catalysts on the cycloetherification reaction of 2,3,4-trien-1-ols 117, motivated by the prevalence of oxacyclic structures (*e.g.*, furan derivatives) in many biologically active natural compounds (Scheme 26a).^159,160^ The study identified several distinct reaction conditions that can be implemented to make tri- and tetrasubstituted furans 118 and 119 in a controlled manner.^160^ A carbocyclization/coupling sequence was also explored in indole-substituted [3]cumulene 120, leading to the formation of 2,3,4-trisubstituted carbazoles 121 as the minor product together with furan 122 (Scheme 26b).

Liu and coworkers showed that cycloisomerization of [3]cumulenone 123 catalyzed by gold(i) complexes yields tri- and disubstituted vinyl furans 124 and 125 as the major and minor products, respectively (Scheme 26c).^161^ The same study reported that Brønsted acid-catalyzed isomerization of [3]cumulenol 126 to 1,5-dien-3-yne 127 is a mild and selective synthetic method for entry into this useful class of compounds (Scheme 26d).

**Scheme 27:**
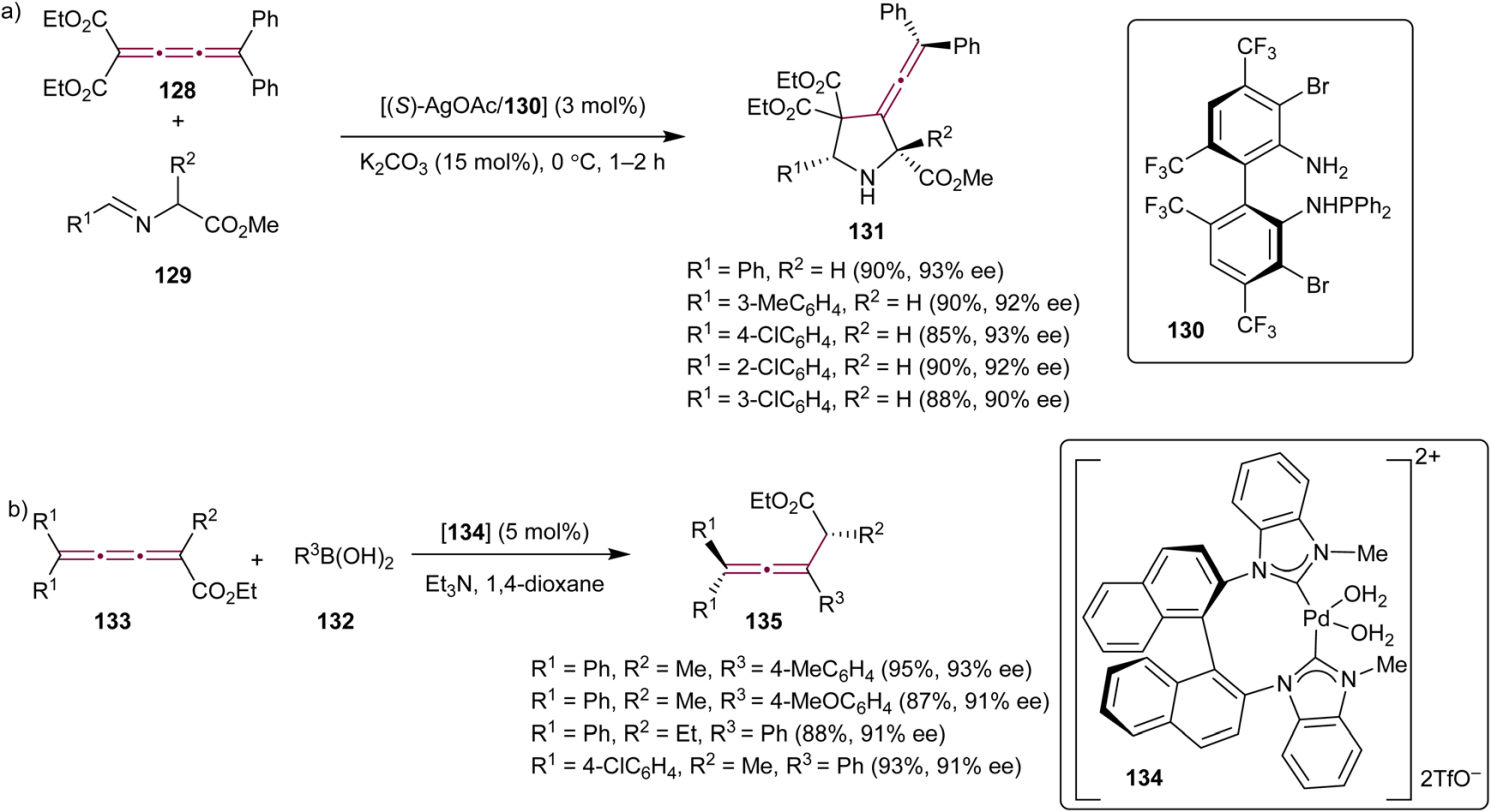
Stereoselective organometallic reactions of [3]cumulenes (a) 128 and (b) 133.^162,164^

In the context of constantly growing demand for synthetic methods capable of producing enantiopure pharmaceutical compounds, the exploration of organometallic reactions involving cumulenes has also been targeted with the objective of achieving high stereoselectivity in the construction of complex molecular systems. In 2011, Xue *et al.* described a catalytic asymmetric 1,3-dipolar cycloaddition reaction between tetrasubstituted cumulene 128 and azomethine ylides 129 (Scheme 27a).^162^ This transformation was catalyzed by the Ag(i) complex with the ligand TF-BiphamPhos 130 and gave 3-vinylidene-pyrrolidine derivatives 131 in high yields, with enantioselectivities reaching 93% ee.

Catalytic, asymmetric conjugate addition of organoboron reagents to alkenes or allenes has been a well-established and efficient approach for creating chiral, enantioenriched molecules from simple, often achiral, building blocks.^163^ Shi and coworkers have expanded this methodology to include cumulenes. The authors reported a conjugate addition of arylboronic acids 132 to cumulenes 133 (Scheme 27b) catalyzed by the axially chiral *N*-heterocyclic carbene Pd(ii) complex 134.^164^ This transformation resulted in the formation of allenes 135 with good to excellent yields and moderate to good enantioselectivities.

**Scheme 28:**
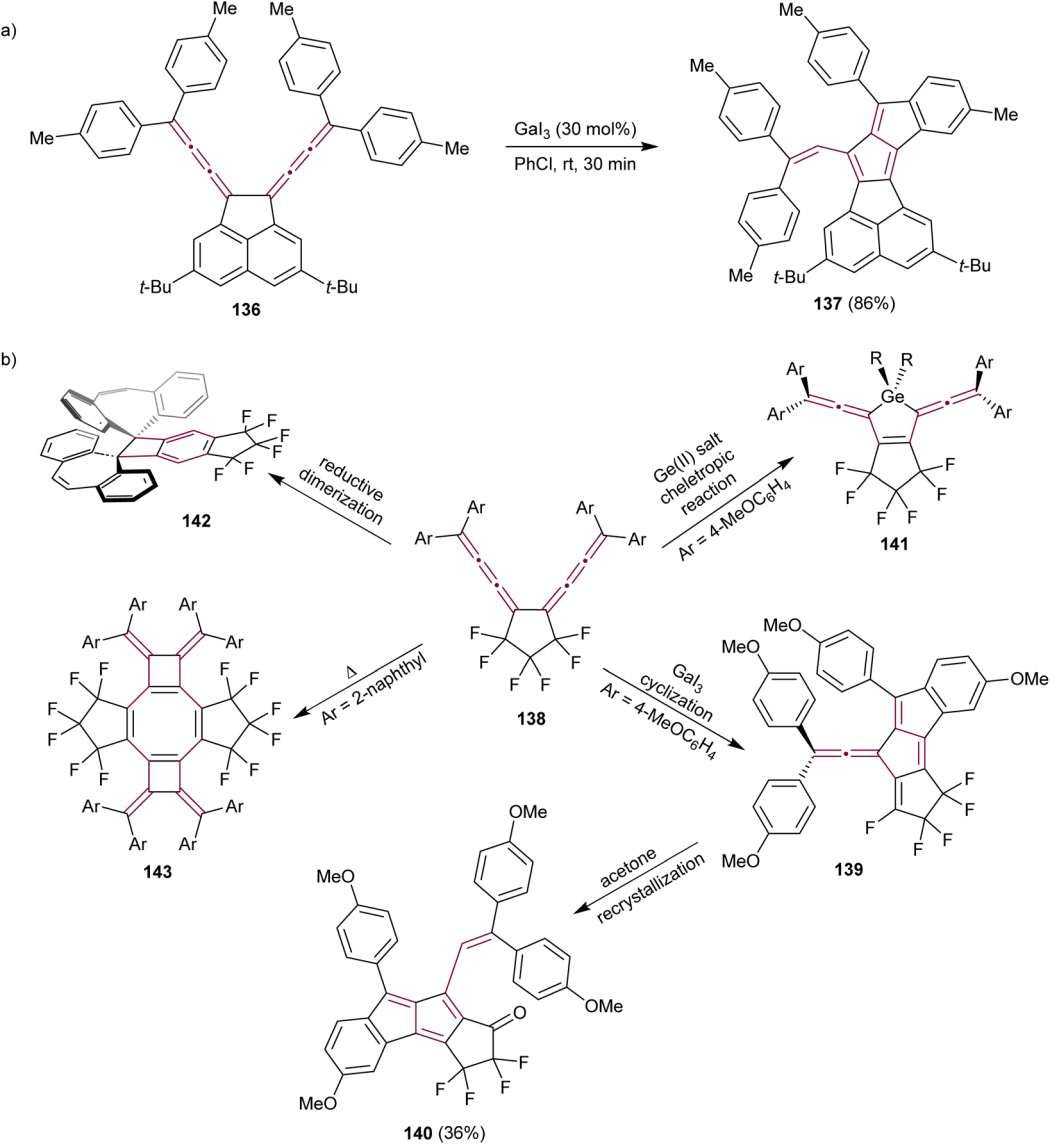
Catalytic reactions of bis[3]cumulenes (a) 136 and (b) 138.^165,166^

Conjugated bis[3]cumulene 136 is composed of two cumulene units linked by a single bond (Scheme 28a). Owing to the presence of numerous unsaturated carbon atoms within their structure, they are excellent candidates for complex domino reactions. Konishi *et al.* reported the Lewis-acid catalyzed cycloisomerization of conjugated bis[3]cumulene 136 that leads to the formation of functionalized pentalenes 137 (Scheme 28a).^165^ The rigid acenaphthene backbone enables effective communication between the two exocyclic [3]cumulenes, facilitating the one-pot construction of pentalenes 137 through the catalytic cycle involving Lewis-acid, such as GaI_3_. The resulting diareno[a,f]pentalene derivative 137 is a stable purple solid with a nearly planar structure, as shown by X-ray crystallographic analysis.

The same research group have investigated the reactivity of hexafluorocyclopentane-bridged bis[3]cumulenes 138 (Scheme 28b).^166^ Electron-withdrawing fluorine atoms enhance the stability of cumulenes and the flexible aliphatic cyclopentane minimizes steric hindrance relative to the acenaphthene backbone, resulting in new reactivity patterns. The reaction of 138 with the Lewis acid GaI_3_ results in extended fulvene 139. Interestingly, fulvene 139 transforms to pentalene 140 during crystallization in acetone when functionalized with electron-donating aryl substituents, such as *p*-anisyl. Biscumulenes 138 serve as ligands when treated with GeCl_2_ and form stable germacycles 141. When the cumulene bears dibenzocycloheptatriene as an endgroup, a reductive Bergman-type cyclization takes place to produce benzocyclobutane 142, featuring an extraordinarily long single C–C bond of 1.75 Å between dibenzocycloheptatriene moieties. They also showed that the bis[3]cumulene 138 endcapped with 2-naphthyl groups undergoes thermal dimerization to form the functionalized cyclooctatetraene derivative 143. This study beautifully demonstrated the potential of cumulenes as synthons, showing that from one cumulene-based scaffold, a range of complex π-conjugated structures can be synthesized by simply altering the reagents and reaction conditions.

### Miscellaneous reactions

In the previous sections, we provide detailed discussions on cyclooligomerization, cycloaddition, and organometallic reactions involving odd [*n*]cumulenes. This section aims to integrate and examine the diverse array of additional reaction types involving cumulenes that do not fit into the other groups. They include reactions involving acids, bases, and halogens, as well as epoxidation reactions and various other processes. This will further highlight the synthetic utility and functional versatility of [*n*]cumulenes in organic chemistry.

**Scheme 29:**
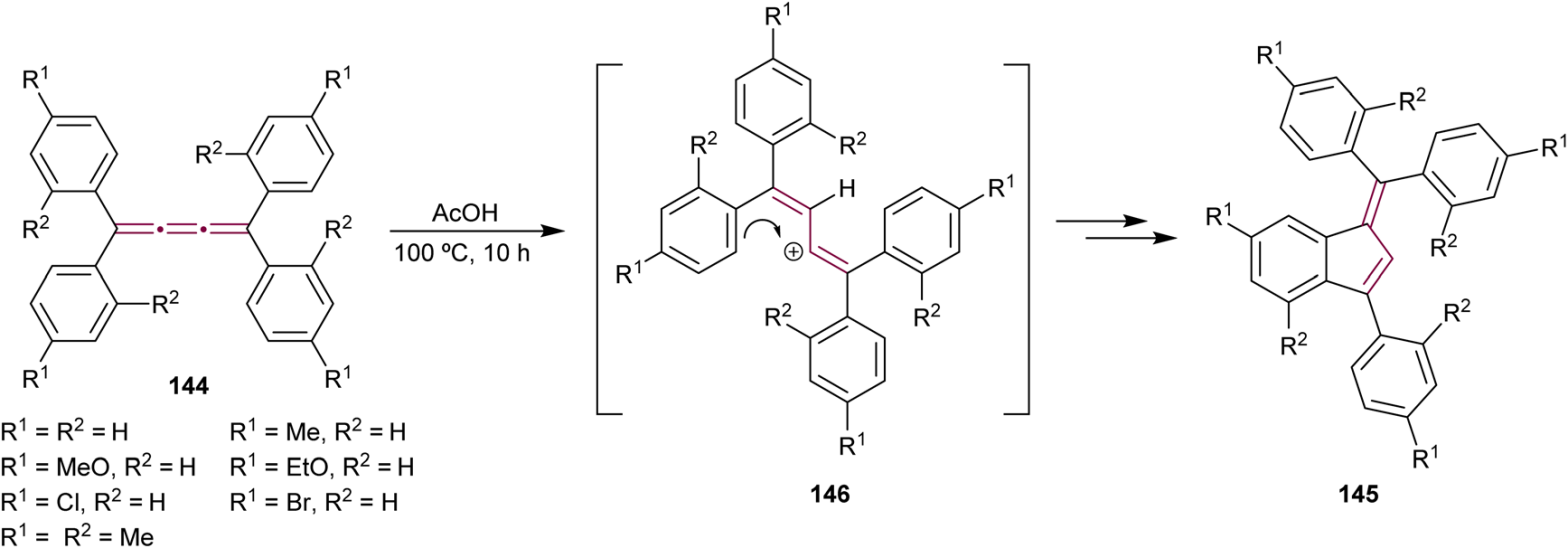
Acid-mediated rearrangement of tetraaryl[3]cumulenes 144 to benzo[*d*]fulvenes 145.^170^

Benzofulvenes are a valuable class of compounds widely used as precursors in total synthesis, organometallic chemistry, and materials science.^167–169^ Given their synthetic utility, numerous strategies have emerged for assembling the benzofulvene scaffold. As discussed earlier, synthesis of benzofulvenes can be achieved through the Pd-catalyzed arylation of [3]cumulenes (Scheme 24).^155–157^ However, already in 1950, Brand reported that tetraaryl[3]cumulenes 144 cyclize to benzo[*d*]fulvene 145 upon heating in acetic acid at 100 °C (Scheme 29).^170^ The reaction likely proceeds *via* protonation of cumulene, producing the carbocation intermediate 146, which then undergoes an intramolecular Friedel–Crafts arylation to form a five-membered ring. The structure of 145 and the reaction mechanism were supported by subsequent studies, which demonstrated that treatment of various [3]cumulenes with acid consistently produced similar structures.^171,172^

**Scheme 30:**
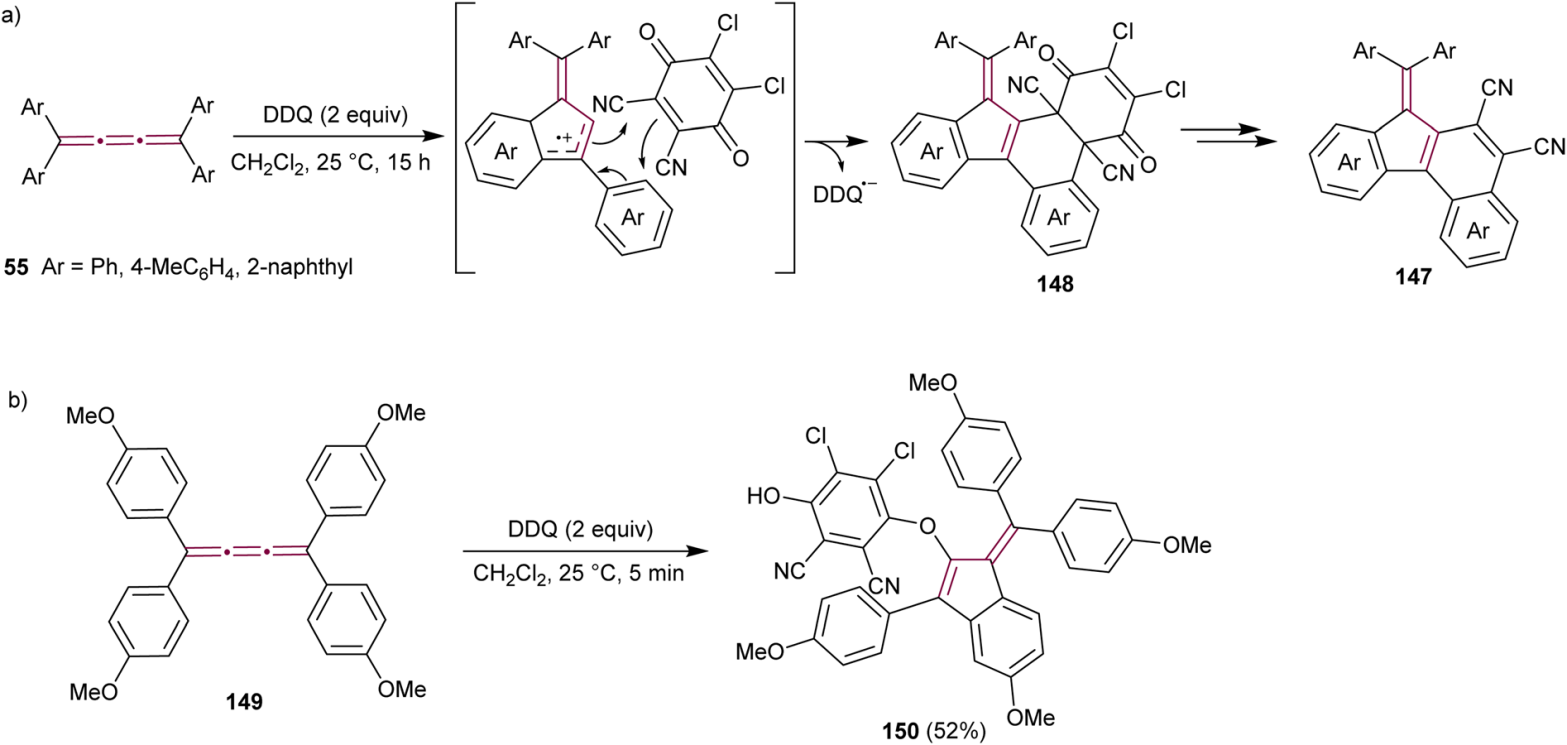
Reactions of DDQ with tetraaryl[3]cumulenes (a) 55 and (b) 149.^173^

Diederich and coworkers have reported the synthesis of cyanated benzo[*c*]fluorenes 147 from the reaction of tetraaryl[3]cumulenes 55 with 2,3-dichloro-5,6-dicyano-1,4-benzoquinone (DDQ), a common oxidizing agent (Scheme 30a).^173^ The proposed mechanism begins with a single electron transfer from the cumulene to DDQ, followed by intramolecular cyclization to form the fulvene core. Cycloaddition of this intermediate with DDQ results in 148, which was isolated and its structure confirmed by single-crystal X-ray diffraction analysis. Subsequent elimination of 2,3-dichloromaleate yields the final product, fulvenes 147. When substituted with electron-donating anisyl groups, cumulene 149 undergoes the same cyclization step to a benzofulvene intermediate, but the reaction is terminated by the addition of DDQ to produce benzofulvene 150 (Scheme 30b).

**Scheme 31:**
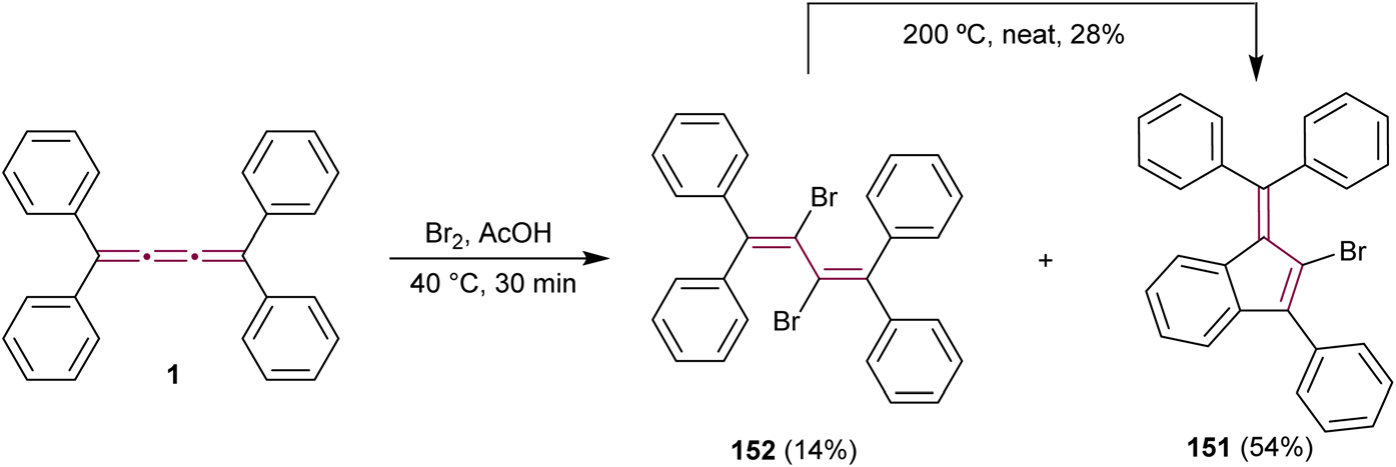
Bromination of tetraphenyl[3]cumulene 1.^38^

The outcome of halogenation reactions of cumulenes differs substantially from that of alkenes. For instance, bromination of tetraphenyl[3]cumulene 1 yields a mixture of products with compositions that vary depending on the reaction conditions.^38^ Woliński reports that, in a polar protic solvent such as acetic acid, the primary product of reaction with bromine is benzofulvene 151, implicating polar or ionic intermediates in its formation. Conversely, carrying out the reaction in an apolar solvent like CCl_4_ and catalyzed by benzoyl peroxide favors the formation of butadiene 152, which represents the expected product upon bromination of a double bond.^38^ Additionally, 2,3-dibromobutadiene 152 can be transformed into fulvene 151 upon heating to 200 °C in the absence of solvent (Scheme 31).

**Scheme 32:**
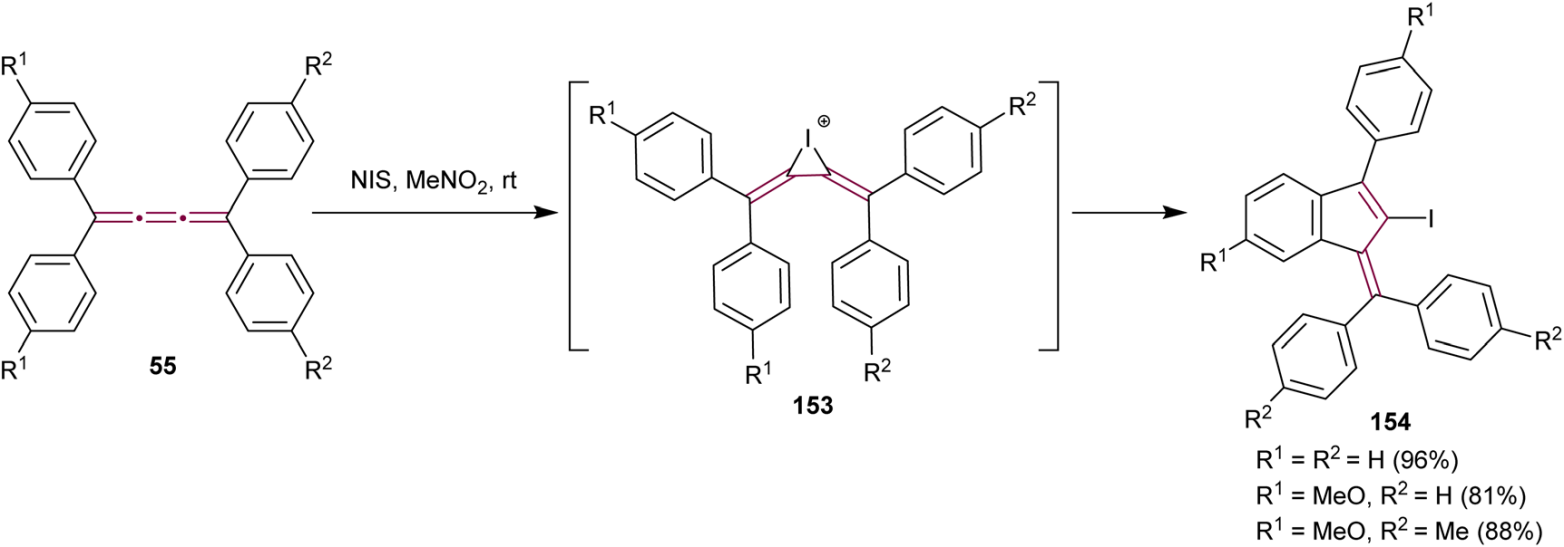
Synthesis of iodofulvenes 154 from tetraaryl[3]cumulenes 55.^174^

In 2021, Yagishita *et al.* described the synthesis of the benzofulvene framework 154*via* electrophilic iodocyclization of tetraaryl[3]cumulenes 55 (Scheme 32).^174^ The reaction utilizes *N*-iodosuccinimide (NIS) to generate iodonium intermediates 153, which undergo rearrangement forming iodobenzofulvenes 154. The cyclization step occurs regioselectivity on a more electron-rich aromatic ring when cumulenes are unsymmetrically substituted. Under optimized conditions, the equivalent reaction with NBS instead of NIS gives the analogous bromo-derivatives of benzofulvenes 154. The NIS transformation is subsequently shown to proceed efficiently under solvent-free mechanochemical conditions.^175^

**Scheme 33:**
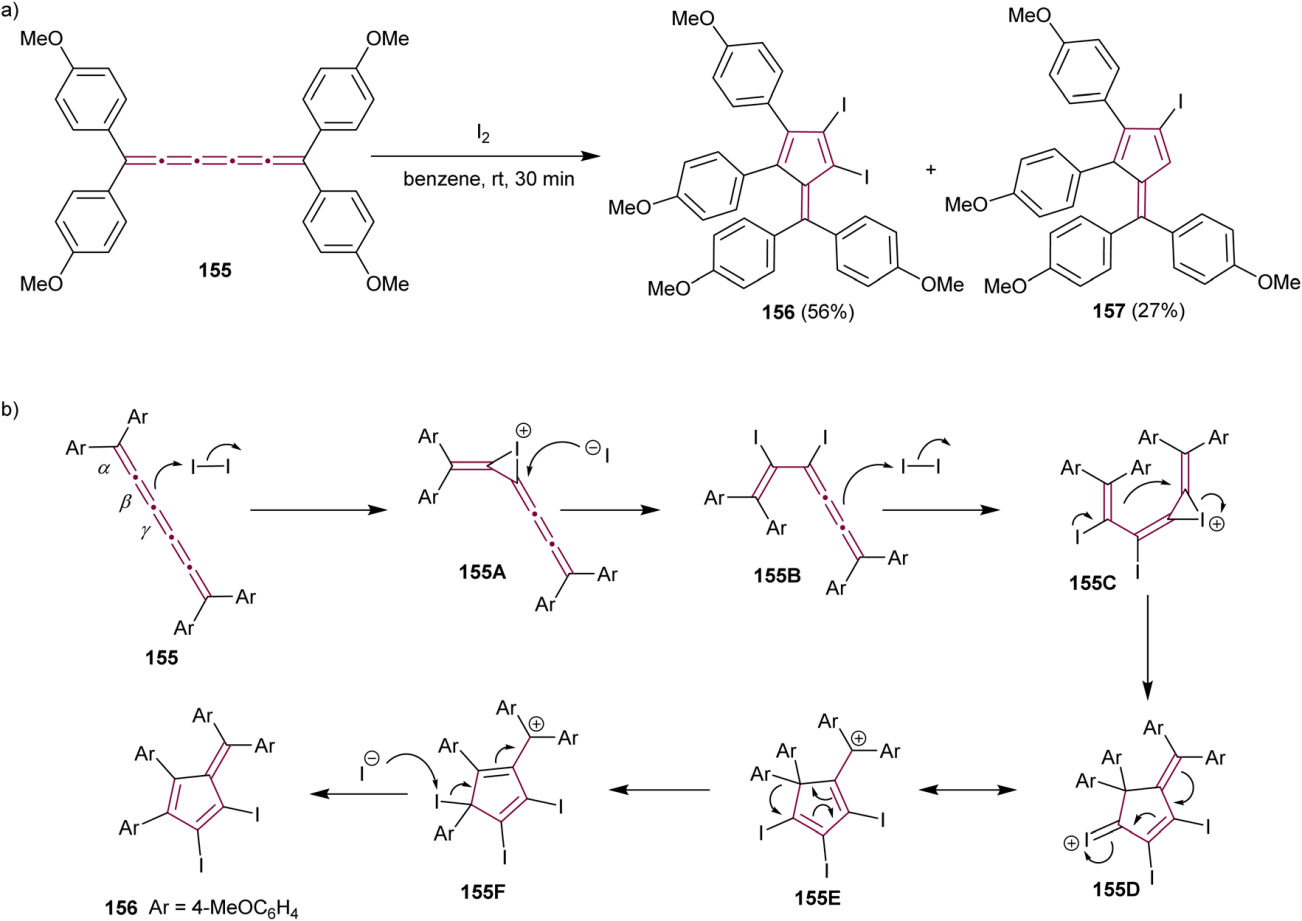
(a) Synthesis of iodofulvenes 156 and 157 from [5]cumulene 155 and (b) the proposed mechanism.^176^

The formation of highly functionalized fulvenes through the reaction of tetraaryl[5]cumulene 155 with elemental iodine is depicted in Scheme 33a. This reaction yields diiodofulvene 156 and iodofulvene 157 in varying ratios, depending on reaction conditions.^176^ Mechanistic studies suggest that iodonium ion intermediate 155A is generated by the reaction of 155 with iodine at the β-bond (Scheme 33b). The subsequent nucleophilic attack of the iodide ion on 155A produces diiodide 155B, which further reacts with another molecule of iodine to form the iodonium intermediate 155C. Subsequent cyclization, migration of the aryl group, and elimination of iodonium give the final diiodofulvene 156. When the reaction time is increased, in either MeNO_2_ or benzene, the authors noted a decreased yield of 156 and increased yield of 157. This suggests that compound 157 is generated from compound 156, presumably by the reaction of 156 with HI generated *in situ*. The synthetic utility of iodofulvenes 154 and 157 is showcased in both reports through functionalization *via* a cross-coupling reactions that produced unique π-expanded fulvenes.^176^

**Scheme 34:**
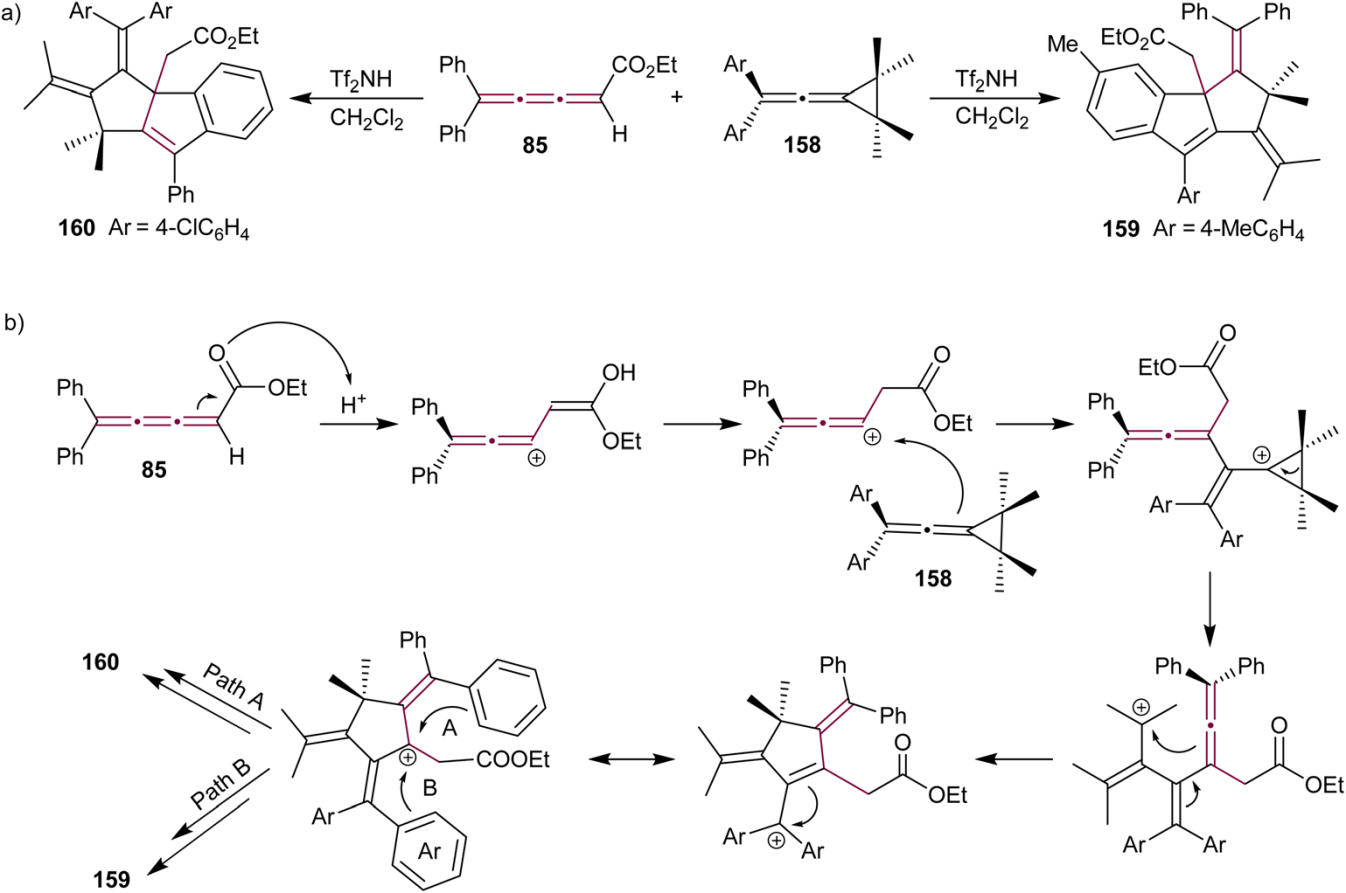
(a) Reaction of [3]cumulene 85 with vinylidenecyclopropane 158 and (b) the proposed mechanism.^172^

Vinylidene cyclopropanes are stable molecules, characterized by an exocyclic allene moiety linked to a cyclopropane ring and provide synthetic access to a range of unique hetero- and polycyclic compounds.^177^ Shi and Li reported a triflic imide-catalyzed reaction cascade between diarylvinylidene cyclopropanes 158 and [3]cumulene 85 (Scheme 34a).^172^ The final product of this cascade annulation reaction varies depending on the substitution of the vinylidene cyclopropane substrate. Vinylidene cyclopropanes 158 substituted with electron-donating groups typically favor the formation of 159, while those substituted with electron-withdrawing groups predominantly yield 160. The proposed mechanism involves activation of [3]cumulene 85 by Brønsted acid Tf_2_NH, leading to the formation of a carbocation intermediate and subsequent nucleophilic attack by diarylvinylidene cyclopropane 158 (Scheme 34a). This is followed by an intramolecular cyclization, which yields the product 159 or 160 depending on the nature of aryl groups.

**Scheme 35:**
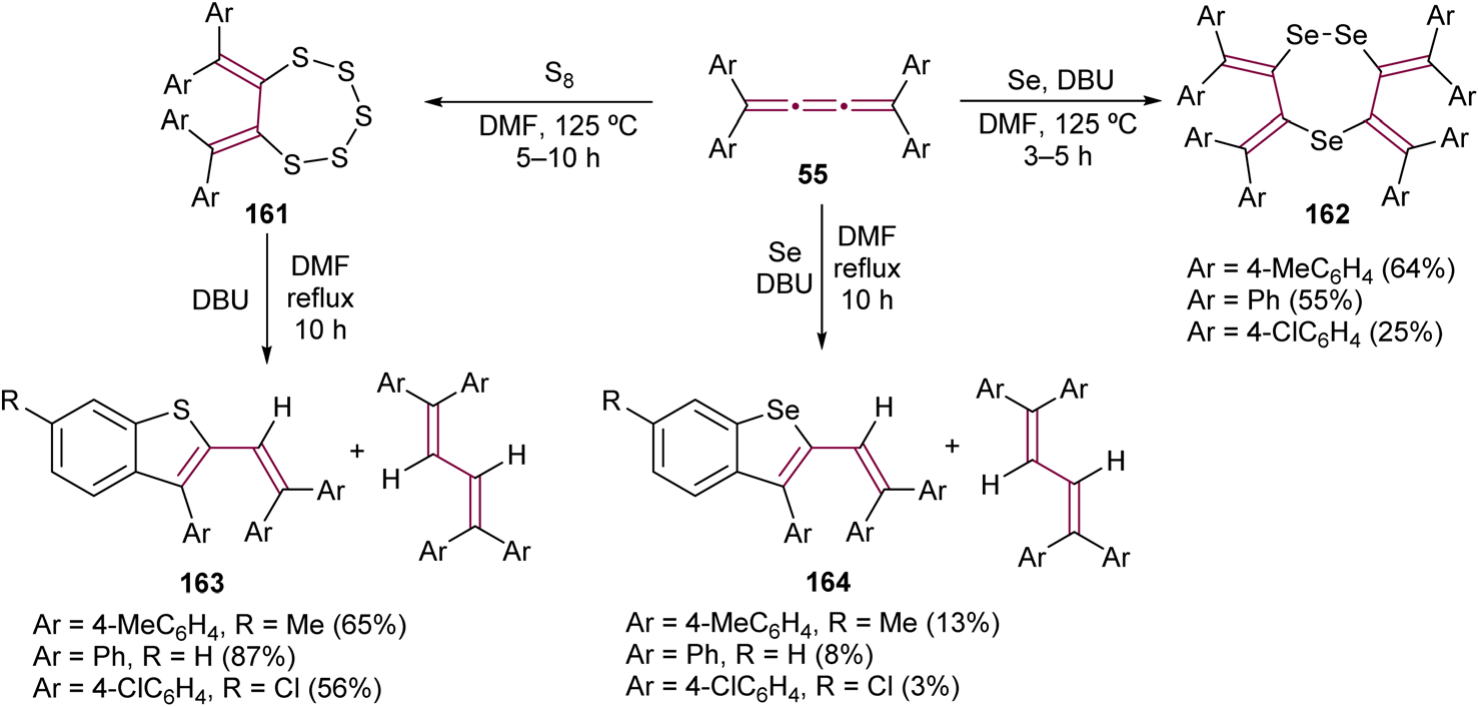
Reactions of tetraaryl[3]cumulenes 55 with elemental sulfur and selenium.^178^

Interesting outcomes have been reported by Ando and co-workers from the reaction of tetraaryl[3]cumulenes 55 with elemental sulfur and selenium (Scheme 35).^178^ After heating the [3]cumulenes 55 with elemental sulfur for several hours, macrocyclic products 161 are obtained. [3]Cumulenes 55 are inert to elemental selenium and required base 1,8-diazabicyclo[5.4.0]undec-7-ene (DBU) to give 1,2,5-triselenephane derivatives 162. When compounds 161 and 162 are heated for extended periods in the presence of DBU, the major product is a 1,3-butadiene in both cases, the product of reduction. However, more intriguing are the minor products, benzothiophene 163 and benzoselenophene 164, which form *via* intramolecular cyclization cascades. When the [3]cumulene is substituted with adamantylidene groups, the product of the sulfurization reaction is analogous to 161.^179^

**Scheme 36:**
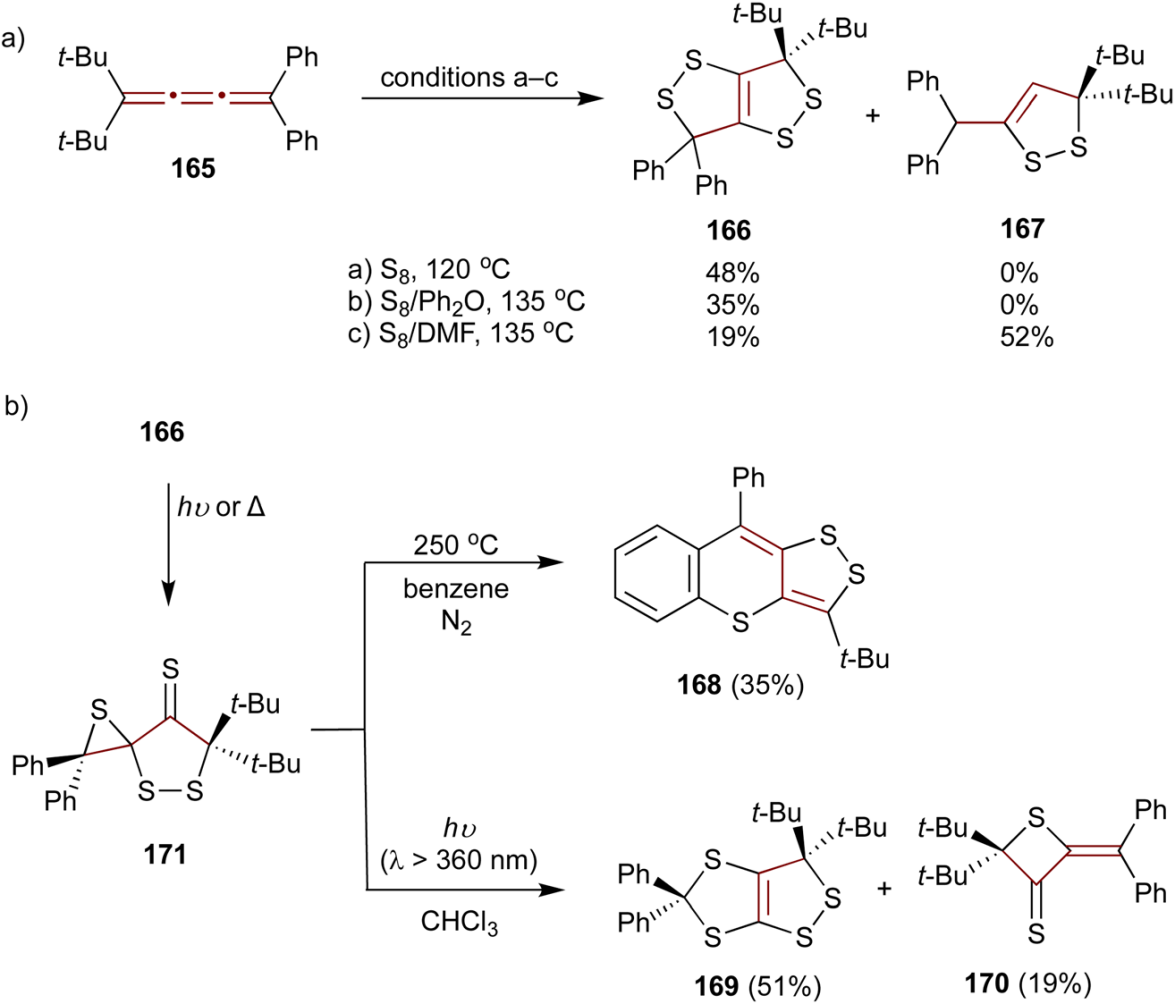
(a) Sulfurization of [3]cumulene 165 and (b) transformations of 166.^178–180^

As later reported by Ando and coworkers, the sulfurization of unsymmetrical [3]cumulene 165 proceeds quite differently compared to symmetric tetraaryl- (55) or adamantylidene-substituted derivatives.^178–180^ When 165 is heated neat with molten sulfur or in a solution of diphenyl ether, the exclusive product of this reaction is 1,2-dithiolo-1,2-dithiole 166. However, performing the sulfurization in DMF leads to 1,2-dithiole 167 as the major product (Scheme 36a). Interestingly, upon heating to 250 °C in benzene under N_2_, 1,2-dithiolo-1,2-dithiole 166 undergoes thermolysis producing an unusual polycyclic scaffold 168 (Scheme 36b). In contrast, upon UV irradiation (360 nm), 166 rearranges to 1,2-dithiolo-1,3-dithiole 169 and thietane-3-thione 170. Both of these transformations are postulated to proceed *via* a spirothiirane intermediate 171.

**Scheme 37:**
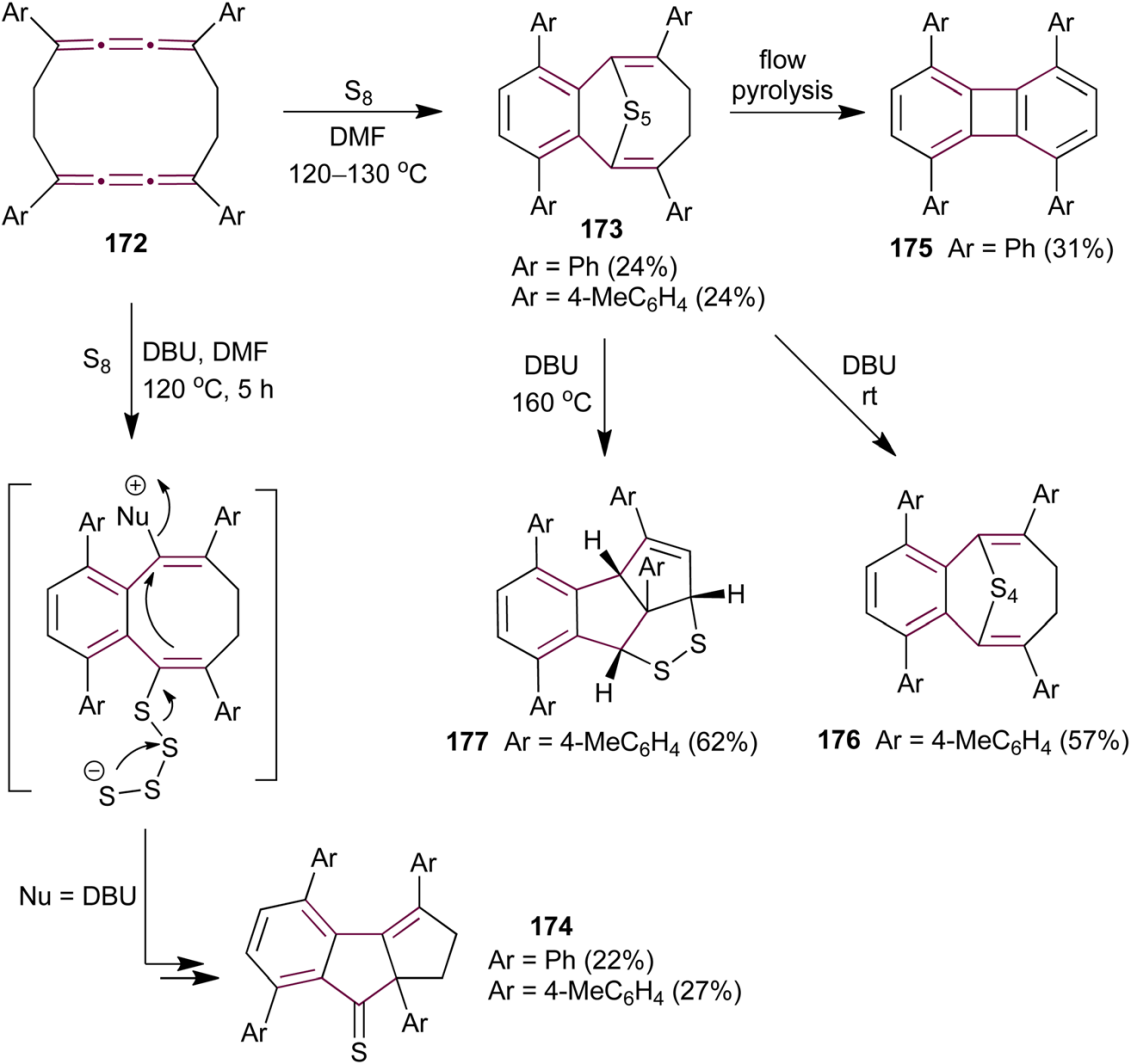
Sulfurization of biscumulene 172.^181^

Ando and coworkers have extended their investigation of cumulene sulfurization to the cyclic biscumulene 172, aiming to induce a transannular bridging reaction.^181^ Treating macrocycle 172 with an excess of sulfur in DMF at 120–130 °C for 10 hours yields cyclic polysulfides 173 (Scheme 37). In contrast, performing the same reaction in the presence of DBU affords cyclopentenethione 174 as the sole product. Single-crystal X-ray diffraction analysis unambiguously confirms the structures of both products. To identify whether 173 is the intermediate in the formation of 174, the authors subjected polysulfide 173 to thermal decomposition under flow pyrolysis conditions, which affords the biphenylene derivative 175. However, when pentasulfide 173 is treated with DBU in DMF at room temperature, it produces the tetrasulfide 176, whereas heating 173 to 160 °C generates the fused dithiolane 177 (Scheme 37). These observations indicate that the mechanism leading to the formation of 174 is fundamentally different from that for the formation of 173 during the sulfurization of biscumulene 172, presumably involving ionic sulfur species under basic conditions.

**Scheme 38:**
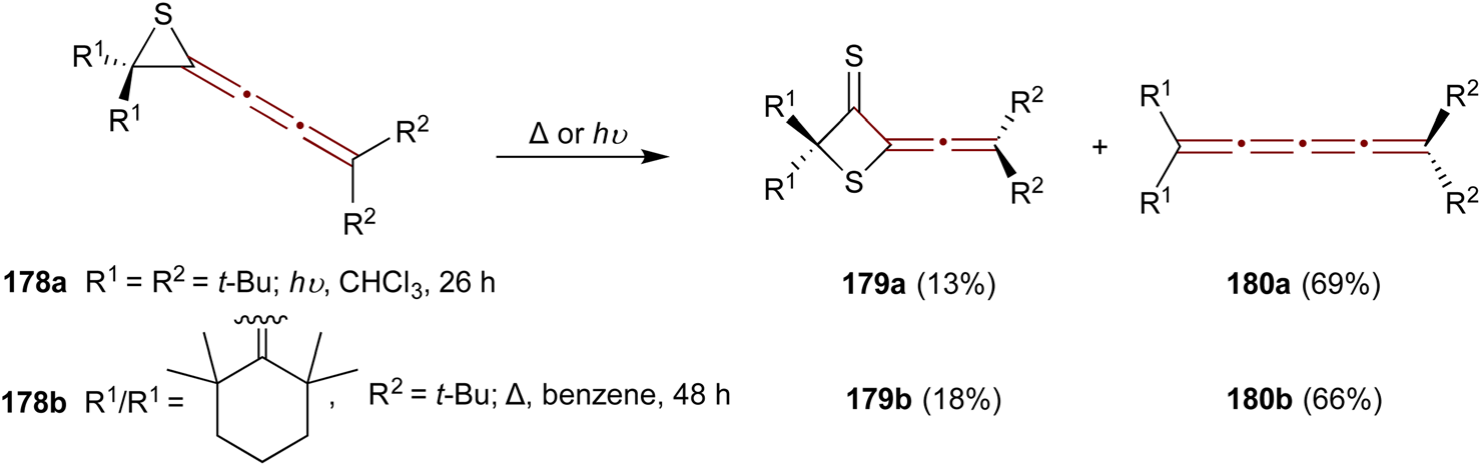
Transformations of 1-episulfides 178 under thermal and photochemical conditions.^182^

A study by Ando and coworkers in 1989 reports a convenient synthesis of episulfide [3]cumulenes 178a,b and investigates their reactivity under thermal and photochemical conditions.^182^ Two transformations are predominant: conversion to thietanethiones 179 and desulfurization to [4]cumulenes 180 (Scheme 38). Irradiation of the *tert*-butyl-substituted episulfide 178a with UV light (300 nm) in chloroform at room temperature gives thietanethione 179a and tetraene 180a in 13% and 69% yields, respectively. When episulfide 178b is heated in benzene under reflux, 179b and 180b are produced in 18% and 66% yields, respectively. Mechanistic investigations suggest that the conversion of 178 to 179 proceeds *via* sulfur transfer involving diradical intermediates. Adding excess elemental sulfur during the thermolysis or photolysis of 178 fails to increase the yield of 179. Moreover, the absence of dimerization products suggests that the bulky substituents of 178 create steric hindrance, favoring sulfur transfer as the principal reaction pathway.

**Scheme 39:**
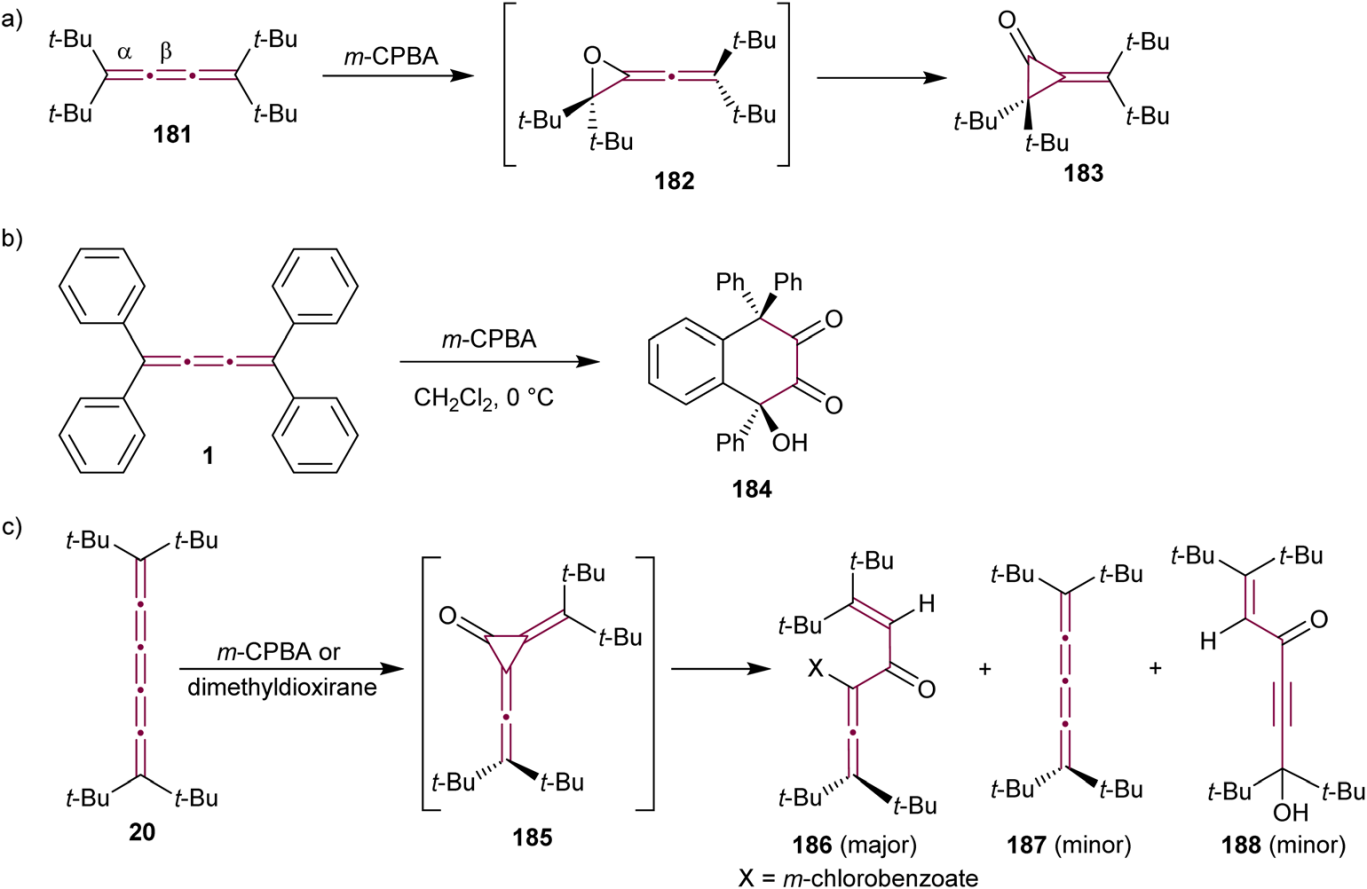
Oxidation reactions of odd cumulenes: (a) 181, (b) 1, and (c) 20.^185–187^

The reaction of allenes with peracids yields products with high synthetic utility.^183^ These reactions typically proceed through intermediate allene oxides (methyleneoxiranes), which then isomerize to form more stable cyclopropanones.^184^ Extending this idea to the oxidation of cumulenes has also yielded some unique molecular architectures. For instance, the reaction of tetra(*tert*-butyl)[3]cumulene 181 with *m*-chloroperbenzoic acid (*m*-CPBA) leads to epoxidation of the terminal α-bond, presumably producing epoxide 182, which readily isomerizes to cyclopropanone 183 (Scheme 39a).^185^ Subsequent transformation of 183 with various reagents facilitates the synthesis of allenes, ketenes, and epoxyketones. In contrast, the oxidation tetraphenyl[3]cumulene 1 with *m*-CPBA yields tetralindione 184, presumably *via* a double oxidation of the cumulene core followed by an intramolecular cyclization at the phenyl endgroup (Scheme 39b).^186^

In 1992, Crandall and coworkers explored the oxidation of higher cumulenes (Scheme 39c).^187^ Treatment of tetra(*tert*-butyl)[5]cumulene 20 with *m*-CPBA results in the transient formation of a cyclopropanone intermediate 185, which subsequently transforms mainly to allenic ketone 186. Minor quantities of [4]cumulene 187 and ketone 188 are also detected in the reaction mixture.

**Scheme 40:**
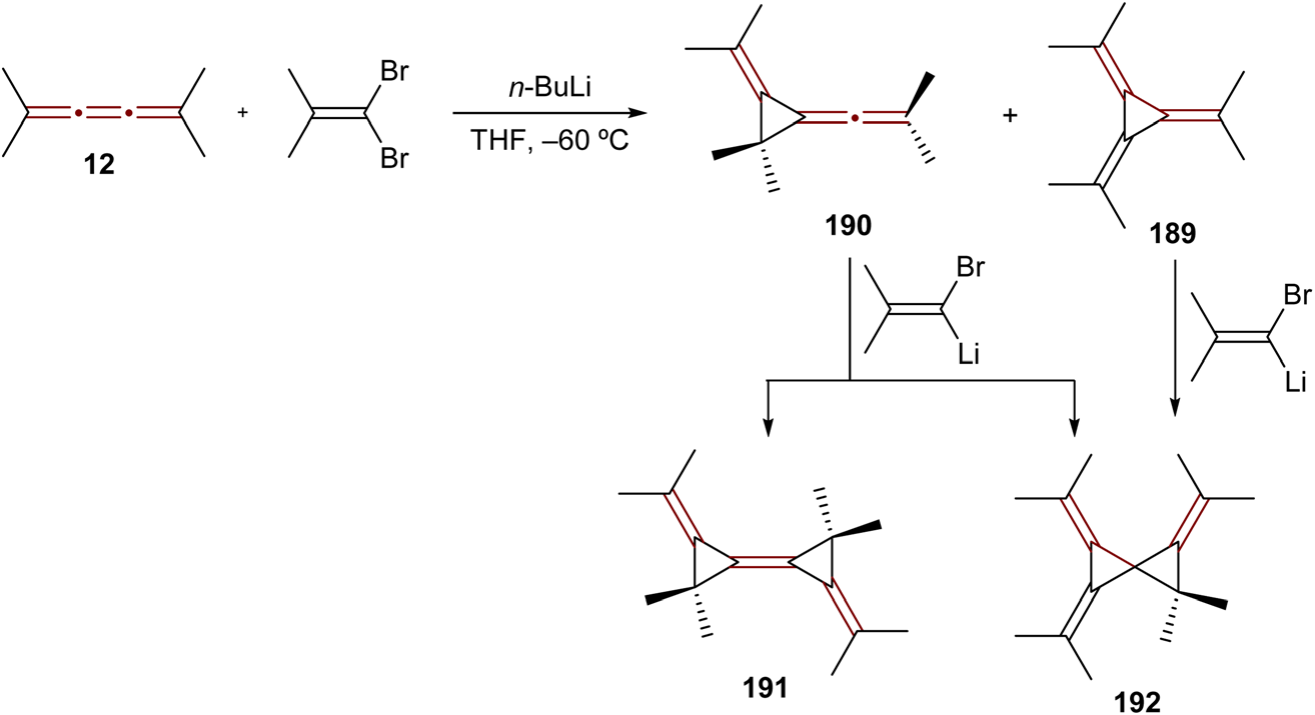
Reaction of tetramethyl[3]cumulene 12 with a vinylidene carbene.^188^

The reaction of [3]cumulenes with vinylidene carbenes has been explored, similar to oxidation reactions, in relation to the reactivity of an allene.^188^ The reaction of tetramethyl[3]cumulene 12 with the carbene generated from 1,1-dibromo-2-methylprop-1-ene mainly gives the expected adduct, [3]radialene 189, together with a product of rearrangement, allene 190 (Scheme 40). Compounds 189 and 190 react further with the alkenyllithium carbene to produce the rather exotic radialene 191 and spirocyclopropane derivative 192.

**Scheme 41:**
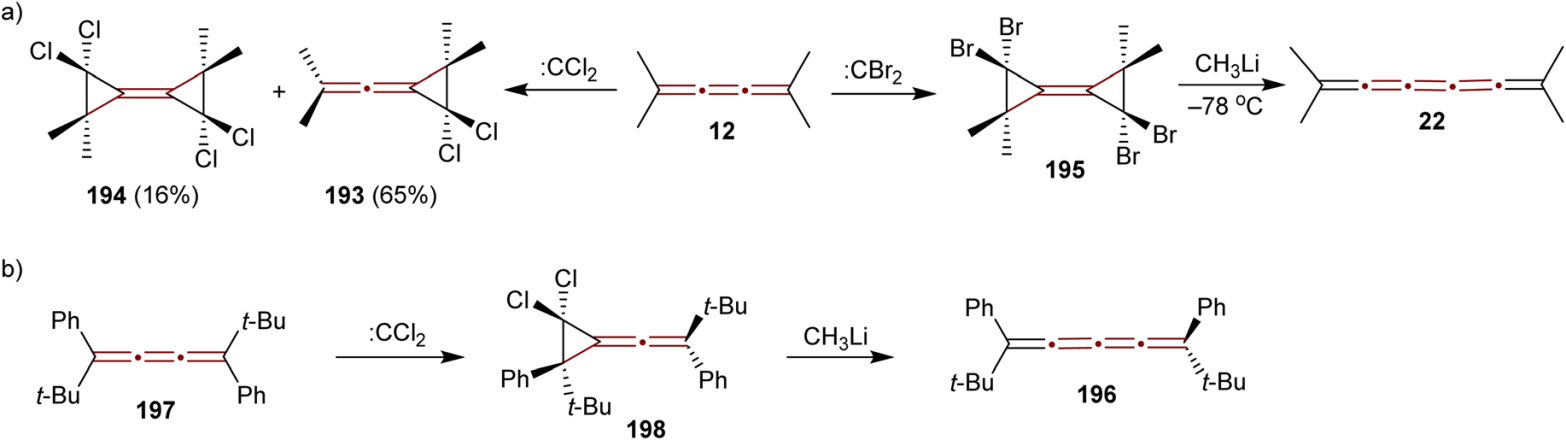
Halocarbene addition to [3]cumulenes: (a) 194 and (b) 197.^189,190^

A study conducted by Skattebøl in 1965 examined the addition of dihalocarbenes to tetramethyl[3]cumulene 12 (Scheme 41a).^189^ Addition of dichlorocarbene (generated from CHCl_3_ and KO*t*Bu) results in a mixture of mono- and diadducts, 193 and 194 in 65% and 16% yields, respectively. In contrast, using dibromocarbene (generated from CHBr_3_ and KO*t*Bu) yields a complex mixture, from which only a small amount of an identifiable diadduct 195 is isolated. Treating 195 with methyllithium at low temperatures gives tetramethyl[5]cumulene 22, a species too unstable for detailed characterization and identified only by UV-vis spectroscopy, the structure of which has later been confirmed spectroscopically by Scott and DeCicco (Scheme 7b).^59^

In 1974, Jochims and Karich described the first successful synthesis of [4]cumulene 196, starting from the corresponding [3]cumulene 197.^190^ Their strategy involved dichlorocarbene addition to 197, forming the intermediate 198, which was subsequently converted into the [4]cumulene 196 (Scheme 41b). The structure of 196 was confirmed by elemental analysis and a range of spectroscopic methods. [4]Cumulene 196 is surprisingly stable toward heat and oxygen, melting unchanged at 133 °C and showing no reactivity toward methyllithium.

### Reactive odd [*n*]cumulene intermediates

While the primary focus of this review is on the reactivity of stable, isolated cumulene species, it is important to acknowledge the broader reactivity landscape, which includes transient intermediates, strained cumulenes, and their organometallic variants. Although often unstable or highly specialized, these types of cumulenes play important roles in unique transformations and facilitate the construction of novel molecular architectures. Each represents a distinct and rich area of study, differing from classical cumulenes in bonding, stability, and reactivity. Although a detailed discussion of this area is beyond the scope of this review, a brief description is provided that offers essential context for understanding the broader aspects of cumulene chemistry.

Strained cumulenes are known as reactive intermediates, with their intrinsic ring strain significantly enhancing their reactivity. These species engage in a spectrum of transformations, providing platforms for the construction of complex molecular architectures. Their unique reactivity is comprehensively reviewed by Johnson.^191^ Notable examples include medium-sized cyclic cumulenes such as 1,2,3-cyclodecatriene, reported by Moore and Ozretich,^192^ as well as various other strained cyclic systems described by Johnson and co-workers.^193^

**Scheme 42:**
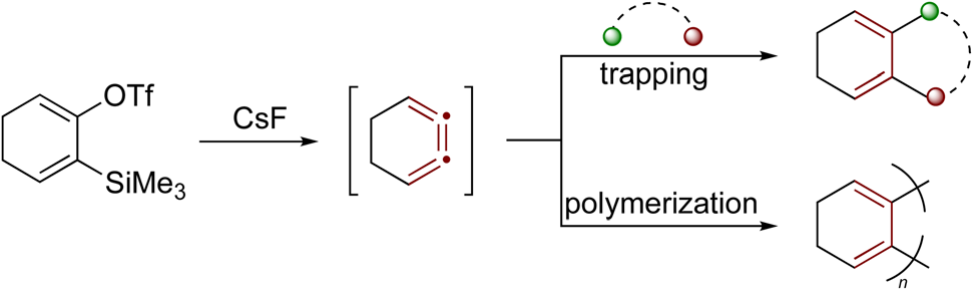
Reactivity of the strained cumulene intermediate: 1,2,3-cyclohexatriene.^14^

Another notable example of a strained cumulene is the benzene isomer 1,2,3-cyclohexatriene,^194–196^ featuring cumulated double bonds but lacking aromatic stabilization. These structural features result in significantly higher free energy (+101 kcal mol^−1^ relative to benzene) and enhanced reactivity.^140,197^ Recent studies by Garg and coworkers show that 1,2,3-cyclohexatriene, and its derivatives, participate in various strain-promoted reactions, including cycloadditions, nucleophilic additions, and σ-bond insertions (Scheme 42).^197,198^ For many organic chemists, 1,2,3-cyclohexatriene invites a comparison with *o*-benzyne, a strained aryne intermediate widely exploited for synthesis.^199,200^ Notably, complementary non-contact AFM and NMR studies have shown that benzyne might more accurately be described by a cumulenic bonding motif rather than the familiar ‘benzene-with-a-triple-bond’ depiction, a view that helps rationalize its characteristic reactivity.^201,202^

**Scheme 43:**
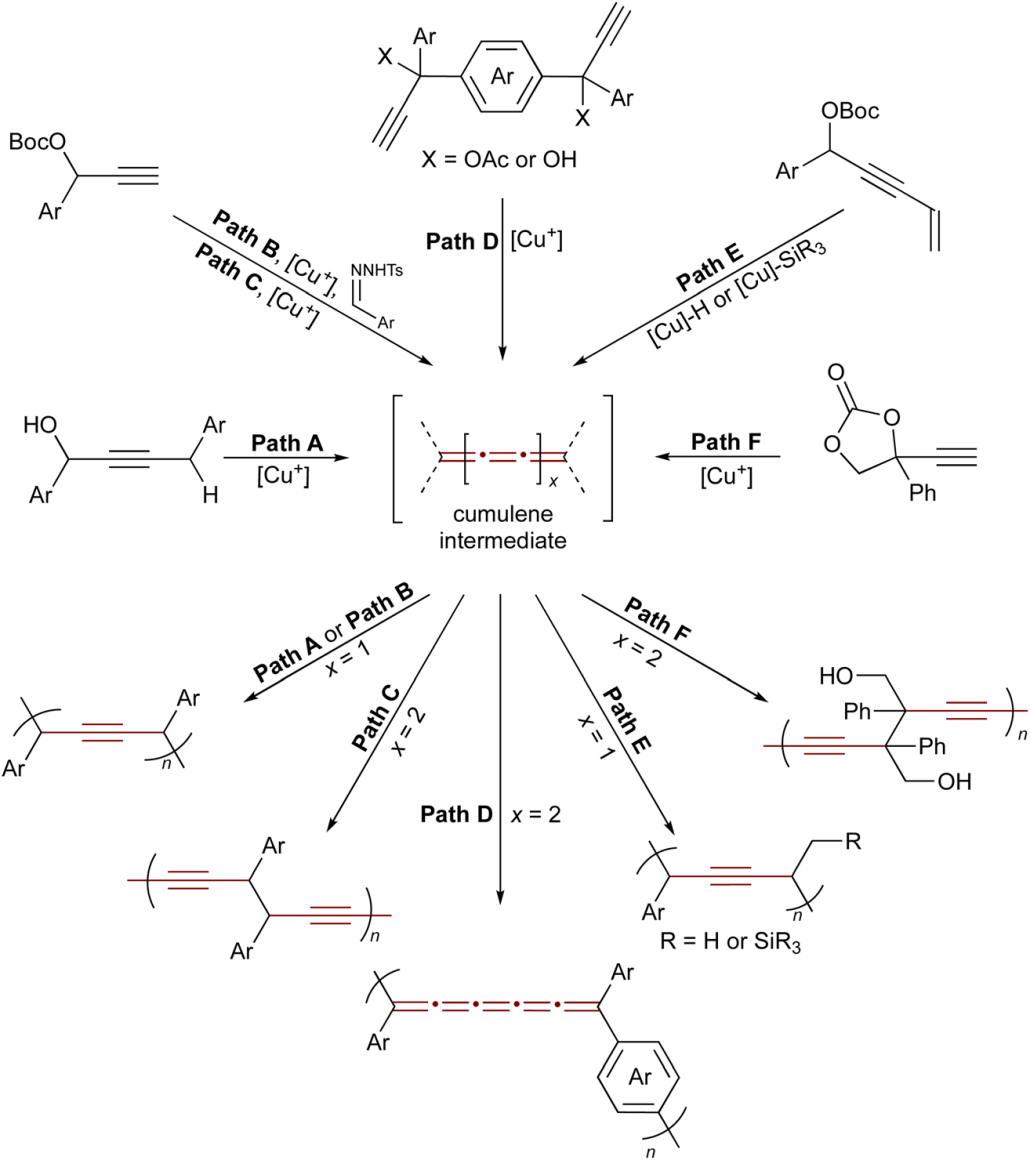
Copper-catalyzed polymerization of propargylic compounds. Path A: dehydrative polymerization of propargylic alcohols.^205^ Path B: copolymerization of propargyl carbonates and aryldiazomethanes.^208^ Paths C and D: synthesis of polydiynes *via* a dimerization and polymerization sequence of propargylic electrophiles.^204^ Path E: condensation polymerization enabled by the 1,2-regioselective hydro- and silylcupration of enyne-type propargylic electrophiles.^206^ Path F: ring-opening polymerization of cyclic carbonates.^207^

Reactive cumulene intermediates have also been exploited in polymer chemistry, where their cumulated π-systems confer high reactivity and enable selective chain-growth. Since 2020, Zhu and coworkers have been developing a family of copper-catalyzed polymerizations of propargylic substrates as a promising strategy to access unique, all-carbon, alkyne-rich polymer backbones *via* reactive cumulene intermediates.^203^ Key examples are summarized in Scheme 43 and illustrate how transient cumulene intermediates enable the creation of diverse polymer architectures.^204–208^ Representative examples include the dehydrative polymerization of propargylic alcohols,^205^ copolymerization of propargyl carbonates with aryldiazomethanes,^208^ stepwise-chain growth condensation routes to conjugated [5]cumulene polymers,^204^ polymerization *via* catalytic element-cupration,^206^ and ring-opening transformations of cyclic carbonates.^207^ By tuning propargylic substrates such as carbonates, alcohols, or acetates along with reaction conditions and external nucleophiles, a wide variety of polymeric alkynes and cumulene-containing frameworks have been realized.

Organometallic chemistry of cumulenes encompasses both short-lived cumulene-containing intermediates and isolable complexes (including metallacyclocumulenes), forming a substantial field in its own right. Coordination with transition metals can stabilize and polarize cumulenic frameworks, unlocking distinctive reactivity relevant to synthesis and catalysis, including selective C–C activation, migratory insertion/fragmentation sequences, and cross-coupling pathways. As this area has been comprehensively surveyed, we refer readers to reviews for a detailed coverage of bonding models, preparative strategies, and representative applications.^209,210^

### Even cumulenes

Even [*n*]cumulenes (*n* = 4, 6, 8…) feature a profoundly different π-electron system compared to odd [*n*]cumulenes (*n* = 3, 5, 7…) due to the presence of two degenerate, orthogonal π-electron systems (see Fig. 1).^211^ A close structural relative to even [*n*]cumulenes, allenes, are valuable precursors in modern organic synthesis due to their π-electron-rich systems and intrinsic axial chirality (depending on the substitution pattern of endgroups). The reactivity and chemistry of allenes have been discussed in numerous reviews^212–215^ and will not be revisited here. Extending the chemistry of allenes to longer even [*n*]cumulenes has been sporadic, likely due to the inherent instability of these unsaturated substrates. To date, [4]cumulenes represent the longest even [*n*]cumulenes successfully isolated; a single example of *in situ* formation of a [6]cumulene has also been reported (*vide infra*).^216,217^ With the development of endgroups designed to improve stability, the breadth of isolable even [*n*]cumulenes has been slowly expanded, allowing for the study of their reactivity. Key transformations of even [*n*]cumulenes are similar to those of odd [*n*]cumulenes and include cyclooligomerization, cycloaddition, reactions with metals, hydrogenation, halogenation, oxidation, as well as other miscellaneous reactions encountered during their synthesis.

### Cyclodimerization

**Scheme 44:**
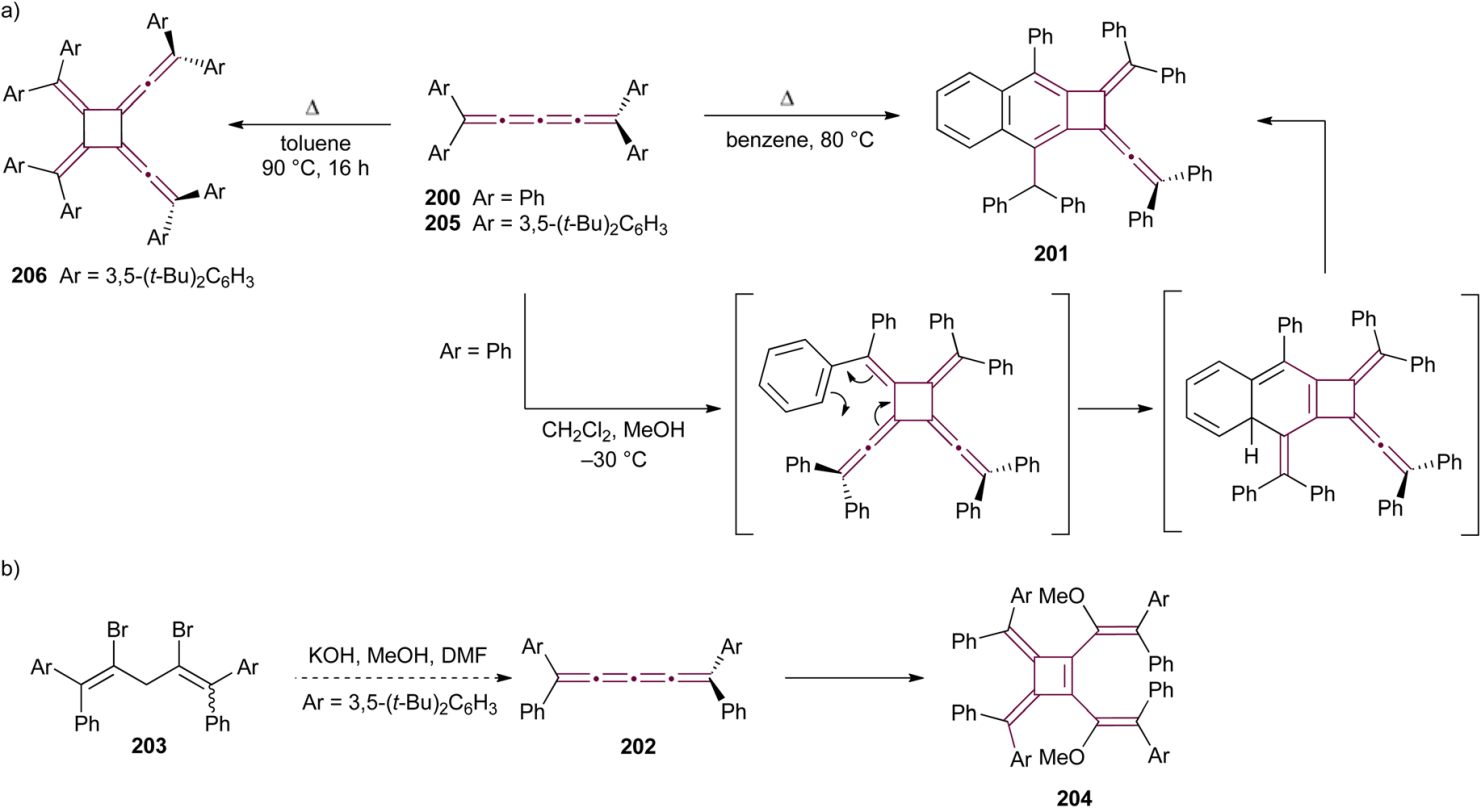
(a) Intermolecular dimerization of tetraphenyl[4]cumulene 200 and tetra(3,5-di-*tert*-butyl)phenyl[4]cumulene 205. (b) Formation of dimer 204 during the attempted synthesis of [4]cumulene 202.^211,220,221^

Shortly after Shechter and Tiers confirmed the structure of the dimerization product from tetraphenyl[3]cumulene 1, originally reported by Brandt in 1921,^8,43^ the striking photostability of tetraphenyl[4]cumulene 200 was highlighted by Fischer and Fischer.^218^ They attributed this behavior to two independent molecular π-orbitals in the [4]cumulene framework and the correspondingly larger HOMO–LUMO gap characteristic of even [*n*]cumulenes. The synthesis of 200 was independently achieved by Kuhn,^219^ Fischer,^218^ and then Ratts,^220^ although their observations differed. While Kuhn did not report dimerization, both Fischer and Ratts suggested that 200 undergoes thermal dimerization. Unlike 1, which converts to radialene 6, Fisher noted that the product from dimerization of 200 shows one non-aromatic proton signal in its ^1^H NMR spectra. This analysis suggests that dimerization of 200 to form a radialene without incorporation of the aryl endgroups is unlikely. Although the exact structure of the product remained unclear, the formation of a naphthalene-containing moiety was proposed by Fischer (*vide infra*). In 2009, the structure of the product was proposed by Iyoda as the dimer 1,2-dihydrocyclobuta[*b*]naphthalene 201, formed *via* thermal dimerization of 200 in benzene under reflux to give an intermediate, which then underwent a 6π-electrocyclization followed by a proposed H-shift (Scheme 44a).^221^ In a subsequent synthetic report by Tykwinski and coworkers in 2023, the [4]cumulene 200 was isolated in *ca.* 42% yield and 95% purity. Attempts to further purify 200 through recrystallization led to the formation of 201 (Scheme 44a), which was confirmed through spectroscopic methods as well as X-ray crystallography.^211^ Interestingly, the [2 + 2] dimerization of 200 occurred in a head-to-head manner, similar to the syn-orientation determined by Stang and coworkers for odd cumulenes.^222^

Increasing the steric bulk of terminal endgroups has been investigated as a strategy to suppress the intermolecular dimerization pathway, utilizing the sterically bulky (3,5-di-*tert*-butyl)phenyl group. The synthesis of [4]cumulene 202, bearing a combination of phenyl and (3,5-di-*tert*-butyl)phenyl endgroups, has been attempted through the exhaustive elimination of HBr from the 1,4-diene 203 (Scheme 44b). However, this approach yielded a complex mixture of products, and the formation of 202 was not observed. From this mixture, the dimeric product 204 was isolated and identified by X-ray crystallography. It remains unclear whether 204 formed through trapping of an intermediate during the elimination of 203 or during purification of the crude reaction mixture. The instability of 202, compared to the isolable 200, is attributed to the increased electron density introduced by the (3,5-di-*tert*-butyl)phenyl endgroups, which offsets the potential stabilization provided by steric shielding.

Since two (3,5-di-*tert*-butyl)phenyl endgroups are insufficient for stabilization, [4]cumulene 205, bearing four bulky substituents, has been targeted and synthesized. Cumulene 205 dimerizes when heating a sample to 90 °C in dry toluene, as revealed by ^1^H NMR spectroscopy. Similar to the thermal dimerization of 200, the dimerization of 205 result in the formation of a head-to-head dimer 206, as confirmed by X-ray crystallographic analysis (Scheme 44a), albeit in only trace amounts.

In the 1960s, extended unsaturated carbenes, such as alkylidenes and vinylidenes, were extensively studied through the trapping of reaction intermediates.^223^ For example, Stang and coworkers have investigated the reactivity of vinylidene carbenes through the addition reactions to olefins, yielding cumulenic products. From both mechanistic and synthetic perspectives, carbene trapping has emerged as a valuable approach for advancing the understanding of cumulene chemistry.^66,222^

**Scheme 45:**
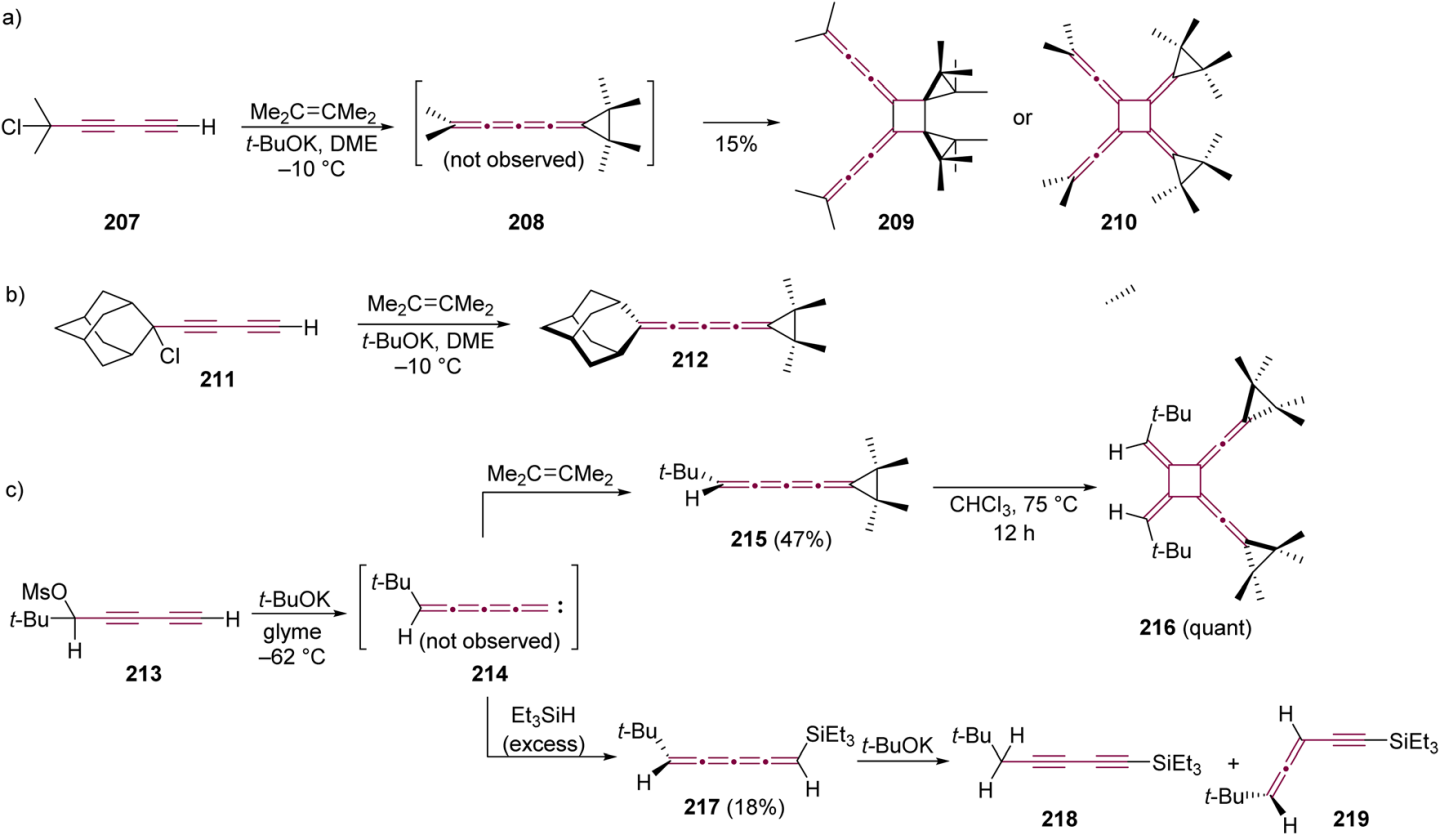
Synthesis and subsequent reactions of [4]cumulenes based on trapping of vinylidene carbenes bearing (a) methyl 207, (b) adamantyl 211, and (c) *t*-Bu 213 endgroups.^224–226^

Le Noble and coworkers have explored a carbene trapping method to form an even [4]cumulene, through base-induced elimination of the diyne precursor 207 and tetramethylethene as the trapping agent (Scheme 45a).^224,225^ The [4]cumulene 208 could not be isolated, and a dimer was obtained as the only product. The head-to-head dimerization was proposed based on earlier findings by Stang and coworkers,^222^ and among the two possible isomers (209 and 210), the former was deemed more consistent with the spectroscopic data. Interestingly, replacing the methyl endgroups with a bulkier adamantylidene group in a similar carbene trapping experiment with 211 yielded the stable [4]cumulene 212, which did not undergo cyclooligomerization (Scheme 45b).^225^

Stang and Learned, in 1988, reported on the reactivity of a vinylidene carbene system terminated at one end with a single *tert*-butyl group (Scheme 45c).^226,227^ Starting from diyne 213, elimination at a low temperature (−62 °C) produces carbene 214, which is trapped with tetramethylethene to afford the [4]cumulene 215 in 47% yield. Cumulene 215 is relatively unstable and undergoes cyclodimerization quantitatively to form 216 after several days at room temperature or a few hours upon heating in chloroform.^227^ Alternatively, trapping 214 with Et_3_SiH yields the unstable [4]cumulene 217 in 18% yield. In the presence of *t*-BuOK, 217 undergoes a prototropic rearrangement to give a mixture of diyne 218 and enyne 219 (Scheme 45c).

### Cycloaddition reactions

**Scheme 46:**
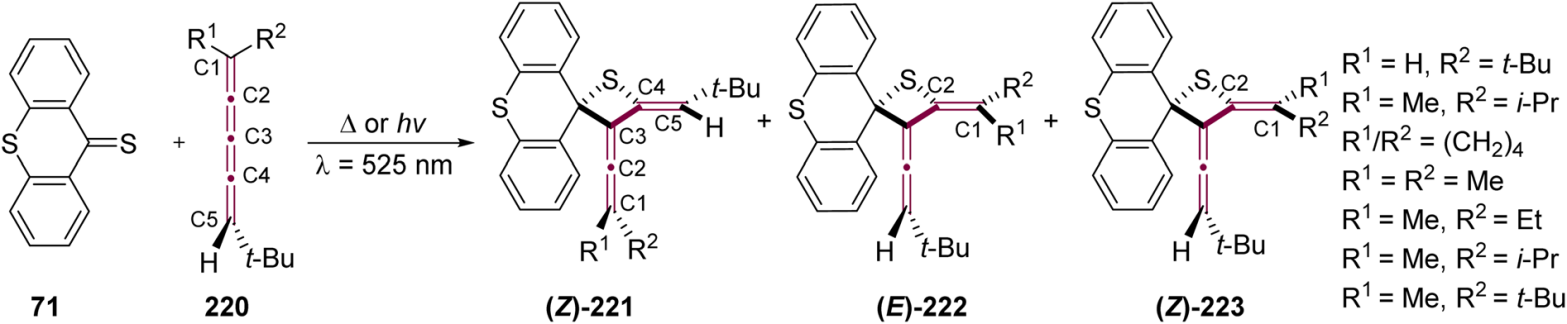
Thermal and photochemical [2 + 2] cycloaddition of thioxanthenethione 71 with [4]cumulenes 220.^228^

Visser and coworkers have studied a series of photochemical and thermal [2 + 2] cycloaddition reactions between thioxanthenethione 71 and [4]cumulenes 220 (Scheme 46).^228^ The thermal reactions were conducted using a solution of 71 (0.02 M) and 220 at room temperature and were typically completed within 15 minutes. To prevent competition from the rapid thermal reaction, the photochemical reactions were performed in dilute solutions of 71 (0.005 M) at −70 °C. [4]cumulenes 220 bearing a range of endgroups and substitution patterns were investigated, and the reactions consistently produced three isomeric thietanes: (*Z*)-221, (*E*)-222, and (*Z*)-223. Notably, in contrast to earlier photochemical studies on odd [*n*]cumulenes that show reactivity at C1 and C2, the thiocarbonyl functional group in its triplet state preferentially attacks position C2 or C4 of the [4]cumulenes. Interestingly, the size of the substituent does not significantly affect the product ratios under photochemical conditions. Under thermal conditions, however, steric hindrance from the substituents plays a dominant role in determining the product distribution.^228^

**Scheme 47:**
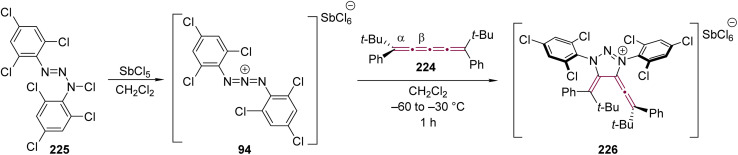
Reaction of [4]cumulene 224 with 1,3-diaza-2-azoniaallene salt 94.^118^

In 1997, a [3 + 2] cycloaddition reaction was reported by Jochims and coworkers using [4]cumulene 224 and the 1,3-diaza-2-azoniaallene salt 94, formed *in situ* by reacting antimony pentachloride with *N*-chlorotriazine 225 (Scheme 47).^118^ The salt 94 was added to a solution of [4]cumulene 224 in CH_2_Cl_2_, producing a red suspension *via* the [3 + 2] cycloaddition reaction and, upon purification, the triazolium salt 226 was isolated. X-ray crystallographic analysis revealed that the cycloaddition proceeded regioselectively at one of the β-bonds of the [4]cumulene moiety. The formation of triazolium salt 226 was calculated to be exothermic by 40 kcal mol^−1^, which is markedly higher than 18 kcal mol^−1^ for the corresponding cycloaddition reaction of 94 with analogous [3]cumulene 95. This finding suggests a more thermodynamically favorable product for the reaction with [4]cumulenes, although the overall reactivity would also depend on the activation barrier, which is not explicitly addressed in this study. A comparison of the reactivity of salt 94 with both [4]cumulene 224 and [3]cumulene 95 (Scheme 20) raises intriguing questions regarding the steric and electronic factors that govern cycloaddition regioselectivity in cumulenes. Further investigations into alternative reaction conditions and substituent effects may provide a more comprehensive understanding of the reactivity of cumulenes.

### Organometallic reactions

Before the advent of advanced spectroscopic techniques, hydrogenation was widely employed to identify the presence and number of non-aromatic double bonds in cumulenes.^219^ [4]cumulenes, which contain four contiguous double bonds, can undergo complete hydrogenation to carbon–carbon single bonds with consumption of four molar equivalents of hydrogen.^219,229^ In most of this section, we discuss the hydrogenation of [4]cumulenes using RANEY® and palladium catalysts to confirm their constitutional molecular formulas. We also highlight the correlation between the reaction time and the impact of various endgroups on the hydrogenation process. Finally, we examine the partial hydrogenation of [4]cumulenes with Lindlar's catalyst and aluminum amalgam, illustrating the formation of intriguing side products.

**Scheme 48:**
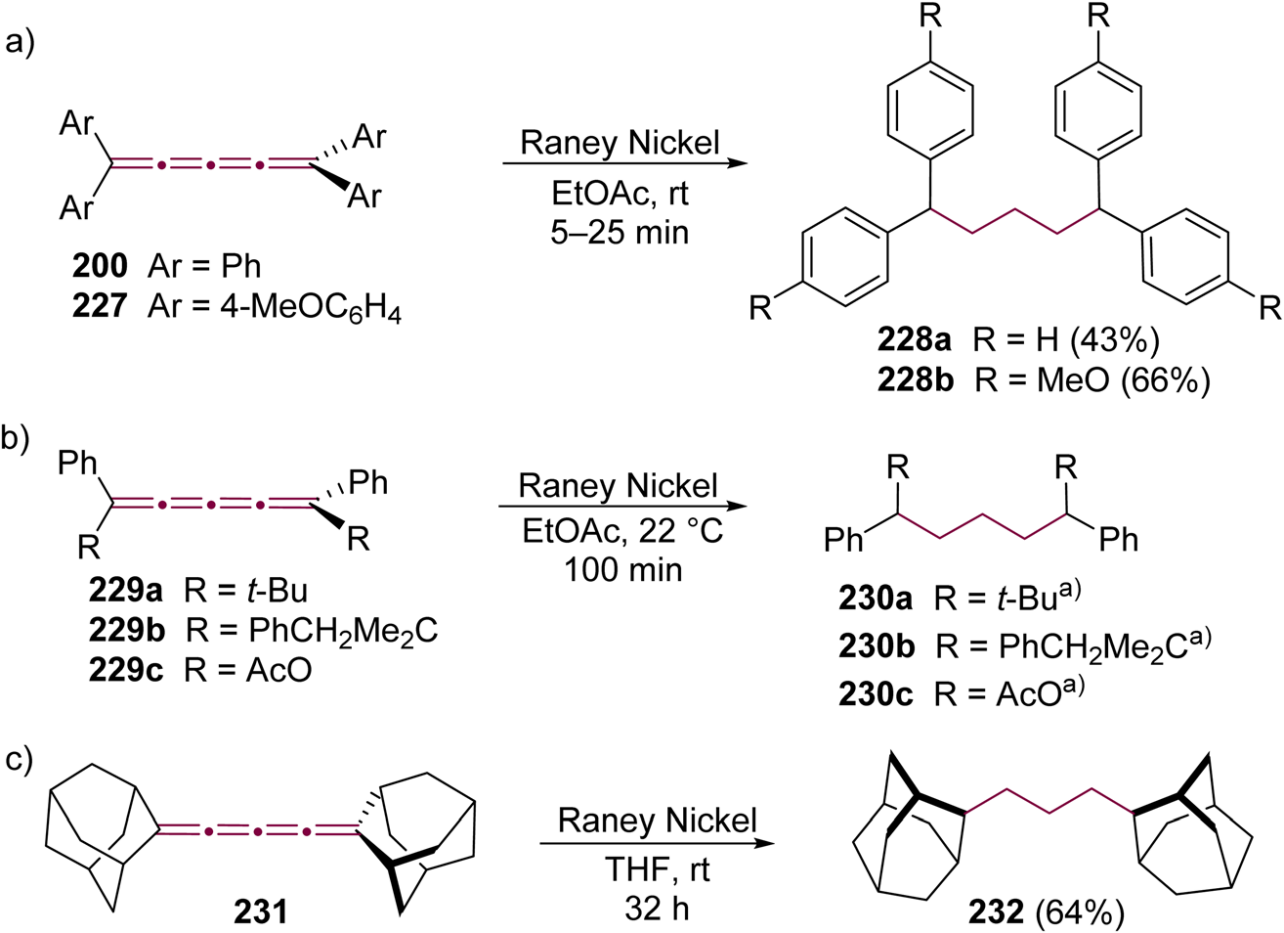
Hydrogenation reaction with RANEY® on [4]cumulenes: (a) 200 and 227, (b) 229a–c, and (c) 231; ^a)^ – yield not given.^219,229,230^

To the best of our knowledge, the first hydrogenation of even [*n*]cumulenes featuring aryl endgroups (200 and 227) was reported in 1964 by Kuhn and Fischer.^219^ When these [4]cumulenes are treated with RANEY® in ethyl acetate, approximately four equivalents of hydrogen are consumed in each case, yielding tetraarylpentanes 228a and 228b in 43 and 66% yields, respectively, within 5–25 minutes (Scheme 48a). Subsequently, in 1977, Karich and Jochims investigated the reduction of [4]cumulenes bearing aryl and bulky alkyl endgroups (229a,b, Scheme 48b),^229^ while in 1986 Nader and Brecht examined a [4]cumulene bearing both aryl and carboxylate endgroups (229c, Scheme 48b).^230^ In all these cases, the results mirror those of Kuhn and Fischer, with [4]cumulenes 229a,^190^229b,^229^ and 229c^230^ each consuming approximately four equivalents of H_2_ to produce the corresponding 1,1,5,5-tetrasubstituted pentanes 230a–c, albeit the time necessary for full hydrogenation is extended to 30–100 min (Scheme 48b). A comparison of these studies suggests that bulky alkyl endgroups likely shield the cumulene core, thereby slowing the hydrogenation reactions.

Karich and Jochims described the hydrogenation of adamantylidene-endcapped [4]cumulene 231, demonstrating the effect of fully replacing aryl endgroups with an alkyl substituent (Scheme 48c).^229^ They observed that the reactivity of 231 decreases significantly, with the reaction requiring 32 hours to yield the fully hydrogenated product 232. The authors attributed the reduced reactivity, in part, to the low solubility of 231 in THF, which may have hindered the reaction speed. If one considers the progression from 200/227 (5–25 min) to 229 (100 min) to 231 (32 h), however, it appears equally plausible that the alkyl substitution itself contributes to the slower reaction rate.

**Scheme 49:**
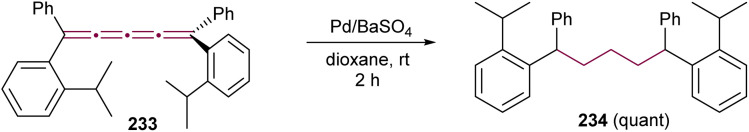
Hydrogenation of [4]cumulene 233 using Pd/BaSO_4_.^231^

In 1977, von Herrath and Rewicki demonstrated the hydrogenation of cumulene 233 to 1,1,5,5-tetrasubstituted pentane 234 using Pd/BaSO_4_ (Scheme 49).^231^ Compared to tetraaryl[4]cumulenes 200 and 227, which are fully hydrogenated within 25 min, the reaction with Pd/BaSO_4_ requires two hours to fully convert the product, also with a quantitative yield.

**Scheme 50:**
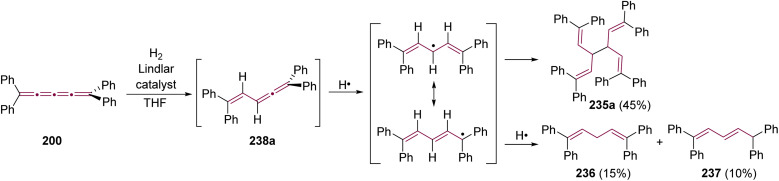
Hydrogenation of 200 with Lindlar's catalyst.^218^

In 1964, Fischer and Fischer reported a partial hydrogenation of 200 using Lindlar's catalyst.^218^ The reaction consumes approximately 1.9 equivalents of H_2_, rather than the four equivalents required for full hydrogenation using Pd/BaSO_4_ or RANEY®. Under these conditions, the major product arises from reductive dimerization to yield 235a (45% yield), accompanied by the minor diene products 236 and 237 in 15% and 10% yields, respectively (Scheme 50). The authors proposed a mechanism involving the initial formation of intermediate 238a, which then undergoes addition of an H˙ radical to produce an intermediate radical species. This radical preferentially dimerizes to form 235a, while a small proportion undergoes H˙ abstraction, leading to the formation of dienes 236 and 237 (Scheme 50).

**Scheme 51:**
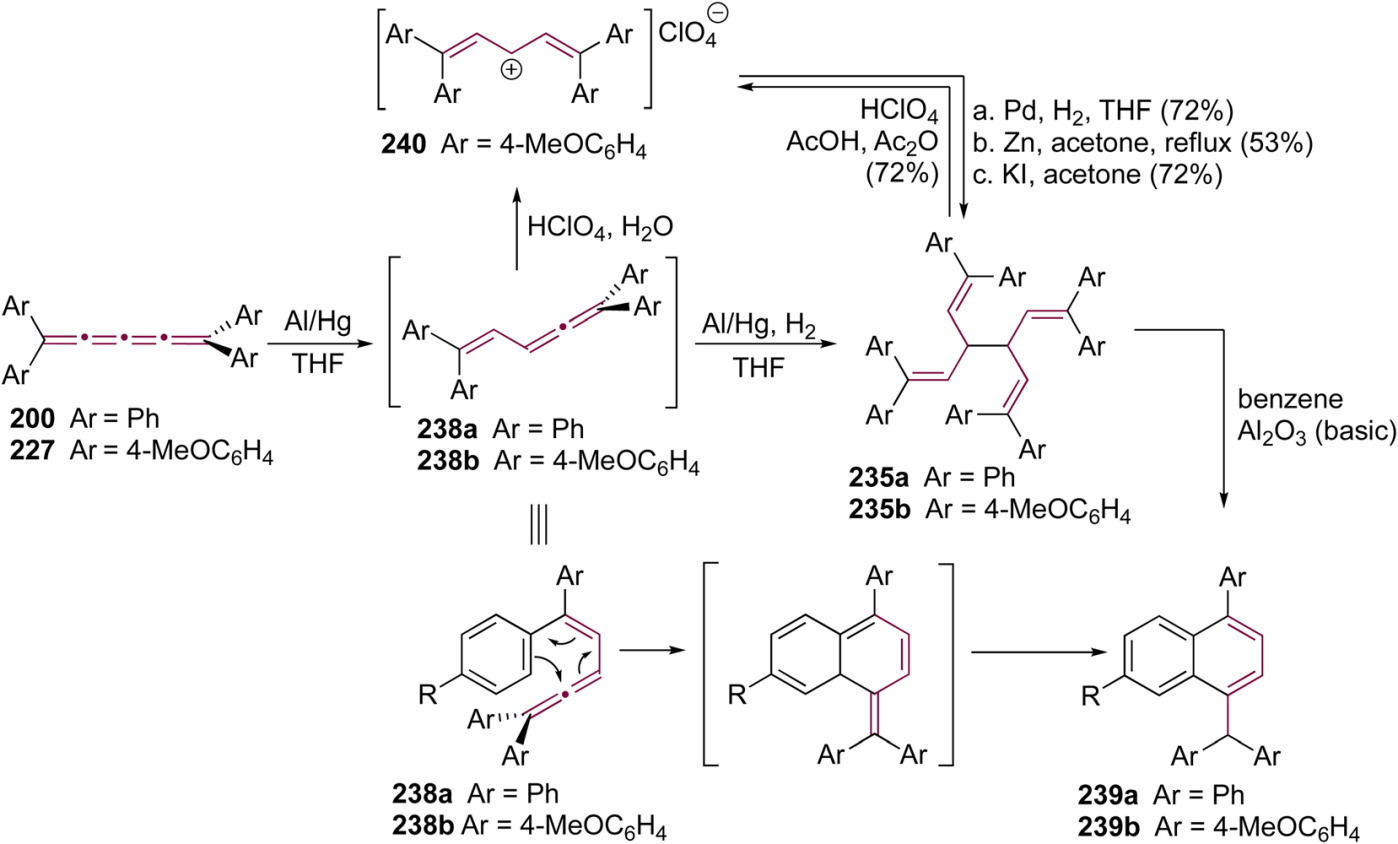
Hydrogenation reaction of [4]cumulenes 200 and 227 with Al/Hg amalgam.^218^ Oxidation of 238b with HClO_4_ and subsequent dimerization to 235b.^232^ Proposed mechanism for the formation of naphthalenes 239a,b.^218^

Fischer has explored the species involved in the reduction of 200 and 227 using Al/Hg amalgam (Scheme 51).^218^ Attempts to isolate the intermediates 238a and 238b from Al/Hg reduction *via* column chromatography (Al_2_O_3_) instead yield the colorless naphthalene derivatives 239a and 239b (Scheme 51). These compounds are postulated to arise from triene intermediates 238a and 238b, respectively, which undergo a 6π-electrocyclization followed by a proposed [1,7]-hydride shift. This transformation closely resembles the mechanism previously proposed for the formation of 201 from [4]cumulene 200 (Scheme 44).

On the other hand, Wizinger and coworkers reported that treating 238b with perchloric acid produces the oxidized species 240 as a blue solid.^232,233^ Subsequent reduction of 240 yields the dimer 235b as the predominant product regardless of the conditions employed. This transformation resembles the formation of 235a from 238a previously reported by Fischer using Lindlar's catalyst (Scheme 50).^218^ Notably, the formation of the dimer 235b is reversible, *i.e.*, treating 235b with perchloric acid in acetic acid and acetic anhydride regenerates the oxidized salt 240 in 72% yield.

**Scheme 52:**
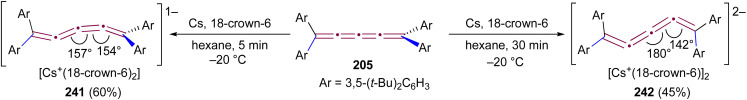
Chemical reduction of [4]cumulene 205 with cesium metal to form mono- and dianions (bonds for calculating the torsion angle *Φ* are shown in blue).^234^

The first successful isolation of the reduced species formed from a [4]cumulene was reported by Petrukhina, Tykwinski, and coworkers in 2024. The authors explored the redox characteristics of 205 using cesium metal as a reducing agent and observed the formation of both a monoanionic radical and a dianionic species.^234^ The authors noted that the reduced species obtained from the reaction of 205 with either sodium or potassium metal is too reactive for isolation. Crystallographic data reveal that, upon stepwise reduction, the [4]cumulene undergoes significant geometric changes that include twisting of the endgroups from an orthogonal geometry in 205 toward planarization in mono- and dianions 241 and 242, respectively (Scheme 52). Using X-ray structural analysis, the torsion angle between opposing endgroups (blue bonds in Scheme 52) is reduced from *Φ* = 72° in neutral 205 to *Φ* = 42° in the monoanion 241 to *Φ* = 0° (*i.e.*, coplanar) in the dianion 242. These findings suggest important implications for the electron-handling capabilities of cumulenes, particularly in the context of redox-active materials.

### Miscellaneous reactions

**Scheme 53:**
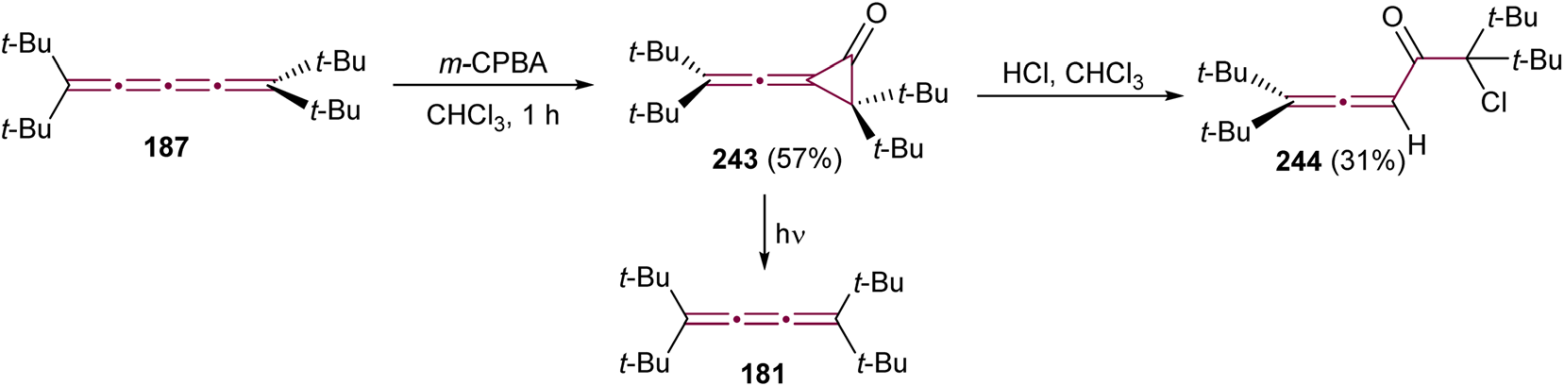
Oxidation of [4]cumulene 187 with *m*-CPBA.^187^

Crandall and coworkers have examined the reactivity of [*n*]cumulenes with *m*-CPBA and dimethyldioxirane (DMDO), proposing that the initially formed (not isolated) epoxide rapidly isomerizes to a relatively stable cyclopropenone intermediate. In one example, the reaction of tetra(*tert*-butyl)[4]cumulene 187 with one equivalent of *m*-CPBA yields the cyclopropenone intermediate 243 (Scheme 53).^187^ Over time, exposure of 243 to sunlight induces slow decarbonylation, affording the [3]cumulene 181. Alternatively, treatment of 243 with hydrochloric acid furnishes the allenyl ketone 244.

**Scheme 54:**
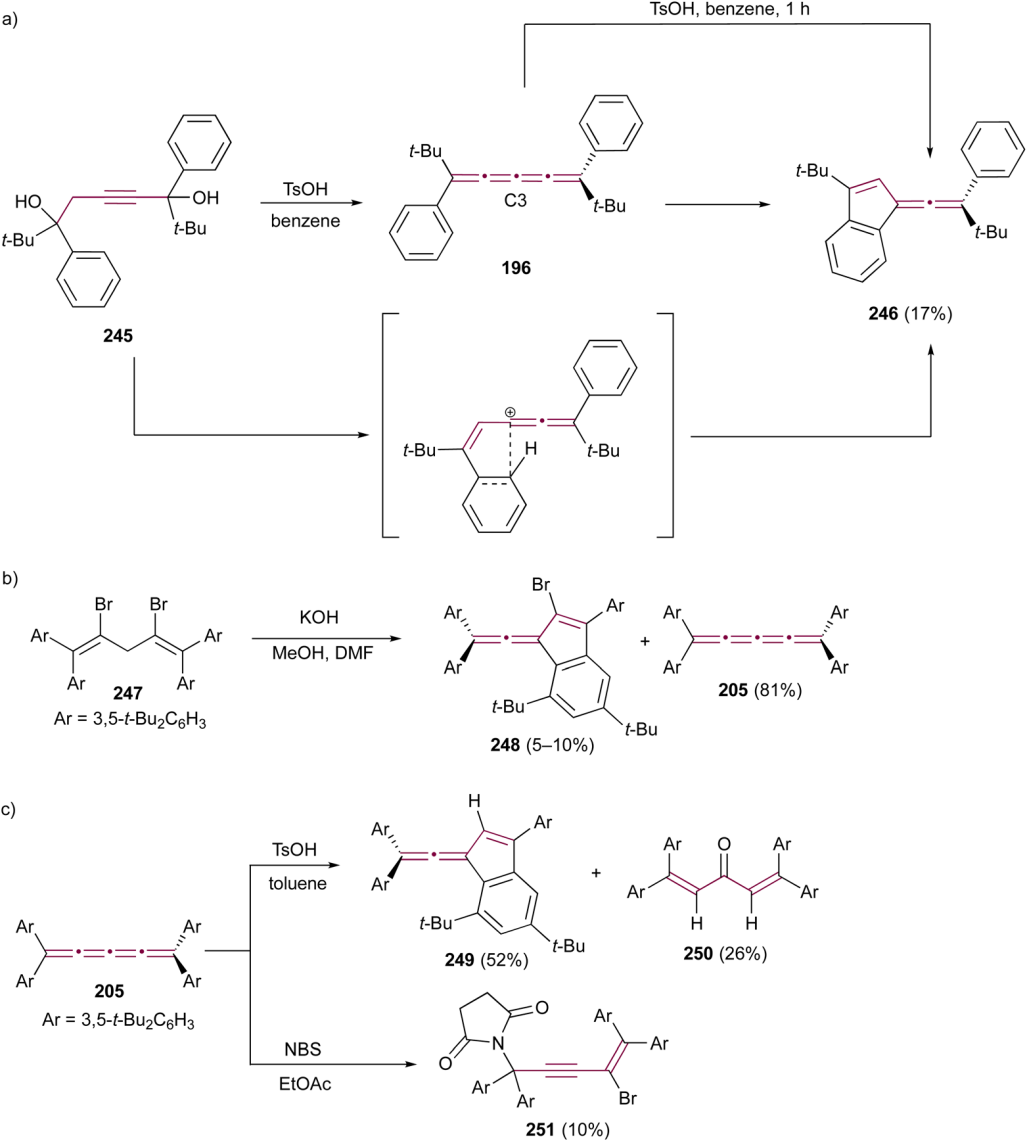
Reactions of (a) [4]cumulene 196 with TsOH, (b) formation of 248, and (c) reactions of [4]cumulene 205 with TsOH or NBS.^190,211,235^

Nakagawa and coworkers reported the synthesis of [4]cumulene 196 through dehydration of diol 245 using *p*-toluenesulfonic acid in benzene under reflux (Scheme 54a). The resulting yellow crystalline solid was initially proposed to be 196. However, subsequent hydrogenation experiments revealed the reduction of only three double bonds, inconsistent with the proposed structure of 196.^235^ In 1962, Kuhn and Schulz independently reported the synthesis of indene 246,^236^ which showed hydrogen consumption and spectral data consistent with the molecule described by Nakagawa. This finding strongly suggests that a structural revision is needed for Nakagawa's proposed product. While the hydrogen consumption and spectra of 246 aligned with Nakagawa's final product, the revision depended primarily on indirect data, given that no [4]cumulenes were yet known at the time. Attempts by Kuhn and Schulz to prepare [4]cumulene 196 in 1963 were unsuccessful.^237,238^ A breakthrough was noted in 1974, when Jochims and Karich successfully synthesized [4]cumulene 196*via* carbene insertion followed by dehalogenation, marking the first successful isolation of this species.^190,229^ In the same study, treating 196 with TsOH triggered intramolecular cyclization at the *ortho*-position of the phenyl endgroup and C3 of the cumulene chain (Scheme 54a).^190^ The resulting yellow crystalline solid was characterized as the indene structure 246, consistent with the findings of Kuhn and Schulz.

Indene formation was documented in 2023 for the sterically encumbered [4]cumulene 205 when treated with TsOH in toluene.^211^ During the synthesis of 205, the elimination reaction of divinylbromide 247 generated both the desired [4]cumulene 205 and the brominated indenylidene 248 (Scheme 54b). Because it is difficult to envision a plausible mechanism for formation of 248 under the strongly basic conditions used to eliminate HBr from 247, the authors proposed that 248 is most likely produced by bromination of 205 during the workup process.

In analogy to the formation of 246 from 196, subjecting [4]cumulene 205 to TsOH induced intramolecular cyclization, giving 249, along with 250, which arose from the acid-catalyzed hydration of 205 (Scheme 54c). Notably, the presence of acid is essential, as stirring 205 in a mixture of acetone and water (1 : 1) at room temperature did not yield either 249 or 250. Although not explored in detail, an attempt to brominate 205 with the mild brominating agent NBS did not reportedly provide 248; instead, 251 was obtained and characterized by X-ray crystallography.

**Scheme 55:**
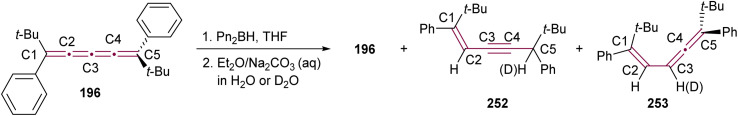
Hydroboration of [4]cumulene 196 with Pn_2_BH (Pn = (+)-α-pinene).^239^

Jochims and coworkers have reported the partial hydroboration of [4]cumulene 196 with di(3-pinanyl)borane (Pn_2_BH), toward enantioselective enrichment *via* kinetic resolution. Treating 196 with a substoichiometric amount of Pn_2_BH (0.67 equivalents) returns 33% of unreacted 196 with approximately 5% enantioenrichment, presenting a potential approach to enantiomeric resolution of the [4]cumulene scaffold. Enyne 252 is isolated as the major product and triene 253 as the minor product (Scheme 55).^239^ Deuteration experiments reveal that boronation occurs at C3 of the cumulene core, while hydride is delivered to C2. Subsequent quenching with H_2_O or D_2_O introduces a proton or deuteron at C5 (yielding 252) or C3 (yielding 253).

**Scheme 56:**
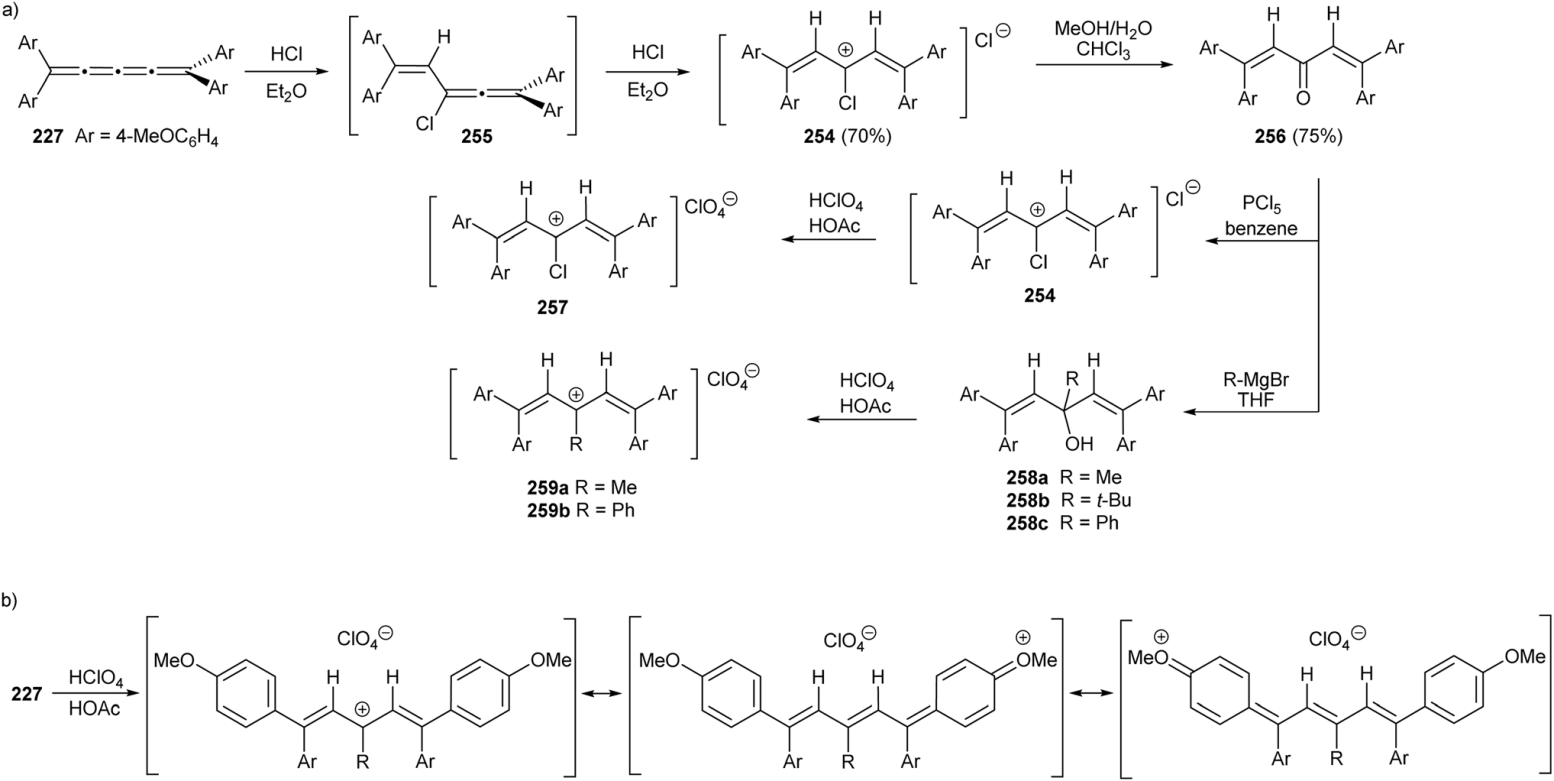
(a) Reaction of [4]cumulene 227 with HCl and (b) resonance stabilization of the salt 259 proposed by Fischer.^218^

Tetraphenyl[4]cumulene 200 reacts in the presence of either hydrochloric or formic acid, but the hydrolyzed product is not sufficiently stable for characterization (not shown). Fischer and Fischer report that treating [4]cumulene 227 with two equivalents of HCl yields black-violet crystals identified as the pentadienyl salt 254 (Scheme 56a).^218^ They hypothesized that the reaction proceeds *via* a pentatriene intermediate 255, formed by the addition of one equivalent of HCl to [4]cumulene 227, although 255 is not isolated. Subsequent treatment of pentadienyl salt 254 in chloroform with a mixture of methanol and water (9 : 1) gives colorless crystals of pentadieneone 256 in 75% yield. Treating 256 with PCl_5_ in benzene gives a deep violet solution whose UV-vis spectrum matched that of 254, and its further reaction with perchloric acid produces the brown-violet perchlorate salt 257. Alternatively, the reaction of pentadieneone 256 with various Grignard reagents yields alcohols 258a–c, which subsequently form the corresponding perchlorate salts 259a,b upon reaction with perchloric acid. The authors suggested that the phenyl endcapped derivatives lack the resonance stabilization provided by anisole endgroups, thus accounting for the reduced stability (Scheme 56b).

**Scheme 57:**
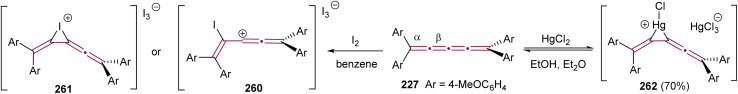
Iodination and mercuriation of [4]cumulene 227.^218^

Treating tetraanisyl[4]cumulene 227 with iodine yields a gray-black crystalline precipitate proposed to be the allenic cation 260 (Scheme 57). Iodination at the β-bond is suggested on the basis of IR and UV-vis analyses, as well as comparisons with results from the protonation reactions (*vide supra*). Although not explicitly mentioned in the study, it is worth considering that the structure takes the form of an iodonium intermediate 261.^240^ Intermediates or products from the analogous halogenation reactions of [4]cumulene 227 using Br_2_ could not be characterized, although it is noted that three molar equivalents of Br_2_ are consumed during the bromination.

Similar to reactions of odd [*n*]cumulenes with HgCl_2_, treating tetraphenyl[4]cumulene 200 with HgCl_2_ reportedly fails to produce stable products that could be either isolated or studied.^218^ In contrast, the reaction of 227 with HgCl_2_ immediately yields a purple crystalline precipitate in 70% yield that is identified as complex 262 formed *via* addition to the β-bond (Scheme 57). Spectroscopic analyses confirm that 262 is structurally similar to 261, exhibiting essentially identical IR and UV-vis absorption characteristics. The formation of 262 is reversible, and shaking 262 in a water or alcohol solution regenerates 227, which crystallizes out of the solution.^218^

The combined studies by Fischer and Fischer reveal key reactivity trends, particularly regarding protonation reactions.^218^ Most notably, allenes and even [*n*]cumulenes (*n* = 4) exhibit enhanced reactivity compared to odd [*n*]cumulenes (*n* = 3 and 5). Based on the evidence in hand, it also seems clear that the β-bond in [4]cumulenes is more reactive than the terminal α-bond. Fischer and Fischer rationalize this trend based on the loss of π-electron energy upon, *e.g.*, protonation. This analysis clearly shows that the energy loss is more significant for even than odd [*n*]cumulenes (Table 1). While not reported in the study, this interpretation could easily be extended to other electrophilic reactivity patterns that show preference for position C2. Thus, the experimental outcomes align with the hypothesis that the enhanced reactivity of the β-bond stems from its lower reorganization energy, leading to a preference for this bond in the reaction pathway and ultimately producing a stable product.

**Table 1:** Relative π-electron energies of tetraphenyl[*n*]cumulenes and their conjugate acids^218^

Cumulene/conjugate acid	Total π-electron energy	π-Electron energy loss resulting from protonation
Ph_2_C=C=CPh_2_/Ph_2_C=CH–^+^CPh_2_	36.88/35.90 β	0.98 β
Ph_2_C=C=C=CPh_2_/Ph_2_C=C=CH–^+^CPh_2_	39.38/37.82 β	1.56 β
Ph_2_C=C=C=C=CPh_2_/Ph_2_ C=C=C=CH–^+^CPh_2_	41.88/40.80 β	1.08 β
Ph_2_C=C=C=C=C=CPh_2_/Ph_2_ C=C=C=C=CH–^+^CPh_2_	44.40/42.90 β	1.50 β

**Scheme 58:**
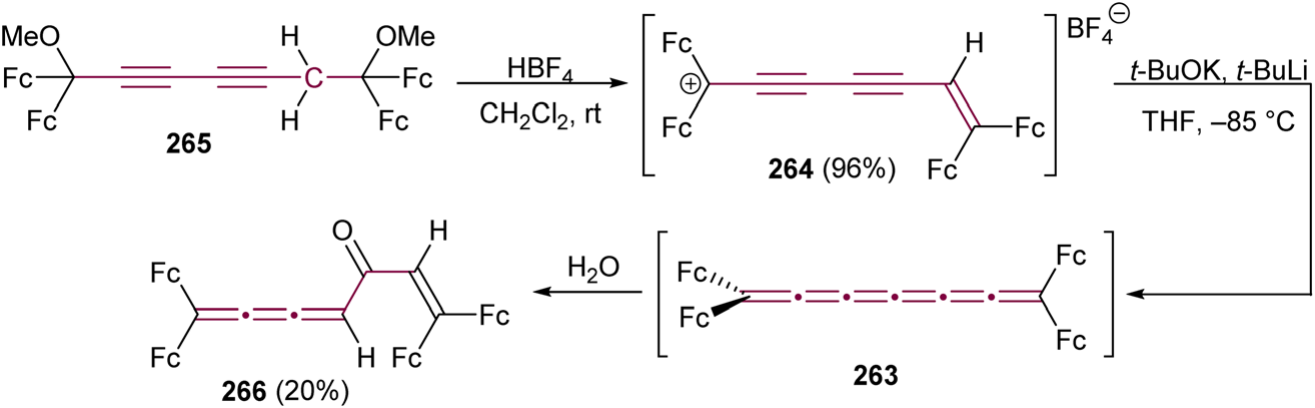
*In situ* formation of [6]cumulene 263 followed by hydrolysis to give 266 (Fc = ferrocenyl).^217^

Although the isolation and characterization of a [6]cumulene have not yet been reported, a single study by Bildstein and coworkers from 2001 describes the *in situ* formation of tetraferrocenyl[6]cumulene 263 (Scheme 58).^217^ Their efforts to establish the formation of 263 include the elimination reaction of diyne 265 to form the cation 264, followed by a trapping experiment with H_2_O, giving 266 as the product.

## Conclusions

Over the past century, the chemistry of [*n*]cumulenes has witnessed the development of a variety of synthetic methodologies that allow the construction of these “exotic” molecules. Historically, much of the motivation has stemmed from studying [*n*]cumulenes as models of carbyne-like systems or from harnessing their unique electronic properties. However, the robust synthetic protocols now available, particularly for [3]- and [5]cumulenes, highlight that these systems can also serve as remarkably versatile building blocks in organic synthesis.

Despite the challenges that arise when attempting to access longer odd (beyond [5]cumulenes) and even [*n*]cumulenes, the chemistry surveyed in this review demonstrates that these compounds are far from being mere curiosities. Indeed, the high internal energy inherent in [*n*]cumulenes leads to reactivity profiles that can unlock new transformations and provide innovative routes to frameworks otherwise difficult to obtain. From cyclooligomerization and cycloaddition processes to organometallic transformations, [*n*]cumulenes repeatedly display a capacity to form carbon skeletons that are largely inaccessible from more conventional substrates.

We hope that this comprehensive overview will convince the broader organic chemistry community to consider [*n*]cumulenes as useful synthetic building blocks. By incorporating cumulene moieties into retrosynthetic plans, chemists can exploit their unusual reactivity to generate novel architectures and potentially uncover uncharted reaction pathways. In this way, [*n*]cumulenes offer a potent combination of structural diversity, synthetic accessibility (especially for the shorter members), and distinctive chemical behavior, an attractive toolkit that deserves wider recognition and application in contemporary organic synthesis.

## Author contributions

All authors contributed to writing and editing this article.

## Conflicts of interest

The authors declare no competing interests.

## Data Availability

No primary research results, software or code have been included and no new data were generated or analysed as part of this review.
